# Ultrasound-Assisted Synthesis of Substituted Chalcone-Linked 1,2,3-Triazole Derivatives as Antiproliferative Agents: In Vitro Antitumor Activity and Molecular Docking Studies

**DOI:** 10.3390/ijms26073389

**Published:** 2025-04-04

**Authors:** Manuel Cáceres, Víctor Kesternich, Marcia Pérez-Fehrmann, Mariña Castroagudin, Ronald Nelson, Víctor Quezada, Philippe Christen, Alejandro Castro-Alvarez, Juan G. Cárcamo

**Affiliations:** 1 Departamento de Química, Facultad de Ciencias, Universidad Católica del Norte, Avda. Angamos 0610, Antofagasta 1270709, Chile; manu.caceresv@gmail.com (M.C.); maperez@ucn.cl (M.P.-F.); marina.castroagudin@ce.ucn.cl (M.C.); rnelson@ucn.cl (R.N.); victor.quezada@ucn.cl (V.Q.); 2 School of Pharmaceutical Sciences and Institute of Pharmaceutical Sciences of Western Switzerland, University of Geneva, 1205 Geneva, Switzerland; philippe.christen@unige.ch; 3 Departamento de Ciencias Preclínicas, Facultad de Medicina, Universidad de La Frontera, Temuco 4811230, Chile; 4 Instituto de Bioquímica y Microbiología, Facultad de Ciencias, Universidad Austral de Chile, Valdivia 5091000, Chile; gcarcamo@uach.cl; 5 Centro FONDAP, Interdisciplinary Center for Aquaculture Research (INCAR), Valdivia 5091000, Chile

**Keywords:** synthesis of 1,2,3-triazole, chalcone, antitumor activity, molecular docking studies

## Abstract

The synthesis of (*E*)-1-(1-benzyl-5-methyl-1*H*-1,2,3-triazol-4-yl)-3-phenyl-2-propen-1-one derivatives was carried out in two steps, using benzylic chloride derivatives as starting material. The structural determination of intermediates and final products was performed by spectroscopic methods: infrared spectroscopy, nuclear magnetic resonance spectroscopy and mass spectrometry (IR, NMR, and MS). In vitro evaluation of cytotoxic activity on adherent and non-adherent cells showed that triazole chalcones exhibited significant activity against three of the five cell lines studied: non-Hodgkin lymphoma U937, glioblastoma multiform tumor T98G, and gallbladder cancer cells Gb-d1. In contrast, the cytotoxic activity observed for cervical cancer HeLa and gallbladder adenocarcinoma G-415 was considerably lower. Additionally, in the cell lines where activity was observed, some compounds demonstrated an In vitro inhibitory effect superior to that of the control, paclitaxel. Molecular docking studies revealed specific interactions between the synthesized ligands and therapeutic targets in various cell lines. In U937 cells, compounds **4a** and **4c** exhibited significant inhibition of vascular endothelial growth factor receptor (VEGFR) kinase, correlating with their biological activity. This effect was attributed to favorable interactions with key residues in the binding site. In T98G cells, compounds **4r** and **4w** showed affinity for transglutaminase 2 (TG2) protein, driven by their ability to form hydrophobic interactions. In Gb-d1 cells, compounds **4l** and **4p** exhibited favorable interactions with mitogen-activated protein kinase (MEK) protein, similar to those observed with the known inhibitor selumetinib. In HeLa cells, compounds **4h** and **4g** showed activity against dihydrofolate reductase (DHFR) protein, driven by hydrogen bonding interactions and favorable aromatic ring orientations. On the other hand, compounds **4b** and **4t** exhibited no activity, likely due to unfavorable interactions related to halogen substitutions in the aromatic rings.

## 1. Introduction

**Figure 1:**
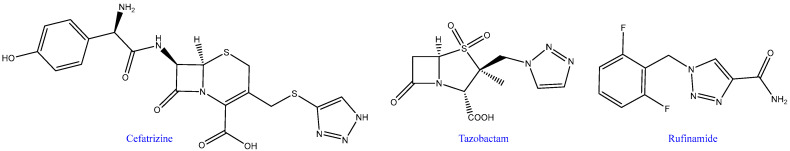
Drugs that possess a 1*H*-1,2,3-triazole ring in their structure.

The 1*H*-1,2,3-triazole ring is a stable and versatile structure widely used as a bioisostere, a pharmacophore, a linker, and a building block for synthesizing more complex chemical compounds, including pharmaceutical drugs [1,2]. Although the 1*H*-1,2,3-triazole ring does not directly correlate with biological activity, it is found in the structure of several drugs, such as cefatrizine, a cephalosporin antibiotic; tazobactam, a β-lactamase inhibitor; and rufinamide, an anticonvulsant [3]. The 1,2,3-triazole ring is a prominent pharmacophore system among nitrogen-containing heterocycles, and it can be easily synthesized using ‘click’ chemistry through copper- or ruthenium-catalyzed azide–alkyne cycloaddition reactions (Figure 1) [4].

In addition, the 1,2,3-triazole pharmacophore has been extensively reported in the medicinal chemistry literature for its diverse pharmacological profile. Some of its pharmacological activities include platelet antiaggregant [5,6], antitumor [7,8,9,10], anticonvulsant [11], potassium channel activator [12], antimicrobial [13] anti-inflammatory [14,15], antiviral [16,17,18], antichagasic [19,20], antituberculosis [21,22,23,24,25,26], antileishmanial [27], antibacterial [28], and antifungal [29] properties.

**Figure 2:**
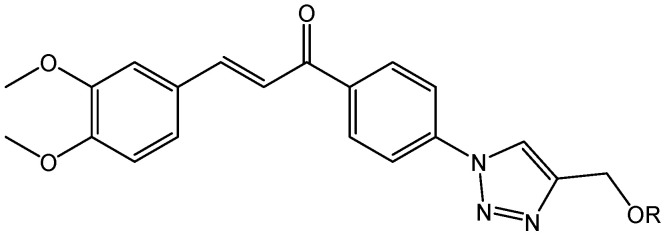
Neuroprotector chalcone triazole derivatives structure.

On the other hand, chalcones are α,β-unsaturated ketones that form the central core of numerous biologically active compounds and serve as precursors in flavonoid biosynthesis [30]. Structurally, they consist of two aromatic rings linked by a three-carbon α,β-unsaturated carbonyl system, which enables diverse chemical modifications and facilitates interactions with biological targets [31]. Furthermore, the pharmacological activities of chalcones are diverse, and are influenced by the position and type of substituents on their aromatic rings [32,33]. For instance, Mohammed et al. [34] reported the synthesis of 1,2,3-triazole-linked ciprofloxacin–chalcones, which exhibited remarkable antiproliferative activity against colon cancer cells. They attributed the antiproliferative effect to the presence of the 1,2,3-triazole ring, and the inhibitory activity on tubulin polymerization to the chalcone substructure moiety [34]. Zhang et al. reported the synthesis and antiproliferative activity of novel chalcone-1,2,3-triazole-azole hybrids, identifying a compound with exceptional efficacy against SK-N-SH cancer cells. Mechanistic investigations revealed that this compound induced morphological changes in cancer cells, likely through apoptosis activation, suggesting its potential as a therapeutic candidate for neuroblastoma treatment [35]. The hybrid framework, combining chalcone and triazole–azole pharmacophores, highlights a promising strategy for developing anticancer agents with enhanced activity profiles. Other analogous structures have also demonstrated efficient antitumor activity in vitro [36,37,38,39]. In addition to their anticancer properties, chalcones and their derivatives exhibit a wide range of pharmaceutical activities, including antibacterial, antifungal, antiparasitic, antioxidant, antimalarial, and antiviral activities, and anti-infective effects [40,41,42,43,44]. Furthermore, they have demonstrated efficacy as neuroprotectors against oxidative stress-induced neuronal cell damage (Figure 2) [45].

Motivated by the goal of discovering compounds with potent anticancer activity and the opportunity to further investigate their structure–activity relationships, this study focused on synthesizing substituted chalcone-linked 1,2,3-triazole derivatives by combining two widely studied pharmacophores. The anticancer activity of these compounds was evaluated against G-415 (Human gallbladder adenocarcinoma), Gbd1 (Human gallbladder adenocarcinoma), U-937 (human histiocytic lymphoma), HeLa (human cervix adenocarcinoma), and T98G (human glioblastoma multiforme) cell lines. Additionally, molecular docking studies were performed on VEGFR, TG2, MEK, and DHFR proteins to explore their potential mechanisms of action.

## 2. Results and Discussion

### 2.1. Synthesis of Chalcone Derivatives

**Scheme 1:**
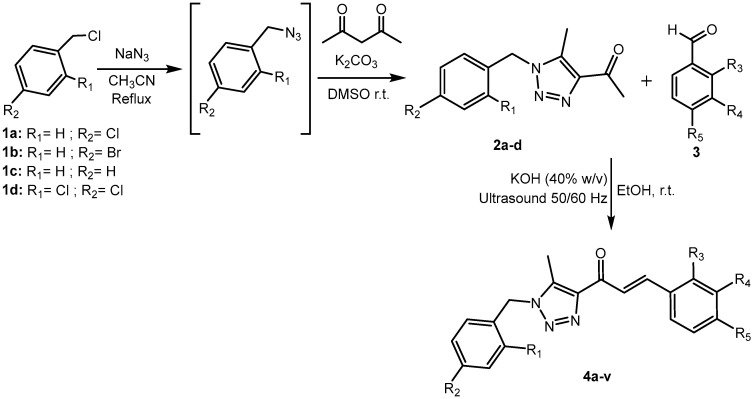
Synthesis of chalcones (**4a**–**v**).

For the synthesis of 1,2,3-triazole-linked chalcones, the Benzyl Acetyl Triazole derivatives (BAT) **2a**–**d** (Scheme 1) were first obtained as key intermediates. Their synthesis was carried out sequentially by substitution of benzyl chloride derivatives with NaN_3_ and subsequent cyclocondensation with acetylacetone in a basic medium, adapting procedures previously described by Chen et al. [46] and Nelson et al. [47]. Finally, the synthesis of the triazolic chalcone analogues **4a**–**v** was achieved using the method of Shankarling et al., involving a KOH (40% *w*/*v*)-promoted, ultrasound-assisted (frequency: 50/60 Hz) cross-aldol condensation between BAT **2a**–**d** and benzaldehyde derivatives (Scheme 1 and Table 1) [48]. 

The structural determination of the synthesized products was carried out through spectroscopic 1D and 2D NMR experiments, IR spectroscopy, and MS spectrometry. In the IR spectra of the chalcones, intense and sharp bands, attributable to the conjugated ketone carbonyl groups, were observed between 1684 and 1653 cm^−1^. In the ^13^C-NMR spectra, the carbonyl group was observed between 183.4 and 184.8 ppm (Table 2). Additionally, in the ^1^H-NMR spectra, two doublet signals were observed at approximately 8.31 and 7.78 ppm, both with a coupling constant (*J*) of 16 Hz (Table 2). This observation indicates the presence of vinyl protons conjugated to a ketone carbonyl group in a *trans*-configuration.

### 2.2. Molecular Docking

A molecular docking study was performed to identify the predominant interactions of the most active synthesized ligands in each of the cell lines studied. The analysis was organized according to the most representative therapeutic target for each cell line. The estimated energies were expressed in kcal/mol, and images were obtained using PyMol (Table 3).

#### 2.2.1. Therapeutic Target Involved in U937—VEGFR Kinase

**Figure 3:**
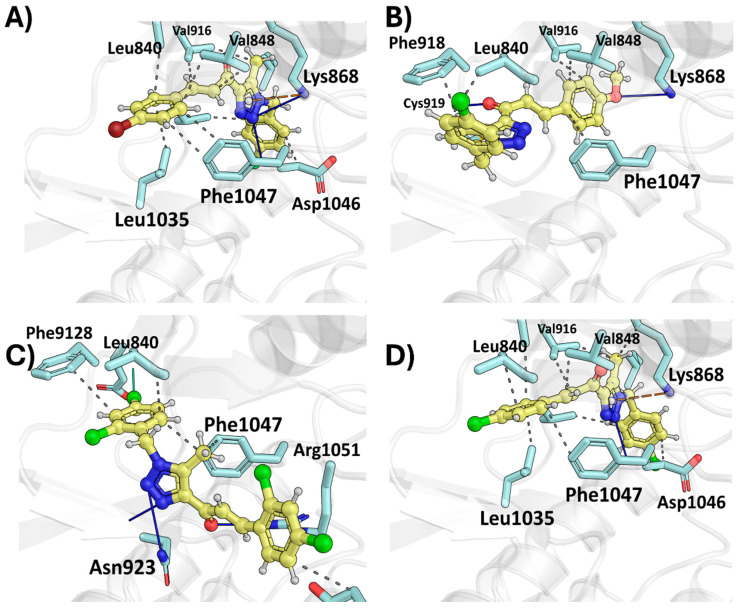
A representation of the best pose achieved for the most active compounds, **4a** (**A**) and **4c** (**B**), and the least active compounds, **4s** (**C**) and **4b** (**D**), on the VEGFR protein.

The VEFGR protein was selected as a target due to its reported overexpression in U937 and KG-1 cell lines [49]. One of the most relevant drugs for U937 is sorafenib, a multikinase inhibitor approved by the FDA, which has been shown to reduce the prevalence of refractory leukemia in its early stages [50]. VEFGR was obtained from the PDB (4ASD) [51]. The most active ligands, **4a** and **4c** (Figure 3A,B), were compared with the least active compounds, **4b** and **4s** (IC_50_ > 200 µM).

The docking poses were obtained by generating the top ten poses for each ligand. Compound **4c** (Figure 3B) presented favorable interactions due to its optimal phenyl arrangement, which enhanced the carbonyl orientation, allowing a favorable hydrogen bond with the Cys919 residue, which interacted with sorafenib (co-crystallized ligand). In addition, ligand **4c** oriented its phenylmethoxyl fragment towards the internal region of the binding site, establishing favorable interactions with Phe1047, Val91, and Val848. Furthermore, the chlorobenzyl group was positioned to form a π-stacking interaction with Phe918. In contrast, compound **4a** (Figure 3A) oriented its chlorobenzyl group towards the innermost pocket, interacting with Val848. The triazole fragment was positioned to form a hydrogen bond with the amide proton of residue Asp1046, which interacted with Leu840, Leu1035, and Phe1047 to accommodate the ligand and enhance its Van der Waals interactions. These interactions were also observed in sorafenib, explaining the strong biological activity and binding affinity of the ligands for the target site.

On the other hand, the low activity of compounds **4b** and **4s** (Figure 3C,D) may be attributed to the presence of chlorine atoms on the phenyl rings, which hindered the formation of favorable interactions within the internal pocket of the binding site. In the case of compound **4s**, its binding pose exposed the ligand to the solvent region of the binding site. In contrast, compound **4b** exhibited a binding mode similar to compound **4a**; however, the chlorophenyl group of the chalcone moiety does not engage in hydrophobic interactions as effectively as the bromobenzene group in **4a**.

#### 2.2.2. Therapeutic Target Involved in T98G—Tissue Transglutaminase, TG2

**Figure 4:**
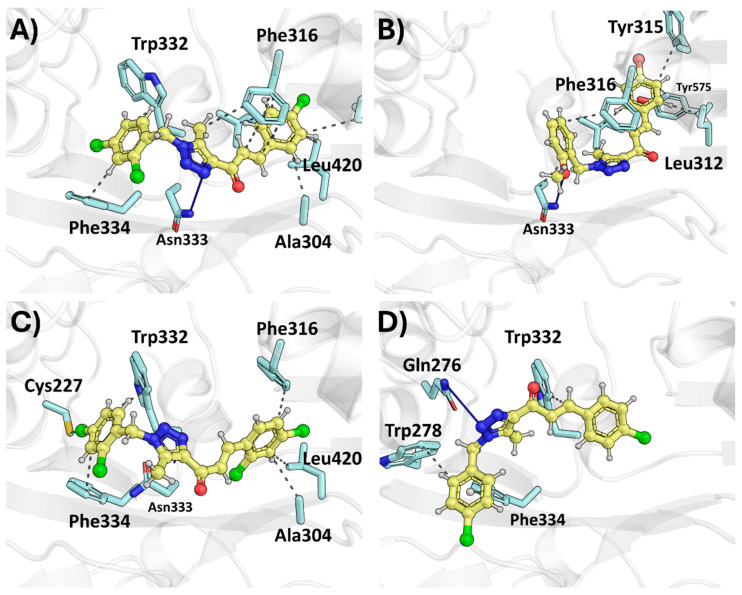
A representation of the best pose achieved for the most active compounds, **4q** (**A**) and **4v** (**B**), and the least active compounds, **4s** (**C**) and **4b** (**D**), on the TG2 protein.

The therapeutic target studied was transglutaminase 2 (TG2), in accordance with Gundemir et al. [52], who related TG2 overexpression to cell survival and proliferation in glioblastomas (GMBs). For this study, the Protein Data Bank structure 3S3J [53] was used to identify the predominant interactions contributing to the biological activity of the most active compounds, particularly **4q** and **4v**, and to compare them with the less active compounds **4b** and **4s** (Figure 4).

The arrangement of the most active ligands revealed that the chalcone fragment oriented the *para*-halogenated phenyl group in the hydrophobic region, constituted by Leu420 and Phe316 in the case of compound **4q**. On the other hand, compound **4v** penetrated deeper into this pocket, establishing additional interactions with Tyr315, Tyr575, and Leu312, in addition to Phe316. This enhanced binding is attributed to the *ortho*-methoxy substitution on the benzyl group in **4v**, which allowed the formation of a hydrogen bond with Asn333, facilitating its entry into a more hydrophobic region compared to **4q**.

In contrast, the less active compounds **4b** and **4s** failed to establish similar hydrophobic interactions, due to the absence of hydrogen bond acceptor groups at the *ortho*-position of the benzyl group (Figure 4D). Additional substitutions, such as the dichlorinated phenyls in compound **4t**, prevented the ligand from adopting a suitable orientation to interact with Phe316 and Leu420.

#### 2.2.3. Therapeutic Target Involved in Gb-d1—MEK

MEK is an active kinase implicated in several gallbladder cancer cell lines [54]. The protein crystallized structure, obtained from the Protein Data Bank (PDB ID: 7M0T) [55], contains the ligand selumetinib, a kinase inhibitor whose binding site is located in the innermost region of the kinase, thereby inhibiting the active state of MEK.

**Figure 5:**
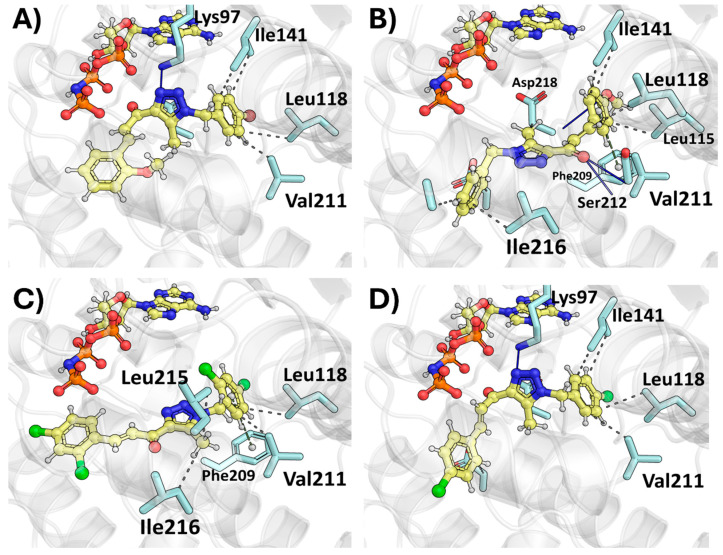
A representation of the best pose achieved for the most active compounds, **4k** (**A**) and **4o** (**B**), and the least active compounds, **4s** (**C**) and **4b** (**D**), on MEK protein.

The most active ligands, **4k** and **4o**, showed favorable interactions similar to those of selumetinib (SI). In the case of compound **4k**, hydrogen bond interactions with Lys97 were favored, resulting in additional hydrophobic interactions with Ile141, Leu118, and Val211. Compound **4o** primarily formed a hydrogen bond between the carbonyl group and amide of Ser212, enhancing its interaction with the hydrophobic region constituted by Ile141, Leu118, Leu115, and Phe209 (Figure 5).

In contrast, the less active compounds **4b** and **4s** exhibited poor interactions with the hydrophobic region. Although compound **4b** possesses a single substituent on the aromatic ring, which could favor an interaction similar to that of **4q**, the absence of a substituent at the *ortho*-position and the presence of chlorine at the *para*-position prevented the ligand from stabilizing in the innermost hydrophobic region of the binding site. For compound **4s**, the disubstitution of the aromatic rings hindered the formation of a hydrogen bond at the binding site.

#### 2.2.4. Therapeutic Target Involved in HeLa—Dihydrofolate Reductase, DHFR

**Figure 6:**
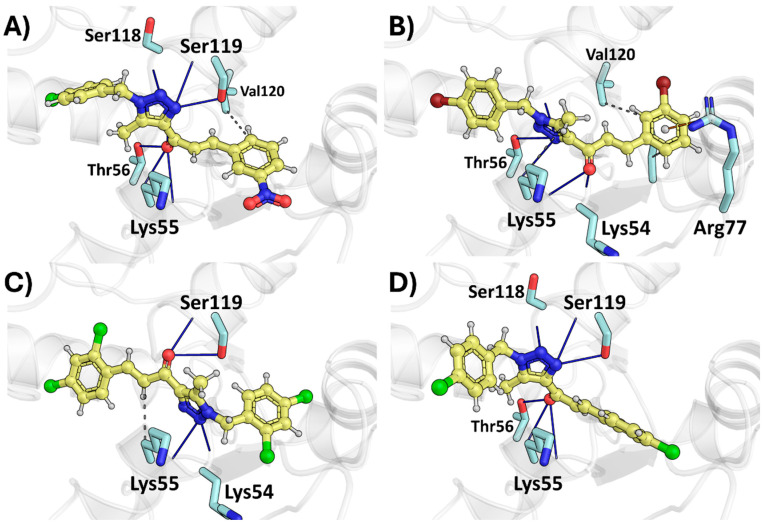
A representation of the best pose achieved for the most active compounds, **4g** (**A**) and **4f** (**B**), and the least active compounds, **4s** (**C**) and **4b** (**D**), in the DHFR protein.

According to our previously published work, one of the most significant therapeutic targets in HeLa cells is dihydrofolate reductase (DHFR) [56]. The docking poses obtained corresponded to compounds **4g** and **4f**, which exhibited activities of 42.5 and 44.5 μM, respectively. Similarly to the analysis in Section 2.2.2, these ligands were compared with the less active compounds **4b** and **4s** (Figure 6).

All four ligands formed hydrogen bonds with the binding site through their carbonyl and triazole groups. The most notable differences arose from the substitutions on the aromatic rings. In the case of compound **4g**, the triazole arrangement allowed hydrogen bonding with Ser118 and Ser119, while the carbonyl interacted with Thr56. The aromatic rings enhanced these interactions, providing orientations that stabilized the carbonyl group, such as the arrangement of the *m*-nitrophenyl ring towards Val120. In the case of compound **4f**, its orientation included an additional interaction with Arg77, suggesting that substitutions at the *meta*-position favored the positioning of the ring in the hydrophobic region.

In contrast, ligands **4b** and **4s** lacked these substitutions, and instead featured *para*-substituents, which hindered the optimal arrangement of the carbonyl group. As a result, in compound **4b**, the rings were oriented towards the solvent-exposed region, whereas in compound **4s**, the rings adopted an alternative conformation to facilitate hydrogen bonding between the carbonyl and Ser119, thereby losing crucial hydrophobic interactions.

### 2.3. Cytotoxic Activities

The cytotoxic effects of chalcones **4a**–**4v** were evaluated against five cancer cell lines (G415, Gbd1, T98G, HeLa, and U937) using the MTT colorimetric assay after 24 h of exposure (Table 4) [57]. The IC_50_ values were determined from dose–response curves, and ranged from 4.4 to >200 μM, indicating a variable cytotoxic effect, depending on both the structure of the chalcones and the specific cell line tested. Compounds with IC_50_ values greater than 200 μM were considered inactive. 

Among the tested cell lines, the human non-Hodgkin lymphoma cell line (U937) was the most sensitive, with 16 chalcones exhibiting IC_50_ values below 20 μM. Notably, compounds **4a**, **4c**, **4d**, **4e**, **4f**, **4g**, **4h**, **4i**, **4j**, **4k**, **4l**, **4m**, **4n**, **4o**, **4t**, and **4v** showed IC_50_ values between 4.4 and 17.8 μM. The most potent compound against U937 was **4c** (IC_50_ = 4.4 μM), followed by **4a** (IC_50_ = 4.6 μM), both featuring substitutions at the *para* position. However, they differ in the nature of their substituents. Compound **4a** has a bromine atom, whereas compound **4c** contains a chlorine atom and a methoxy group at the *para* position of the aromatic ring of the chalcone. The human glioblastoma cell line (T98G) was sensitive to **4d**, **4f**, **4i**, **4j**, **4k**, **4l**, **4m**, **4o**, **4p**, **4q**, **4r**, and **4v**, with IC_50_ values ranging from 11 to 66.5 μM. The most active compounds were **4v** (IC_50_ = 11 μM), **4r** (IC_50_ = 14 μM), and **4q** (IC_50_ = 12.3 μM), all characterized by the presence of bulky groups at R_1_ and R_2_, as well as halogen groups at the R_3_ or R_5_ positions. Compound **4v** was the most active, likely due to the presence of substituents at the *ortho* position. This structural feature is absent in the less active compounds **4g** and **4i**, suggesting the importance of substituent position in modulating biological activity in this cell line. The human gallbladder carcinoma lines (Gb-d1) exhibited moderate sensitivity to 10 chalcones, with IC_50_ values ranging from 16 to 58.8 μM, comparable to the reference drug (paclitaxel, IC_50_ = 21 μM). The most potent compound was **4o** (IC_50_ = 16 μM), followed by **4h** (IC_50_ = 17.2 μM), both characterized by the presence of chlorine atoms at the R_3_ and R_5_ positions. The human cervical adenocarcinoma cell line (HeLa) was slightly sensitive to only four chalcones (**4f**, **4g**, **4m**, and **4o**), with IC_50_ values ranging from 42.5 to 95.5 μM, indicating lower sensitivity compared to other cell lines. The main difference between these compounds and the others is the absence of substitutions at the *para* position of the aromatic rings. Compound **4g** features chlorine substitutions at the *ortho* position and a nitro group at the *ortho* position of the chalcone fragment. A similar trend was observed with gallbladder carcinoma cell line (G-415), which was the least sensitive. Only three compounds (**4f**, **4g**, and **4m**) showed weak cytotoxicity, with IC_50_ values of 27.6, 51, and 26 μM, respectively.

Interestingly, chalcones with halogen substituents (Cl, Br) exhibited enhanced cytotoxic activity in multiple cell lines, particularly U937 and T98G, suggesting a possible influence of electronic effects on their mechanism of action. On the other hand, chalcones containing electron-donating groups, such as a methoxy group, showed moderate activity, especially in T98G and HeLa cell lines. When comparing the activity of chalcones with that of paclitaxel, a well-known anticancer agent, it is notable that compounds such as **4a**, **4c**, and **4d** in U937, and **4v** in T98G, exhibited IC_50_ values within a comparable range, suggesting their potential as lead compounds for further optimization. However, the presence of multiple chlorine atoms in compounds such as **4b** and **4s** was associated with reduced efficacy, negatively affecting their biological activity (**4b** and **4s**). Overall, the cytotoxic activity of the chalcones appears to be influenced by both structural modifications and the specific cell line tested. Further investigations will be necessary to fully evaluate their therapeutic potential.

## 3. Materials and Methods

### 3.1. General Section

Melting points were determined on a Stuart SMP3 apparatus (Staffordshire UK), and are uncorrected. IR spectra were recorded on a Perkin-Elmer FT-IR Spectrometer Spectrum Two (Llantrisant, UK) with KBr. NMR spectra were acquired in DMSO-*d*_6_ or CDCl_3_ with a Varian Unity Inova 500 MHz spectrometer (Palo Alto, CA, USA). Chemical shifts are reported in parts per million (δ) relative to the residual solvent signals (DMSO-d_6_: δ_H_ 2.50, δ_C_ 39.5 or CDCl_3_; δ_H_ H 7.26, δ_C_ C 77.2) as internal standards for the ^1^H and ^13^C NMR spectra. Coupling constants (*J*) are expressed in Hz. HRMS spectra were recorded using a Micromass-LCT Premier Time-of-Flight ESI spectrometer, coupled with an ACQUITY UHPLC (Milford, Massachusetts, USA) (ultra-high-performance liquid chromatography) interface system. For copies of ^1^H, ^13^C NMR, HSQC, HMB, and HSMS-ESI, see Section 4 in the Supplementary Materials. The reactions were monitored by thin-layer chromatography (TLC), performed on silica gel Merck 60 F_254_. The components were visualized under UV light (254 and 365 nm), and/or by treatment with phosphomolybdic acid reagent, followed by heating. All starting materials and reagents were obtained from commercial suppliers [46,47,58,59,60,61].

### 3.2. General Procedure for Synthesis of Triazolic Chalcones (**4a**–**4v**) (Exemplified for Synthesis of **4a**)


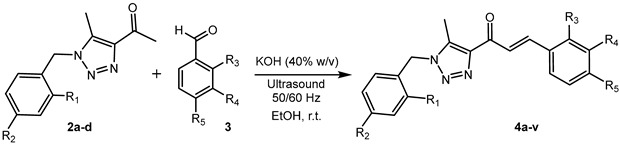


A mixture of 1.0 g (4.0 mmol) of **2a**, 0.74 g (4.0 mmol) of 4-bromobenzaldehyde, and 40 mL of ethanol was subjected to ultrasound (50/60 Hz) for 2 min. Then, 7.0 mL of KOH (40% *w*/*v*) was added over 2 min. After completing the addition, the reaction mixture was subjected to ultrasound (50/60 Hz) at room temperature for 10 min. The resulting precipitate was filtered, washed with ethanol, and vacuum-dried, yielding 1.52 g (90% yield) of **4a** as a white solid (Rf: 0.72, eluent: hexane/EtOAc, 1/1).

*(E)-3-(4-bromophenyl)-1-(1-(4-chlorobenzyl)-5-methyl-1H-1,2,3-triazol-4-yl)-2-propen-1-one* (**4a**).

White solid (90% yield), m.p.: 184-186 °C. Rf: 0.72, eluent: hexane/EtOAc, 1/1. IR (cm^−1^) v: 3063 and 3026 (CAr-H); 2988 and 2967 (Csp3-H); 1684 (C=O); 1601 (C=C); 1592 (CAr-CAr); 1554 (N=N). ^1^H-NMR (CDCl_3_, 500 MHz) δ (ppm): 2.55 (3H, s), 5.52 (2H, s), 7.14 (2H, d, *J =* 8.3 Hz, H4), 7.34 (2H, d, *J =* 8.4 Hz), 7.55 (4H, m), 7.80 (1H, d, *J =* 16.0 Hz), 8.02 (1H, d, *J =* 16.0 Hz). ^13^C-NMR (CDCl_3_, 125.7 MHz) δ (ppm): 9.5 (CH_3_), 51.2 (CH2), 123.6 (CH), 125.0 (C), 128.8 (2CH), 129.8 (2CH), 130.2 (2CH), 132.3 (2CH), 132.5 (C), 134.0 (C), 134.9 (C), 138.1 (C), 142.4 (CH), 144.4 (C), 184.2 (C). HRMS-ESI (*m*/*z*) calculated for C_19_H_16_C_l_BrN_3_O [M + H]^+^: 416.01652, found: 416.01669.

*(E)-3-(4-chlorophenyl)-1-(1-(4-chlorobenzyl)-5-methyl-1H-1,2,3-triazol-4-yl)-2-propen-1-one* (**4b**).

White solid (85% yield), m.p.: 176–178 °C. Rf: 0.77, eluent: hexane/EtOAc, 1/1. IR (cm^−1^) v: 3053 (CAr-H); 2984 and 2952 (Csp3-H); 1661 (C=O); 1601 (C=C); 1590 (CAr-CAr); 1567 (N=N). ^1^H-NMR (CDCl_3_, 500 MHz) δ (ppm): 2.55 (3H, s), 5.51 (2H, s), 7.14 (2H, d, *J =* 8.3 Hz), 7.34 (2H, d, *J =* 8.4 Hz), 7.38 (2H, d, *J =* 8.4 Hz), 7.63 (2H, d, *J =* 8.4 Hz), 7.82 (1H, d, *J =* 16.0 Hz), 8.00 (1H, d, *J =* 16.0 Hz). ^13^C-NMR (CDCl_3_, 125.7 MHz) δ (ppm): 9.5 (CH_3_), 51.2 (CH_2_), 123.5 (CH), 128.8 (2CH), 129.3 (2CH), 129.6 (2CH), 130.0 (2CH), 132.6 (C), 133.6 (C), 134.9 (C), 136.6 (C), 138.1 (C), 142.3 (CH), 144.4 (C), 184.2 (C). HRMS-ESI (*m*/*z*) calculated for C_19_H_16_Cl_2_N_3_O [M + H]^+^: 372.06703, found: 372.06772.

*(E)-1-((4-chlorobenzyl)-5-methyl-1H-1,2,3-triazol-4-yl)-3-(4-methoxyphenyl)-2-propen-1-one* (**4c**).

Yellow solid (91% yield), m.p.: 173–175 °C. Rf: 0.63, eluent: hexane/EtOAc, 1/1. IR (cm^−1^) v: 3084 nd 3051 (CAr-H); 2981; 2948 and 2849 (Csp3-H); 1656 (C=O); 1591 (C=C); 1570 (CAr-CAr); 1570 (N=N). ^1^H-NMR (CDCl_3_, 500 MHz) δ (ppm): 2.52 (3H, s), 3.82 (3H, s), 5.48 (2H, s), 6.91 (2H, d, *J =* 8.3 Hz), 7.11 (2H, d, *J =* 8.1 Hz), 7.30 (2H, d, *J =* 7.9 Hz), 7.63 (2H, d, *J =* 8.3 Hz), 7.82 (1H, d, *J =* 15.9 Hz), 7.90 (1H, d, *J =* 15.8 Hz). ^13^C-NMR (CDCl_3_, 125.7 MHz) δ (ppm): 9.4 (CH_3_), 51.0 (CH_2_), 55.4 (CH_3_), 114.4 (2CH), 120.6 (CH), 127.7 (C), 128.7 (2CH), 129.4 (2CH), 130.5 (2CH), 132.6 (C), 134.6 (C), 137.7 (C), 143.5 (CH), 144.4 (C), 161.8 (C), 184.3 (C). HRMS-ESI (*m*/*z*) calculated for C_20_H_19_ClN_3_O_2_ [M + H]^+^: 368.11657, found: 368.11703.

*(E)-3-(2,4-dichlorophenyl)-1-(1-(4-chlorobenzyl)-5-methyl-1H-1,2,3-triazol-4-yl)-2-propen-1-ona* (**4d**).

White solid (90% yield), m.p.: 181–182 °C. Rf: 0.75, eluent: hexane/EtOAc, 1/1. IR (cm^−1^) v: 3064 and 3025 (CAr-H); 2990 and 2952 (Csp3-H); 1669 (C=O); 1604 (C=C); 1583 (CAr-CAr); 1561(N=N). ^1^H-NMR (CDCl_3_, 500 MHz) δ (ppm): 2.56 (3H, s,), 5.52 (2H, s), 7.14 (2H, d, *J =* 8.3 Hz), 7.30 (1H, d, *J =* 8.5 Hz), 7.34 (2H, d, *J =* 8.4 Hz), 7.46 (1H, s), 7.81 (1H, d, *J =* 8.5 Hz), 8.00 (1H, d, *J =* 16.0 Hz), 8.22 (1H, d, *J =* 16.0 Hz). ^13^C-NMR (CDCl_3_, 125.7 MHz) δ (ppm): 9.5 (CH_3_), 51.2 (CH_2_), 125.6 (CH), 127.7 (CH), 128.8 (3CH), 129.6 (2CH), 130.2 (CH), 132.9 (C), 132.5 (C), 134.7 (C), 136.4 (C), 136.7 (CH), 138.2 (C), 138.3 (CH), 144.3 (C), 183.8 (C). HRMS-ESI (*m*/*z*) calculated for C_19_H_15_N_3_OCl_3_ [M + H]^+^: 406.02806, found: 406.02866.

*(E)-3-(2-chlorophenyl)-1-(1-(4-chlorobenzyl)-5-methyl-1H-1,2,3-triazol-4-yl)-2-propen-1-one* (**4e**).

White solid (75% yield), m.p.: 144–146 °C. Rf: 0.69, eluent: hexane/EtOAc, 1/1. IR (cm^−1^) v: 3067 (CAr-H); 2992 and 2956 (Csp3-H); 1667 (C=O); 1604 (C=C); 1583 (CAr-CAr); 1561 (N=N). ^1^H-NMR (CDCl_3_, 500 MHz) δ (ppm): 2.56 (3H, s), 5.52 (2H, s), 7.14 (2H, d, *J =* 8.3 Hz), 7.31 (2H, dd, *J =* 8.8 and 5.5 Hz), 7.34 (2H, d, *J =* 8.4 Hz), 7.43 (1H, d, *J =* 6.8 Hz), 7.88 (1H, d, *J =* 6.7 Hz), 8.03 (1H, d, *J =* 15.9 Hz), 8.31 (1H, d, *J =* 15.9 Hz). ^13^C-NMR (CDCl_3_, 125.7 MHz) δ (ppm): 9.5 (CH_3_), 51.2 (CH_2_), 125.1 (CH), 127.2 (C), 128.2 (CH), 128.8 (2CH), 129.6 (2CH), 130.3 (CH), 131.4 (CH), 132.6 (C), 133.3 (C), 134.9 (C), 135.9 (CH), 138.2 (C), 139.5 (CH), 144.4 (C), 184.1 (C). HRMS-ESI (*m*/*z*) calculated for C_19_H_16_Cl_2_N_3_O [M + H]^+^: 372.06703, found: 372.06665.

*(E)-1-((4-bromobenzyl)-5-methyl-1H-1,2,3-triazol-4-yl)-3-(3-bromophenyl)-2-propen-1-one* (**4f**).

White solid (82% yield), m.p.: 141–143 °C. Rf: 0.75, eluent: hexane/EtOAc, 1/1. IR (cm^−1^) *v*: 3067 and 3026 (CAr-H); 2990 and 2952 (Csp3-H); 1667 (C=O); 1611 (C=C); 1585 (CAr-CAr); 1560 (N=N). ^1^H-NMR (CDCl_3_, 500 MHz) δ (ppm): 2.55 (3H, s), 5.51 (2H, s), 7.08 (2H, d, *J =* 8.2 Hz), 7.85 (1H, s), 7.29 (1H, t, *J =* 7.8 Hz), 7.50 (2H, d, *J =* 8.3 Hz), 7.61 (1H, d, *J =* 8.5 Hz), 8.02 (1H, d, *J =* 15.9 Hz), 7.78 (1H, d, *J =* 16.0 Hz). ^13^C-NMR (CDCl_3_, 125.7 MHz) δ (ppm): 9.0 (CH_3_), 50.9 (CH_2_), 122.9 (C), 124.2 (CH), 127.3 (CH), 129.0 (2CH), 130.4 (CH), 131.3 (CH), 132.4 (2CH), 132.5 (C), 132.9 (CH), 136.5 (C), 138.1 (C), 141.9 (CH), 144.3 (C), 183.9 (C). HRMS-ESI (*m*/*z*) calculated for C_19_H_16_Br_2_N_3_O [M + H]^+^: 459.96600, found: 459.96606.

*(E)-1-((4-chlorobenzyl)-5-methyl-1H-1,2,3-triazol-4-yl)-3-(3-nitrophenyl)-2-propen-1-one* (**4g**).

Orange solid (60% yield), m.p.: 182–183 °C. Rf: 0.81, eluent: hexane/EtOAc, 1/1. IR (cm^−1^) v: 3090 (CAr-H); 3061 (CAr-H); 2964 (Csp3-H); 2949 (Csp3-H); 2917 (Csp3-H); 1661 (C=O); 1600 (C=C); 1571 (CAr-CAr); 1561 (N=N); 1528 (N=O); 1345 (N-O). ^1^H-NMR (CDCl_3_, 500 MHz) δ (ppm): 2.57 (3H, s), 5.53 (2H, s), 7.15 (2H, d, *J =* 8.2 Hz), 8.53 (1H, s), 7.61 (1H, t, *J =* 7.9 Hz), 7.35 (2H, d, *J =* 8.3 Hz), 8.25 (1H, d, *J =* 8.5 Hz), 8.13 (1H, d, *J =* 15.9 Hz), 7.89 (1H, d, *J =* 16.0 Hz). ^13^C-NMR (CDCl_3_, 125.7 MHz) δ (ppm): 9.0 (CH_3_), 50.9 (CH_2_), 134.7 (C), 123.1 (CH), 124.6 (C), 125.6 (CH), 128.0 (C), 128.8 (2CH), 129.4 (CH), 129.5 (2CH), 132.0 (C), 134.1 (CH), 137.9 (C), 140.6 (CH), 144.0 (C), 148.6 (C), 183.4 (C). HRMS-ESI (*m*/*z*) calculated for C_19_H_16_ClN_4_O_3_ [M + H]^+^: 383.09108, found: 383.09238.

*(E)-1-((4-bromobenzyl)-5-methyl-1H-1,2,3-triazol-4-yl)-3-(2-chlorophenyl)-2-propen-1-one* (**4h**).

White solid (85% yield), m.p.: 167–168 °C. Rf: 0.77, eluent: hexane/EtOAc, 1/1. IR (cm^−1^) v: 3061 (CAr-H); 2990 and 2955 (Csp3-H); 1667 (C=O); 1603 (C=C); 1593 (CAr-CAr); 1555 (N=N). ^1^H-NMR (CDCl_3_, 500 MHz) δ (ppm): 2.56 (3H, s), 5.50 (2H, s), 7.08 (2H, d, *J =* 8.3 Hz), 7.31 (1H, dd, *J =* 6.8 and 5.3 Hz), 7.33 (1H, d, *J =* 5.7 and 3.5 Hz), 7.43 (1H, d, *J =* 6.8 Hz), 7.50 (2H, d, *J =* 8.4 Hz), 7.88 (1H, d, *J =* 7.1 Hz), 8.03 (1H, d, *J =* 16.0 Hz), 8.31 (1H, d, *J =* 16.0 Hz). ^13^C-NMR (CDCl_3_, 125.7 MHz) δ (ppm): 9.5 (CH_3_), 51.2 (CH_2_), 123.0 (C), 125.2 (CH), 127.2 (C), 128.1 (CH), 129.1 (2CH), 130.3 (CH), 131.4 (CH), 132.5 (2CH), 130.1 (C), 133.3 (C), 135.9 (CH), 138.2 (C), 139.5 (CH), 144.4 (C), 184.1 (C). HRMS-ESI (*m*/*z*) calculated for C_19_H_16_BrClN_3_O [M + H]^+^: 416.01652, found: 416.01660.

*(E)-1-((1-(4-bromobenzyl)-5-methyl-1H-1,2,3-triazol-4-yl)-3-(2,4-dichlorophenyl)-2-propen-1-one* (**4i**).

White solid (71% yield), m.p.: 183–185 °C. Rf: 0.78, eluent: hexane/EtOAc, 1/1. IR (cm^−1^) v: 3063 and 3022 (CAr-H); 2991 and 2953 (Csp3-H); 1667 (C=O); 1603 (C=C); 1582 (CAr-CAr); 1559 (N=N). ^1^H-NMR (CDCl_3_, 500 MHz) δ (ppm): 2.56 (3H, s), 5.50 (2H, s), 7.08 (2H, d, *J =* 8.2 Hz), 7.30 (1H, d, *J =* 8.4 Hz), 7.46 (1H, s), 7.50 (2H, d, *J =* 8.3 Hz), 7.81 (1H, d, *J =* 8.5 Hz), 8.00 (1H, d, *J =* 15.9 Hz), 8.22 (1H, d, *J =* 16.0 Hz). ^13^C-NMR (CDCl_3_, 125.7 MHz) δ (ppm): 9.5 (CH_3_), 51.3 (CH_2_), 123.0 (C), 125.6 (CH), 127.7 (CH), 128.8 (CH), 129.1 (2CH), 130.2 (C), 131.9 (CH), 132.6 (2CH), 133.0 (C), 136.5 (C), 136.7 (CH), 138.2 (C), 138.3 (CH), 144.3 (C), 183.8 (C). HRMS-ESI (*m*/*z*) calculated for C_19_H_15_BrCl_2_N_3_O [M + H]^+^: 449.97754, found: 449.97662.

*(E)-1-((4-bromobenzyl)-5-methyl-1H-1,2,3-triazol-4-yl)-3-(4-methoxyphenyl)-2-propen-1-one* (**4j**).

Yellow solid (56% yield), m.p.: 185–187 °C. Rf: 0.67, eluent: hexane/EtOAc, 1/1. IR (cm^−1^) v: 3049 (CAr-H); 2950 (Csp3-H); 1658 (C=O); 1592 (C=C); 1572 (CAr-CAr); 1552 (N=N). ^1^H-NMR (CDCl_3_, 500 MHz) δ (ppm): 2.55 (3H, s), 3.85 (3H, s), 5.49 (2H, s), 6.93 (2H, d, *J =* 8.2 Hz), 7.07 (2H, d, *J =* 8.0 Hz), 7.49 (2H, d, *J =* 8.0 Hz), 7.66 (2H, d, *J =* 8.3 Hz), 7.85 (1H, d, *J =* 15.9 Hz), 7.92 (1H, d, *J =* 15.9 Hz). ^13^C-NMR (CDCl_3_, 125.7 MHz) δ (ppm): 9.5 (CH_3_), 51.2 (CH_2_), 55.6 (CH_3_), 114.4 (2CH), 120.6 (CH), 122.8 (C), 127.7 (C), 128.9 (2CH), 130.6 (2CH), 132.4 (2CH), 133.1 (C), 137.7 (C), 143.6 (CH), 144.5 (C), 161.9 (C), 184.5 (C). HRMS-ESI (*m*/*z*) calculated for C_20_H_19_BrN_3_O_2_ [M + H]^+^: 412.06605, found: 412.06699.

*(E)-1-((4-bromobenzyl)-5-methyl-1H-1,2,3-triazol-4-yl)-3-(2-methoxyphenyl)-2-propen-1-one* (**4k**).

Yellow solid (85% yield), m.p.: 130–132 °C. Rf: 0.67, eluent: hexane/EtOAc, 1/1. IR (cm^−1^) v: 3058 (CAr-H); 2964 (Csp3-H); 2949 (Csp3-H); 2843 (Csp3-H); 1657 (C=O); 1589 (C=C); 1563 (CAr-CAr); 1552 (N=N). ^1^H-NMR (CDCl_3_, 500 MHz) δ (ppm): 2.55 (3H, s), 3.91 (3H, s), 5.49 (2H, s), 6.92 (1H, d, *J =* 8.1 Hz), 6.98 (1H, dd, *J =* 7.1 and 14.6 Hz), 7.07 (2H, d, *J =* 8.2 Hz), 7.36 (1H, dd, *J =* 7.8 and 7.5 Hz), 7.48 (2H, d, *J =* 8.2 Hz), 7.75 (1H, d, *J =* 7.6 Hz), 8.07 (1H, d, *J =* 16.1 Hz), 8.26 (1H, d, *J =* 16.1 Hz). ^13^C-NMR (CDCl_3_, 125.7 MHz) δ (ppm): 9.4 (CH_3_), 51.1 (CH_2_), 55.7 (CH_3_), 111.3 (CH), 120.8 (C), 122.9 (C), 123.2 (CH), 124.0 (CH), 128.4 (C), 129.0 (2CH), 132.0 (CH), 132.4 (2CH), 133.2 (C), 137.9 (C), 139.1 (CH), 144.7 (C), 159.0 (C), 184.8 (C). HRMS-ESI (*m*/*z*) calculated for C_20_H_19_BrN_3_O_2_ [M + H]^+^: 412.06605, found: 412.06635.

*(E)-1-((4-bromobenzyl)-5-methyl-1H-1,2,3-triazol-4-yl)-3-(3,4-dimethoxyphenyl)-2-propen-1-one* (**4l**).

Yellow solid (79% yield), m.p.: 154–156 °C. Rf: 0.47, eluent: hexane/EtOAc, 1/1. IR (cm^−1^) v: 3051 (CAr-H); 2953 (Csp3-H); 2836 (Csp3-H); 1653 (C=O); 1593 (CAr-CAr); 1568 (N=N). ^1^H-NMR (CDCl_3_, 500 MHz) δ (ppm): 2.56 (3H, s), 3.94 (3H, s), 3.97 (3H, s), 5.50 (2H, s), 6.85 (1H, d, *J =* 8.2 Hz), 7.08 (2H, d, *J =* 8.1 Hz), 7.26 (2H, d, *J =* 8.2 Hz), 7.50 (2H, d, *J =* 8.2 Hz), 7.85 (1H, d, *J =* 15.9 Hz), 7.91 (1H, d, *J =* 15.9 Hz). ^13^C-NMR (CDCl_3_, 125.7 MHz) δ (ppm): 9.5 (CH_3_), 51.2 (CH_2_), 56.1 (2CH_3_), 110.0 (CH), 111.2 (CH), 120.8 (CH), 122.9 (C), 124.1 (CH), 128.1 (C), 129.1 (2CH), 132.5 (2CH), 133.2 (C), 137.9 (C), 144.1 (CH), 144.6 (C), 149.4 (C), 151.7 (C), 184.3 (C). HRMS-ESI (*m*/*z*) calculated for C_21_H_21_BrN_3_O_3_ [M + H]^+^: 442.07662, found: 442.07532.

*(E)-1-(benzyl)-5-methyl-1H-1,2,3-triazol-4-yl)-3-phenyl-2-propen-1-one* (**4m**).

White solid (84% yield), m.p.: 153–155 °C (Lit. 157–158 °C [62]). Rf: 0.68, eluent: hexane/EtOAc, 1/1. IR (cm^−1^) v: 3060 (CAr-H); 3032 (CAr-H); 2973 (Csp3-H); 1663 (C=O); 1600 (C=C); 1574 (CAr-CAr); 1561 (CAr-CAr). ^1^H-NMR (CDCl_3_, 500 MHz) δ (ppm): 2.54 (3H, s), 5.54 (2H, s), 7.18 (2H, d, *J =* 7.1 Hz), 7.32 (3H, m), 7.39 (3H, m), 7.88 (1H, d, *J =* 16.0 Hz), 8.06 (1H, d, *J =* 16.0 Hz). ^13^C-NMR (CDCl_3_, 125.7 MHz) δ (ppm): 9.4 (CH_3_), 51.8 (CH_2_), 123.0 (CH), 127.3 (2CH), 128.7 (CH), 128.8 (2CH), 128.9 (2CH), 129.2 (2CH), 130.6 (CH), 134.1 (C), 135.5 (C), 138.1 (C), 143.6 (CH), 144.4 (C), 184.4 (C). HRMS-ESI (*m*/*z*) calculated for C_19_H_18_N_3_O [M + H]^+^: 304.14498, found: 304.14496.

*(E)-1-(benzyl)-5-methyl-1H-1,2,3-triazol-4-yl)-3-(4-chlorophenyl)-2-propen-1-one* (**4n**).

White solid (90% yield), m.p.: 160–161 °C (Lit. 158–159 °C [62]). Rf: 0.76, eluent: hexane/EtOAc, 1/1. IR (cm^−1^) v: 3057 (CAr-H); 3031 (CAr-H); 2969 (Csp3-H); 1662 (C=O); 1603 (C=C); 1590 (CAr-CAr); 1566 (N=N). ^1^H-NMR (CDCl_3_, 500 MHz) δ (ppm): 2.55 (3H, s), 5.56 (2H, s), 7.19 (2H, d, *J =* 8.3 Hz), 7.35 (3H, m), 7.39 (2H, d, *J =* 8.4 Hz), 7.63 (2H, d, *J =* 8.4 Hz), 7.82 (1H, d, *J =* 16.0 Hz), 8.02 (1H, d, *J =* 16.0). ^13^C-NMR (CDCl_3_, 125.7 MHz) δ (ppm): 9.0 (CH_3_), 51.6 (CH_2_), 123.5 (CH), 127.2 (2CH), 127.5 (CH), 129.2 (2CH), 129.2 (2CH), 129.9 (2CH), 133.4 (C), 133.8 (C), 136.6 (C), 138.0 (C), 142.1 (CH), 144.0 (C), 184.0 (C). HRMS-ESI (*m*/*z*) calculated for C_19_H_17_ClN_3_O [M + H]^+^: 338.10600, found: 338.10522.

*(E)-1-(bencil)-5-metil-1H-1,2,3-triazol-4-il)-3-(2,4-diclorofenil)-2-propen-1-ona* (**4o**).

White solid (98% yield), m.p.: 145–147 °C (Lit. 142–143 °C [62]). Rf: 0.63, eluent: hexane/EtOAc, 1/1. IR (cm^−1^) v: 3087 (CAr-H); 3062 (CAr-H); 3033 (CAr-H); 2949 (Csp3-H); 1667 (C=O); 1604 (C=C); 1580 (CAr-CAr); 1559 (N=N). ^1^H-NMR (CDCl_3_, 500 MHz) δ (ppm): 2.56 (3H, s), 5.56 (2H, s), 7.19 (2H, d, *J =* 8.1 Hz), 7.30 (1H, dd, *J =* 8.5 and 2.1 Hz), 7.35 (3H, m), 7.46 (1H, d, *J =* 2.1 Hz), 7.82 (1H, d, *J =* 8.5 Hz), 8.02 (1H, d, *J =* 15.9 Hz), 8.22 (1H, d, *J =* 15.9 Hz). ^13^C-NMR (CDCl_3_, 125.7 MHz) δ (ppm): 9.0 (CH_3_), 51.6 (CH_2_), 123.9 (C), 125.5 (CH), 127.3 (2CH), 127.6 (CH), 128.8 (CH), 129.0 (2CH), 130.0 (CH), 133.7 (C), 136.4 (C), 136.7 (C), 137.9 (C), 138.0 (CH), 144.2 (C), 183.6 (C). HRMS-ESI (*m*/*z*) calculated for C_19_H_16_Cl_2_N_3_O [M + H]^+^: 372.06703, found: 372.06754.

*(E)-1-(benzyl)-5-methyl-1H-1,2,3-triazol-4-yl)-3-(4-methoxyphenyl)-2-propen-1-one* (**4p**).

Yellow solid (50% yield), m.p.: 133–135 °C (Lit. 132–133 °C [62]). Rf: 0.69, eluent: hexane/EtOAc, 1/1. IR (cm^−1^) v: 3069 and 3035 (CAr-H); 2979, 2947 and 2834 (Csp3-H); 1657 (C=O); 1598 (C=C); 1574 (CAr-CAr), 1568 (N=N). ^1^H-NMR (CDCl_3_, 500 MHz) δ (ppm): 2.55 (3H, s), 5.55 (2H, s), 7.19 (2H, d, *J =* 8.3 Hz), 7.34 (3H, m), 6.94 (2H, d, *J =* 8.2 Hz), 7.66 (2H, d, *J =* 8.3 Hz), 7.93 (1H, d, *J =* 15.9 Hz), 7.85 (1H, d, *J =* 15.9 Hz). ^13^C-NMR (CDCl_3_, 125.7 MHz) δ (ppm): 9.0 (CH_3_), 51.5 (CH_2_), 56.8 (CH_3_), 114.3 (2CH), 120.6 (CH), 127.1 (CH), 127.2 (2CH), 127.7 (C), 129.0 (2CH), 130.5 (2CH), 133.8 (C), 137.6 (C), 143.5 (CH), 144.7 (C), 161.9 (C), 184.4 (C). HRMS-ESI (*m*/*z*) calculated for C_20_H_20_N_3_O_2_ [M + H]^+^: 334.15554, found: 334.15646.

*(E)-1-(2,4-dichlorobenzyl)-5-methyl-1H-1,2,3-triazol-4-yl)-3-(4-chlorophenyl)-2-propen-1-one* (**4q**).

White solid (95% yield), m.p.: 190–192 °C. Rf: 0.82, eluent: hexane/EtOAc, 1/1. IR (cm^−1^) v: 3081 (CAr-H); 2956 (Csp3-H); 1666 (C=O); 1608 (C=C); 1589 (CAr-CAr); 1568 (N=N). ^1^H-NMR (CDCl_3_, 500 MHz) δ (ppm): 2.59 (3H, s), 5.62 (2H, s), 6.81 (1H, d, *J =* 8.3 Hz), 7.22 (1H, d, *J =* 8.4 Hz), 7.47 (1H, s), 7.39 (2H, d, *J =* 8.4 Hz), 7.64 (2H, d, *J =* 8.4 Hz), 7.84 (1H, d, *J =* 16.0 Hz), 8.01 (1H, d, *J =* 16.0 Hz). ^13^C-NMR (CDCl_3_, 125.7 MHz) δ (ppm): 8.8 (CH_3_), 47.9 (CH_2_), 123.3 (CH), 127.6 (2CH), 128.0 (CH), 129.5 (CH), 129.7 (CH), 129.9 (2CH), 130.2 (C), 133.6 (C), 133.7 (C), 136.6 (C), 136.9 (C), 137.9 (C), 142.4 (CH), 144.1 (C), 183.9 (C). HRMS-ESI (*m*/*z*) calculated for C_19_H_15_Cl_3_N_3_O [M + H]^+^: 406.02806, found: 406.02927.

*(E)-1-(2,4-dichlorobenzyl)-5-methyl-1H-1,2,3-triazol-4-yl)-3-(2-chlorophenyl)-2-propen-1-one* (**4r**).

White solid (86% yield), m.p.: 158–161 °C. Rf: 0.79, eluent: hexane/EtOAc, 1/1. IR (cm^−1^) v: 3080 (CAr-H); 3031 (CAr-H); 2957 (Csp3-H); 1666 (C=O); 1605 (C=C); 1588 (CAr-CAr); 1563 (N=N). ^1^H-NMR (CDCl_3_, 500 MHz) δ (ppm): 2.60 (3H, s), 5.63 (2H, s), 6.81 (1H, d, *J =* 8.3 Hz), 7.22 (1H, d, *J =* 8.4 Hz), 7.48 (1H, s), 7.33 (2H, m), 7.44 (1H, d, *J =* 6.8 Hz), 7.88 (1H, d, *J =* 6.7 Hz), 8.04 (1H, d, *J =* 15.9 Hz), 8.33 (1H, d, *J =* 15.9 Hz). ^13^C-NMR (CDCl_3_, 125.7 MHz) δ (ppm): 8.9 (CH_3_), 47.9 (CH_2_), 125.1 (CH), 127.2 (CH), 128.0 (CH), 128.1 (CH), 129.5 (CH), 129.8 (CH), 130.2 (C), 130.3 (CH), 131.4 (CH), 133.3 (C), 133.7 (C), 135.6 (C), 135.9 (C), 138.2 (C), 139.5 (CH), 144.0 (C), 183.7 (C). HRMS-ESI (*m*/*z*) calculated for C_19_H_15_Cl_3_N_3_O [M + H]^+^: 406.02806, found: 406.02872.

*(E)-1-(2,4-dichlorobenzyl)-5-methyl-1H-1,2,3-triazol-4-yl)-3-(2,4-dichlorophenyl)-2-propen-1-one* (**4s**).

White solid (98% yield), m.p.: 201–203 °C. Rf: 0.83, eluent: hexane/EtOAc, 1/1. IR (cm^−1^) v: 3090 (CAr-H); 3064 (CAr-H); 2953 (Csp3-H); 1669 (C=O); 1605 (C=C); 1584 (CAr-CAr); 1559 (N=N). ^1^H-NMR (CDCl_3_, 500 MHz) δ (ppm): 2.60 (3H, s), 5.62 (2H, s), 6.81 (1H, d, *J =* 8.3 Hz), 7.22 (1H, d, *J =* 8.4 Hz), 7.46 (1H, d, *J =* 5.6 Hz), 7.31 (1H, d, *J =* 8.4 Hz), 7.46 (1H, d, *J =* 5.6 Hz), 7.81 (1H, d, *J =* 8.5 Hz), 8.02 (1H, d, *J =* 15.9 Hz), 8.24 (1H, d, *J =* 16.0 Hz). ^13^C-NMR (CDCl_3_, 125.7 MHz) δ (ppm): 9.0 (CH_3_), 48.2 (CH_2_), 125.6 (CH), 127.6 (CH), 128.0 (CH), 128.7 (CH), 129.6 (CH), 129.8 (CH), 130.0 (CH), 130.1 (C), 132.9 (C), 133.8 (C), 135.6 (C), 136.3 (C), 136.7 (C), 138.3 (CH), 138.4 (C), 144.3 (C), 183.6 (C). HRMS-ESI (*m*/*z*) calculated for C_19_H_14_Cl_4_N_3_O [M + H]^+^: 439.98909, found: 439.98917.

*(E)-1-(2,4-dichlorobenzyl)-5-methyl-1H-1,2,3-triazol-4-yl)-3-(3,4-dimethoxyphenyl)-2-propen-1-one* (**4t**).

Yellow solid (80% yield), m.p.: 185–187 °C. Rf: 0.62, eluent: hexane/EtOAc, 1/1. IR (cm^−1^) v: 3064 (CAr-H); 3008 (CAr-H); 2965 (Csp3-H); 2836 (Csp3-H); 1656 (C=O); 1595 (C=C); 1583 (CAr-CAr); 1562 (N=N). ^1^H-NMR (CDCl_3_, 500 MHz) δ (ppm): 2.59 (3H, s), 3.94 (3H, s), 3.96 (3H, s), 5.62 (2H, s), 6.79 (1H, d, *J =* 8.3 Hz), 6.90 (1H, d, *J =* 8.4 Hz), 7.26 (2H, d, *J =* 8.3 Hz), 7.22 (1H, d, *J =* 8.4 Hz), 7.47 (1H, s), 7.87 (1H, d, *J =* 16.0 Hz), 7.92 (1H, d, *J =* 16.0 Hz). ^13^C-NMR (CDCl_3_, 125.7 MHz) δ (ppm): 8.8 (CH_3_), 47.9 (CH_2_), 56.1 (2CH_3_), 110.0 (CH), 111.2 (CH), 120.5 (CH), 124.1 (CH), 128.0 (CH), 128.1 (C), 129.5 (CH), 129.7 (CH), 130.2 (C), 133.7 (C), 135.7 (C), 138.0 (C), 144.1 (CH), 144.2 (C), 149.4 (C), 151.7 (C), 184.2 (C). HRMS-ESI (*m*/*z*) calculated for C_21_H_20_Cl_2_N_3_O_3_ [M + H]^+^: 432.08816, found: 432.08911.

*(E)-1-(2-methoxybenzyl)-5-methyl-1H-1,2,3-triazol-4-yl)-3-(4-bromophenyl)-2-propen-1-one* (**4v**).

White solid (80% yield), m.p.: 193–195 °C. Rf: 0.19, eluent: hexane/EtOAc, 1/1. IR (cm^−1^) v: 3051 (CAr-H); 3016 (CAr-H); 2966 (Csp3-H); 2943 (Csp3-H); 2839 (Csp3-H); 1661 (C=O); 1601 (C=C); 1586 (CAr-CAr); 1564 (N=N). ^1^H-NMR (CDCl_3_, 500 MHz) δ (ppm): 2.59 (3H, s); 3.88 (3H, s); 5.55 (2H, s); 6.92 (3H, m); 7.31 (1H, t, *J =* 7.6 Hz); 7.55 (4H, m); 7.80 (1H, d, *J =* 16.0 Hz); 8.04 (1H, d, *J =* 16.0 Hz). ^13^C-NMR (CDCl_3_, 125.70 MHz) δ (ppm): 8.79 (CH_3_); 46.00 (CH_2_); 55.21 (CH_3_); 110.46 (CH); 120.98 (CH); 123.74 (CH); 124.96 (C); 128.56 (C); 128.78 (CH); 129.83 (C); 129.91 (CH); 130.22 (2CH); 132.29 (2CH); 133.98 (C); 138.44 (C); 141.98 (CH); 143.70 (C); 184.08 (C). HRMS-ESI (*m*/*z*) calculated for C_20_H_19_BrN_3_O_2_ [M + H]^+^: 412.06605, found: 412.06635.

### 3.3. Computational Method

In this study, four target proteins were selected: VEGFR (PDB ID 4ASD) [51], TG2 (PDB ID 3S3J) [53], MEK (PDB ID 7M0T) [55], and DHFR (PDB ID 4M6J) [63]. These structures were obtained from the Protein Data Bank (PDB) database. The crystallographic structures were cleaned and prepared using the Schrödinger Protein Preparation Wizard module, which includes the correct assignment of protonation states, removal of non-essential water molecules, and optimization of protein geometry and energetics.

The selected ligands were prepared using the LigPrep module of Schrödinger [64], which includes the generation of possible ionization states and tautomers, geometry optimization, and energy minimization. In addition, proper ligand conformations were ensured to guarantee accurate and realistic interactions during the docking process.

Once the proteins and ligands were prepared, molecular docking was performed using Glide with the Standard Precision (SP) and Extra Precision (XP) modules [65,66]. This software enabled the evaluation of the interactions between the ligands and the active sites of the proteins, providing docking scores indicating the affinity and stability of the formed complexes. This approach provided insight into the essential residues of the co-crystallized ligands and the synthesized ligands responding to high affinity towards VEGFR, TG2, MEK, and DHFR, thus contributing to their correlation with biological activity.

### 3.4. Cytotoxixity Assay

Cell culture: The cell lines G-415 (Human gallbladder adenocarcinoma) Gbd1 (Human gallbladder adenocarcinoma), U-937 (human histiocytic lymphoma), HeLa (human cervix adenocarcinoma), and T98G (human brain glioblastoma multiforme) were purchased from the American Type Culture Collection-ATCC (Manassas, VA, USA). In our laboratory, these cells are routinely cultured in media optimized for maximum proliferation. All cell lines were grown at 37 °C in a humidified atmosphere of 5% CO_2_/95% air, and the adherent cells were removed from culture plates by trypsinization (0.53 mM EDTA, 0.05% trypsin). G-415 and Gbd1 were maintained in RPMI 1640, 2 mM glutamine, 10% FBS, and penicillin/streptomycin/amphotericin-B (100 units/mL; 100 µg/mL; 0.25 µg/mL). Confluent cultures of these two adherent cell lines were split 1:3 to 1:6 by trypsinization and seeding at 2–4 × 10^4^ cells/cm^2^. U937 cells were maintained in RPMI 1640, 2 mM glutamine, 10% FBS, and penicillin/streptomycin/amphotericin-B (100 units/mL; 100 µg/mL; 0.25 µg/mL). Cultures were maintained at 2–9 × 10^5^ cells/mL. Three times a week, the culture cells were diluted under the same conditions to maintain optimal density, and were harvested during the exponential growth phase. HeLa cells were cultured in MEM, 2mM glutamine, 10% FBS, 1% non-essential amino acids, and penicillin/streptomycin/amphotericin-B (100 units/mL; 100 µg/mL; 0.25 µg/mL). T98G cells were maintained in DMEM-F12, 2 mM glutamine, 10% FBS, 1% non-essential amino acids, 1% sodium pyruvate, and penicillin/streptomycin/amphotericin-B (100 units/mL; 100 µg/mL; 0.25 µg/mL). Confluent cultures of these two adherent cell lines were split 1:3 to 1:6 by trypsinization and seeded at 2–4 × 10^4^ cells/cm^2^.

Measurement of cytotoxicity by MTT assay: Aliquots of 200 μL of cell suspension (5 × 10^5^/mL) were seeded into 96-well polystyrene tissue culture plates. Then, 2.0 μL of different extract dilutions and vehicle control were added in triplicate to each well. Cells were seeded in 96-well plates (100 μL/well) and, after 24 h, cells were treated with a medium containing the compounds at concentrations ranging from 0 up to 200 μM, for an additional 24 h. The compounds were dissolved in DMSO (1% final concentration) and complete medium. Untreated cells (medium containing 1% DMSO) were used as 100% viability controls. Paclitaxel (98% purity, Sigma-Aldrich, St. Louis, MO, USA) was used as the reference compound. Each concentration was tested in triplicate, and experiments were repeated twice. Cell viability was determined using the MTT reduction assay at the end of the incubation period with the compounds. The results were expressed as a percentage of the control, and IC_50_ values were graphically determined from the dose–response curves.

Cells incubated in culture medium alone served as the control for cell viability (untreated cells). Cells were placed in a humidified 5% CO_2_ incubator at 37 °C for 24 h. After incubation, 10 μL aliquots of MTT solution (5 mg/mL in PBS) were added to each well and re-incubated for 4 h at 37 °C, followed by low-speed centrifugation at 800 rpm for 5 min. Then, 200 μL of the supernatant culture medium were carefully aspirated, and 200 μL aliquots of DMSO were added to each well to dissolve the formazan crystals. The plates were then incubated for 10 min to dissolve air bubbles. The culture plate was placed on an E*max* model micro-plate reader (Molecular Devices, San Jose, CA, USA), and the absorbance was measured using a 650 nm filter. The amount of color produced is directly proportional to the number of viable cells. All assays were performed in duplicate, with three replicates each, and processed independently. Means ± SD values were used to estimate cell viability. The cell viability rate was calculated as the percentage of MTT absorption, as follows:

% survival = (mean experimental absorbance/mean control absorbance) × 100.

The extract concentration was plotted against the corresponding percentage (%) of cell viability obtained with MTT assays, and the 50% inhibitory concentration (IC_50_) was calculated by non-linear regression. Curve fitting was performed using GraphPad Prism^®^6 from Systat Software, Inc. (Richmond, CA, USA) (Figures S1–S21 in Supplementary Materials). 

Statistical analysis: Data were analyzed by one-way analysis of variance, and Student’s *t*-test was used to determine statistical significance (GraphPad Prism^®^6). Each experiment was performed in triplicate on two independent occasions. The results are expressed as mean ± SD. Differences with *p* values of < 0.05 were considered statistically significant.

## 4. Conclusions 

In this study, a series of 1,2,3-triazole-linked chalcones were synthesized and characterized using an ultrasound-assisted strategy, yielding compounds with potential antiproliferative activity. Spectroscopic characterization confirmed the structures of the compounds, and their biological activity was evaluated in various tumor cell lines.

Molecular docking studies allowed the identification of key interactions between the most active compounds and their respective therapeutic targets. In the U937 line, compounds **4a** and **4c** exhibited strong affinity for the VEGFR protein, establishing hydrogen bonds with critical residues such as Cys919 and Asp1046, along with hydrophobic interactions involving Val848 and Phe918. However, the T98G protein showed a positive response to the affinity of the most active ligands (**4q** and **4v**), which interacted with key residues Phe316 and Leu420. In contrast, the less active compounds (**4b** and **4s**) possessed substitutions that failed to adopt the correct position to facilitate these interactions. For the Gb-d1 line, derivatives **4k** and **4o** showed favorable interactions with MEK, forming hydrogen bonds with Lys97 and Ser212, respectively, along with hydrophobic interactions involving Ile141 and Leu118. In the HeLa cell line, compounds **4g** and **4f** demonstrated a high affinity for the DHFR enzyme, forming hydrogen bonds with Ser118 and Ser119, and achieving additional stabilization through interactions with Val120 and Arg77.

Cytotoxicity assays confirmed the moderate antiproliferative activity of several compounds, with IC_50_ values ranging from 4.4 to 32.2 µM, particularly in three of the five cell lines studied: U937, non-Hodgkin lymphoma; T98G, glioblastoma multiforme tumor; and Gb-d1, gallbladder cancer cells. A correlation between biological activity and the orientation and type of substituents on the chalcone and triazole structures was observed, suggesting that these structural modifications could modulate affinity for target proteins and, therefore, cytotoxic activity.

In conclusion, this study demonstrates that chalcones linked to 1,2,3-triazoles represent an interesting scaffold for the development of novel cytotoxic compounds. The combination of docking studies and biological assays has enabled the establishment of a structure–activity relationship that can be exploited in future research to optimize the efficacy of these compounds. In vivo assays and additional mechanistic studies will be essential to validate their therapeutic potential.

## Figures and Tables

**Table 1:** Yields (%) obtained in the final step of the synthesis of substituted triazole chalcones, illustrating the influence of different substituents on the overall reaction yield.

Comp.	R_1_	R_2_	R_3_	R_4_	R_5_	Yield (%)
**4a**	H	Cl	H	H	Br	90
**4b**	H	Cl	H	H	Cl	85
**4c**	H	Cl	H	H	OMe	91
**4d**	H	Cl	Cl	H	Cl	90
**4e**	H	Cl	Cl	H	H	75
**4f**	H	Br	H	Br	H	82
**4g**	H	Cl	H	NO_2_	H	60
**4h**	H	Br	Cl	H	H	85
**4i**	H	Br	Cl	H	Cl	71
**4j**	H	Br	H	H	OMe	56
**4k**	H	Br	OMe	H	H	85
**4l**	H	Br	H	OMe	OMe	79
**4m**	H	H	H	H	H	84
**4n**	H	H	H	H	Cl	90
**4o**	H	H	Cl	H	Cl	98
**4p**	H	H	H	H	OMe	50
**4q**	Cl	Cl	H	H	Cl	95
**4r**	Cl	Cl	Cl	H	H	86
**4s**	Cl	Cl	Cl	H	Cl	98
**4t**	Cl	Cl	H	OMe	OMe	80
**4v**	OMe	H	H	H	Br	80

**Table 2:** Characteristic signals of vinyl protons of *trans*-chalcones in ^1^H-NMR.

						^1^H-NMR (δ/ppm)			^13^C-NMR (δ/ppm)	IR (cm^−1^)
Comp.	R_1_	R_2_	R_3_	R_4_	R_5_	H_α_	H_β_	*J* (Hz)	C=O	C=O
**4a**	H	Cl	H	H	Br	7.80	8.02	16.0	184.2	1684
**4b**	H	Cl	H	H	Cl	7.82	8.00	16.0	184.2	1661
**4c**	H	Cl	H	H	OMe	7.82	7.90	15.9	184.3	1656
**4d**	H	Cl	Cl	H	Cl	8.00	8.22	16.0	183.8	1669
**4e**	H	Cl	Cl	H	H	8.03	8.31	15.9	184.1	1667
**4f**	H	Br	H	Br	H	7.78	8.02	16.0	183.9	1667
**4g**	H	Cl	H	NO_2_	H	7.89	8.13	16.0	183.4	1661
**4h**	H	Br	Cl	H	H	8.03	8.31	16.0	184.1	1667
**4i**	H	Br	Cl	H	Cl	8.00	8.22	16.0	183.8	1667
**4j**	H	Br	H	H	OMe	7.85	7.92	15.9	184.5	1658
**4k**	H	Br	OMe	H	H	8.07	8.26	16.1	184.8	1657
**4l**	H	Br	H	OMe	OMe	7.85	7.91	15.9	184.3	1653
**4m**	H	H	H	H	H	7.88	8.06	16.0	184.4	1663
**4n**	H	H	H	H	Cl	7.82	8.02	16.0	184.0	1662
**4o**	H	H	Cl	H	Cl	7.82	8.02	15.9	183.6	1667
**4p**	H	H	H	H	OMe	7.85	7.93	15.9	184.4	1657
**4q**	Cl	Cl	H	H	Cl	7.84	8.01	16.0	183.9	1666
**4r**	Cl	Cl	Cl	H	H	8.04	8.33	15.9	183.7	1666
**4s**	Cl	Cl	Cl	H	Cl	8.02	8.24	16.0	183.6	1669
**4t**	Cl	Cl	H	OMe	OMe	7.87	7.92	16.0	184.2	1656
**4v**	OMe	H	H	H	Br	7.80	7.04	16.0	184.1	1661

**Table 3:** The binding energy between the most active and least active ligands. The activity results are expressed in IC_50_ µM, and Score corresponds to the affinity energy obtained with Glide XP, and is expressed in kcal/mol.

VEGFR (U937)			TG2 (T98G)			MEK (Gb-d1)			DHFR (HeLa)		
Comp	Activity	Score	Comp	Activity	Score	Comp	Activity	Score	Comp	Activity	Score
**4a**	4.60	−8.54	**4q**	12.30	−7.56	**4k**	20.00	−8.55	**4h**	42.50	−4.84
**4c**	4.40	−8.00	**4v**	11.00	−8.35	**4o**	16.00	−8.84	**4f**	44.50	−5.59
**4b**	200.00	−6.32	**4b**	200.00	−4.33	**4b**	200.00	−7.24	**4b**	200.00	−4.10
**4s**	200.00	−6.14	**4s**	200.00	−4.02	**4s**	200.00	−7.14	**4s**	200.00	−4.45

**Table 4:** The cytotoxic activity of derivative compounds **4a** to **4v** against the selected tumor cell lines.

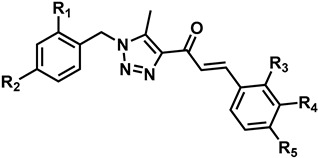										
						IC_50_ (µM)				
Comp.	R_1_	R_2_	R_3_	R_4_	R_5_	U937	T98G	Gb-d1	Hela	G-415
**4a**	H	Cl	H	H	Br	4.6 ± 0.28	>200	>200	>200	>200
**4b**	H	Cl	H	H	Cl	>200	>200	>200	>200	>200
**4c**	H	Cl	H	H	OMe	4.4 ± 0.2	>200	>200	>200	>200
**4d**	H	Cl	Cl	H	Cl	10.8 ± 0.6	24.6 ± 0.1	>200	>200	>200
**4e**	H	Cl	Cl	H	H	12.3 ± 0.3	>200	>200	>200	>200
**4f**	H	Br	H	Br	H	11	21.8 ± 0.3	22.3 ± 1.8	42.5 ± 0.7	27.6 ± 0.2
**4g**	H	Cl	H	NO_2_	H	9.6 ± 0.14	16	24	44.5 ± 2.1	51
**4h**	H	Br	Cl	H	H	11.3 ± 0.3	22	17.2 ± 1.2	>200	>200
**4i**	H	Br	Cl	H	Cl	17.5	20.3 ± 1.1	58.8 ± 2.5	>200	>200
**4j**	H	Br	H	H	OMe	9.5 ± 0.7	66.5 ± 1.6	39.5 ± 0.3	>200	>200
**4k**	H	Br	OMe	H	H	17.8 ± 0.4	44.3 ± 1.8	20	>200	>200
**4l**	H	Br	H	OMe	OMe	12.3 ± 0.3	43.1	23 ± 2.8	>200	>200
**4m**	H	H	H	H	H	8	52 ± 0.7	32.2 ± 1.1	86	26 ± 4.2
**4n**	H	H	H	H	Cl	11	45.6	>200	>200	>200
**4o**	H	H	Cl	H	Cl	10	29	16 ± 3.5	95.5 ± 0.7	>200
**4p**	H	H	H	H	OMe	83 ± 1.4	>200	>200	>200	>200
**4q**	Cl	Cl	H	H	Cl	>200	12.3 ± 0.3	>200	>200	>200
**4r**	Cl	Cl	Cl	H	H	>200	14	>200	>200	>200
**4s**	Cl	Cl	Cl	H	Cl	>200	>200	>200	>200	>200
**4t**	Cl	Cl	H	OMe	OMe	16	>200	20 ± 0.7	>200	>200
**4v**	OMe	H	H	H	Br	8.2 ± 0.3	11 ± 0.9	>200	>200	>200
Paclitaxel						6	21	41	6.2	10

## Data Availability

Data are contained within the article and the Supplementary Materials.

## Supplementary Materials

The following supporting information can be downloaded at https://www.mdpi.com/article/10.3390/ijms26073389/s1: [46,47,58,59,60,61,62].

## References

[1] Khan S.A., Akhtar M.J., Gogoi U., Meenakshi D.U., Das A. (2023). An Overview of 1,2,3-Triazole-Containing Hybrids and Their Potential Anticholinesterase Activities. Pharmaceuticals.

[2] Lengerli D., Ibis K., Nural Y., Banoglu E. (2022). The 1,2,3-Triazole All-in-One Ring System in Drug Discovery: A Good Bioisostere, a Good Pharmacophore, a Good Linker, and a Versatile Synthetic Tool. Expert. Opin. Drug. Discov..

[3] Matin M.M., Matin P., Rahman M.R., Ben Hadda T., Almalki F.A., Mahmud S., Ghoneim M.M., Alruwaily M., Alshehri S. (2022). Triazoles and Their Derivatives: Chemistry, Synthesis, and Therapeutic Applications. Front. Mol. Biosci..

[4] Bozorov K., Zhao J., Aisa H.A. (2019). 1,2,3-Triazole-Containing Hybrids as Leads in Medicinal Chemistry: A Recent Overview. Bioorg. Med. Chem..

[5] Cunha A.C., Figueiredo J.M., Tributino J.L.M., Miranda A.L.P., Castro H.C., Zingali R.B., Fraga C.A.M., De Souza M.C.B.V., Ferreira V.F., Barreiro E.J. (2003). Antiplatelet Properties of Novel *N*-Substituted-Phenyl-1,2,3-Triazole-4-Acylhydrazone Derivatives. Bioorg. Med. Chem..

[6] Jordão A.K., Ferreira V.F., Lima E.S., de Souza M.C.B.V., Carlos E.C.L., Castro H.C., Geraldo R.B., Rodrigues C.R., Almeida M.C.B., Cunha A.C. (2009). Synthesis, Antiplatelet and in Silico Evaluations of Novel *N*-Substituted-Phenylamino-5-Methyl-1*H*-1,2,3-Triazole-4-Carbohydrazides. Bioorg. Med. Chem..

[7] Cogan D.A., Aungst R., Breinlinger E.C., Fadra T., Goldberg D.R., Hao M.H., Kroe R., Moss N., Pargellis C., Qian K.C. (2008). Structure-Based Design and Subsequent Optimization of 2-tolyl-(1,2,3-Triazol-1-yl-4-carboxamide) Inhibitors of P38 MAP Kinase. Bioorg. Med. Chem. Lett..

[8] Chen P.C., Patil V., Guerrant W., Green P., Oyelere A.K. (2008). Synthesis and Structure-Activity Relationship of Histone Deacetylase (HDAC) Inhibitors with Triazole-Linked Cap Group. Bioorg. Med. Chem..

[9] Bhat B.A., Reddy P.B., Agrawal S.K., Saxena A.K., Kumar H.M.S., Qazi G.N. (2008). Studies on Novel 4β-[(4-substituted)-1,2,3-Triazol-1-yl] Podophyllotoxins as Potential Anticancer Agents. Eur. J. Med. Chem..

[10] Kelley J.L., Koble C.S., Davis R.G., McLean E.W., Soroko F.E., Barrett R. (1995). Cooper 1-(fluorobenzyl)-4-Amino-1H-1,2,3-Triazolo [4,5-c]Pyridines: Synthesis and Anticonvulsant Activity. J. Med. Chem..

[11] Mahmood S., Khan S.G., Rasul A., Christensen J.B., Abourehab M.A.S. (2022). Ultrasound Assisted Synthesis and In Silico Modelling of 1,2,4-Triazole Coupled Acetamide Derivatives of 2-(4-Isobutyl Phenyl)Propanoic Acid as Potential Anticancer Agents. Molecules.

[12] Calderone V., Fiamingo F.L., Amato G., Giorgi I., Livi O., Martelli A., Martinotti E. (2008). 1,2,3-Triazol-Carboxanilides and 1,2,3-Triazol-(*N*-Benzyl)-Carboxamides as BK-Potassium Channel Activators. XII. Eur. J. Med. Chem..

[13] Holla B.S., Mahalinga M., Karthikeyan M.S., Poojary B., Akberali P.M., Kumari N.S. (2005). Synthesis, Characterization and Antimicrobial Activity of Some Substituted 1,2,3-Triazoles. Eur. J. Med. Chem..

[14] Zhang T.Y., Li C.S., Cao L.T., Bai X.Q., Zhao D.H., Sun S.M. (2022). New Ursolic Acid Derivatives Bearing 1,2,3-Triazole Moieties: Design, Synthesis and Anti-Inflammatory Activity in Vitro and in Vivo. Mol. Divers..

[15] Kuntala N., Mareddy J., Telu J.R., Banothu V., Pal S., Anireddy J.S. (2021). Nonsteroidal Anti-Inflammatory Drugs Based New 1,2,3-Triazole Derivatives: Their Design, One-Pot Synthesis and in Vitro Evaluation. J. Heterocycl. Chem..

[16] Alvarez R., Velázquez S., San-félix A., Aquaro S., De Clercq E., Pemo C., Karlsson A., Balzarini J., Camarasa M.J. (1994). 1,2,3-Triazole-[2,5-Bis-O-(Tert-Butyldimethylsilyl)-Beta.-D-Ribofuranosyl]-3′-Spiro-5″-(4″-Amino-1″,2″-Oxathiole 2″,2″-Dioxide) (TSAO) Analogs: Synthesis and Anti-HIV-1 Activity. J. Med. Chem..

[17] Silva F.d.C.d., de Souza M.C.B.V., Frugulhetti I.I.P., Castro H.C., Souza S.L.d.O., de Souza T.M.L., Rodrigues D.Q., Souza A.M.T., Abreu P.A., Passamani F. (2009). Synthesis, HIV-RT Inhibitory Activity and SAR of 1-Benzyl-1*H*-1,2,3-Triazole Derivatives of Carbohydrates. Eur. J. Med. Chem..

[18] Jordão A.K., Afonso P.P., Ferreira V.F., de Souza M.C.B.V., Almeida M.C.B., Beltrame C.O., Paiva D.P., Wardell S.M.S.V., Wardell J.L., Tiekink E.R.T. (2009). Antiviral Evaluation of *N*-Amino-1,2,3-Triazoles against Cantagalo Virus Replication in Cell Culture. Eur. J. Med. Chem..

[19] da Silva E.N., Menna-Barreto R.F.S., Pinto M.d.C.F.R., Silva R.S.F., Teixeira D.V., de Souza M.C.B.V., De Simone C.A., De Castro S.L., Ferreira V.F., Pinto A.V. (2008). Naphthoquinoidal [1,2,3]-Triazole, a New Structural Moiety Active against Trypanosoma Cruzi. Eur. J. Med. Chem..

[20] da Silva Júnior E.N., de Souza M.C.B.V., Fernandes M.C., Menna-Barreto R.F.S., Pinto M.d.C.F.R., de Assis Lopes F., de Simone C.A., Andrade C.K.Z., Pinto A.V., Ferreira V.F. (2008). Synthesis and Anti-Trypanosoma Cruzi Activity of Derivatives from nor-Lapachones and Lapachones. Bioorg. Med. Chem..

[21] Gill C., Jadhav G., Shaikh M., Kale R., Ghawalkar A., Nagargoje D., Shiradkar M. (2008). Clubbed [1,2,3] Triazoles by Fluorine Benzimidazole: A Novel Approach to H37Rv Inhibitors as a Potential Treatment for Tuberculosis. Bioorg. Med. Chem. Lett..

[22] Upadhayaya R.S., Kulkarni G.M., Vasireddy N.R., Vandavasi J.K., Dixit S.S., Sharma V., Chattopadhyaya J. (2009). Design, Synthesis and Biological Evaluation of Novel Triazole, Urea and Thiourea Derivatives of Quinoline against Mycobacterium Tuberculosis. Bioorg. Med. Chem..

[23] Wilkinson B.L., Bornaghi L.F., Wright A.D., Houston T.A., Poulsen S.A. (2007). Anti-Mycobacterial Activity of a Bis-Sulfonamide. Bioorg. Med. Chem. Lett..

[24] Castagnolo D., Radi M., Dessì F., Manetti F., Saddi M., Meleddu R., De Logu A., Botta M. (2009). Synthesis and Biological Evaluation of New Enantiomerically Pure Azole Derivatives as Inhibitors of Mycobacterium Tuberculosis. Bioorg. Med. Chem. Lett..

[25] Dabak K., Sezer Ö., Akar A., Anaç O. (2003). Synthesis and Investigation of Tuberculosis Inhibition Activities of Some 1,2,3-Triazole Derivatives. Eur. J. Med. Chem..

[26] Costa M.S., Boechat N., Rangel É.A., da Silva F.d.C., de Souza A.M.T., Rodrigues C.R., Castro H.C., Junior I.N., Lourenço M.C.S., Wardell S.M.S.V. (2006). Synthesis, Tuberculosis Inhibitory Activity, and SAR Study of N-Substituted-Phenyl-1,2,3-Triazole Derivatives. Bioorg. Med. Chem..

[27] Ferreira S.B., Costa M.S., Boechat N., Bezerra R.J.S., Genestra M.S., Canto-Cavalheiro M.M., Kover W.B., Ferreira V.F. (2007). Synthesis and Evaluation of New Difluoromethyl Azoles as Antileishmanial Agents. Eur. J. Med. Chem..

[28] Beena, Kumar N., Rohilla R.K., Roy N., Rawat D.S. (2009). Synthesis and Antibacterial Activity Evaluation of Metronidazole-Triazole Conjugates. Bioorg. Med. Chem. Lett..

[29] Poonia N., Kumar A., Kumar V., Yadav M., Lal K. (2021). Recent Progress in 1*H*-1,2,3-Triazoles as Potential Antifungal Agents. Curr. Top Med. Chem..

[30] Kong Y., Wang K., Edler M.C., Hamel E., Mooberry S.L., Paige M.A., Brown M.L. (2010). A Boronic Acid Chalcone Analog of Combretastatin A-4 as a Potent Anti-Proliferation Agent. Bioorg. Med. Chem..

[31] Dhaliwal J.S., Moshawih S., Goh K.W., Loy M.J., Hossain S., Hermansyah A., Kotra V., Kifli N., Goh H.P., Kaur S. (2022). Pharmacotherapeutics Applications and Chemistry of Chalcone Derivatives. Molecules.

[32] Zhuang C., Zhang W., Sheng C., Zhang W., Xing C., Miao Z. (2017). Chalcone: A Privileged Structure in Medicinal Chemistry. Chem. Rev..

[33] Mezgebe K., Melaku Y., Mulugeta E. (2023). Synthesis and Pharmacological Activities of Chalcone and Its Derivatives Bearing *N*-Heterocyclic Scaffolds: A Review. ACS Omega.

[34] Mohammed H.H.H., Abd El-Hafeez A.A., Ebeid K., Mekkawy A.I., Abourehab M.A.S., Wafa E.I., Alhaj-Suliman S.O., Salem A.K., Ghosh P., Abuo-Rahma G.E.D.A. (2022). New 1,2,3-Triazole Linked Ciprofloxacin-Chalcones Induce DNA Damage by Inhibiting Human Topoisomerase I& II and Tubulin Polymerization. J. Enzyme Inhib. Med. Chem..

[35] Zhang S.Y., Fu D.J., Yue X.X., Liu Y.C., Song J., Sun H.H., Liu H.M., Zhang Y.B. (2016). Design, Synthesis and Structure-Activity Relationships of Novel Chalcone-1,2,3-Triazole-Azole Derivates as Antiproliferative Agents. Molecules.

[36] Kınalı M., Çol S., Çoban C.Ç., Türk M., Aydın G., Emirik M., Baran A. (2023). Chalcone-Based Dipolar Cycloaddition of Novel Heteroaromatic Compounds: Their Anticancer Examination. J. Mol. Struct..

[37] Yadav P., Lal K., Kumar A., Guru S.K., Jaglan S., Bhushan S. (2017). Green Synthesis and Anticancer Potential of Chalcone Linked-1,2,3-Triazoles. Eur. J. Med. Chem..

[38] Zhao L., Mao L., Hong G., Yang X., Liu T. (2015). Design, Synthesis and Anticancer Activity of Matrine-1*H*-1,2,3-Triazole-Chalcone Conjugates. Bioorg. Med. Chem. Lett..

[39] Constantinescu T., Lungu C.N. (2021). Anticancer Activity of Natural and Synthetic Chalcones. Int. J. Mol. Sci..

[40] Ouyang Y., Li J., Chen X., Fu X., Sun S., Wu Q. (2021). Chalcone Derivatives: Role in Anticancer Therapy. Biomolecules.

[41] Yadav M., Lal K., Kumar A., Kumar A., Kumar D. (2022). Indole-Chalcone Linked 1,2,3-Triazole Hybrids: Facile Synthesis, Antimicrobial Evaluation and Docking Studies as Potential Antimicrobial Agents. J. Mol. Struct..

[42] Singh G., Arora A., Kalra P., Maurya I.K., Ruizc C.E., Estebanc M.A., Sinha S., Goyal K., Sehgal R. (2019). A Strategic Approach to the Synthesis of Ferrocene Appended Chalcone Linked Triazole Allied Organosilatranes: Antibacterial, Antifungal, Antiparasitic and Antioxidant Studies. Bioorg. Med. Chem..

[43] Mahapatra D.K., Bharti S.K., Asati V. (2015). Chalcone Scaffolds as Anti-Infective Agents: Structural and Molecular Target Perspectives. Eur. J. Med. Chem..

[44] Singh P., Anand A., Kumar V. (2014). Recent Developments in Biological Activities of Chalcones: A Mini Review. Eur. J. Med. Chem..

[45] Sooknual P., Pingaew R., Phopin K., Ruankham W., Prachayasittikul S., Ruchirawat S., Prachayasittikul V. (2020). Synthesis and Neuroprotective Effects of Novel Chalcone-Triazole Hybrids. Bioorg. Chem..

[46] Chen X.B., Shi D.Q. (2008). Synthesis and Biological Activity of Novel Phosphonate Derivatives Containing of Pyridyl and 1,2,3-Triazole Rings. Phosphorus Sulfur Silicon Relat. Elem..

[47] Nelson R., Kesternich V., Pérez-Fehrmann M., Jaldin S., Marcourt L., Christen P. (2016). Regiospecific Synthesis of 1,4,5-Trisubstituted 1,2,3-Triazoles via Enolate-Azide Cycloaddition between 1,3-Dicarbonyl Compounds and Aryl Azides. J. Chem. Res..

[48] Jarag K.J., Pinjari D.V., Pandit A.B., Shankarling G.S. (2011). Synthesis of Chalcone (3-(4-Fluorophenyl)-1-(4-Methoxyphenyl)Prop-2-En-1-One): Advantage of Sonochemical Method over Conventional Method. Ultrason. Sonochem..

[49] Haghi A., Salami M., Kian M.M., Nikbakht M., Mohammadi S., Chahardouli B., Rostami S., Malekzadeh K. (2020). Effects of Sorafenib and Arsenic Trioxide on U937 and KG-1 Cell Lines: Apoptosis or Autophagy?. Cell J..

[50] Inaba H., Rubnitz J.E., Coustan-Smith E., Li L., Furmanski B.D., Mascara G.P., Heym K.M., Christensen R., Onciu M., Shurtleff S.A. (2011). Phase I Pharmacokinetic and Pharmacodynamic Study of the Multikinase Inhibitor Sorafenib in Combination with Clofarabine and Cytarabine in Pediatric Relapsed/Refractory Leukemia. J. Clin. Oncol..

[51] McTigue M., Murray B.W., Chen J.H., Deng Y.L., Solowiej J., Kania R.S. (2012). Molecular Conformations, Interactions, and Properties Associated with Drug Efficiency and Clinical Performance among VEGFR TK Inhibitors. Proc. Natl. Acad. Sci. USA.

[52] Gundemir S., Monteagudo A., Akbar A., Keillor J.W., Johnson G.V.W. (2017). The Complex Role of Transglutaminase 2 in Glioblastoma Proliferation. Neuro. Oncol..

[53] Lindemann I., Boettcher J., Oertel K., Weber J., Hils M., Pasternack R., Heine A., Klebe G. (2011). Inhibitors of Transglutaminase 2: A Therapeutic Option in Celiac Disease. RCSB Protein Data Bank.

[54] Canale M., Monti M., Rapposelli I.G., Ulivi P., Sullo F.G., Bartolini G., Tiberi E., Frassineti G.L. (2021). Gallbladder Cancer. Cancers.

[55] Gonzalez-Del Pino G.L., Li K., Park E., Schmoker A.M., Ha B.H., Eck M.J. (2021). Allosteric MEK Inhibitors Act on BRAF/MEK Complexes to Block MEK Activation. Proc. Natl. Acad. Sci. USA.

[56] Pérez-Fehrmann M., Kesternich V., Puelles A., Quezada V., Salazar F., Christen P., Castillo J., Cárcamo J.G., Castro-Alvarez A., Nelson R. (2022). Synthesis, Antitumor Activity, 3D-QSAR and Molecular Docking Studies of New Iodinated 4-(3*H*)-Quinazolinones 3N-Substituted. RSC Adv..

[57] Mosmann T. (1983). Rapid Colorimetric Assay for Cellular Growth and Survival: Application to Proliferation and Cytotoxicity Assays. J. Lmmunol. Methods.

[58] González-Calderón D., Santillán-Iniesta I., González-González C.A., Fuentes-Benítes A., González-Romero C. (2015). A Novel and Facile Synthesis of 1,4,5-Trisubstituted 1,2,3-Triazoles from Benzylic Alcohols through a One-Pot, Three-Component System. Tetrahedron Lett..

[59] Shafran Y.M., Beryozkina T.V., Efimov I.V., Bakulev V.A. (2019). Synthesis of β-Azolyl- and β-Azolylcarbonylenamines and Their Reactions with Aromatic Azides. Chem. Heterocycl. Compd..

[60] Jin G., Zhang J., Fu D., Wu J., Cao S. (2012). One-Pot, Three-Component Synthesis of 1,4,5-Trisubstituted 1,2,3-Triazoles Starting from Primary Alcohols. Eur. J. Org. Chem..

[61] Pérez-Ferhmann M., Kesternich V., Cáceres M., Salazar F., Cárdenas A., Cataldo F., Brito I. (2014). Crystal Structure of 1-(1-(2,4-Dichlorobenzyl)-5-Methyl-1*H*-1,2,3-Triazol-4-yl)Ethanone, C_12_H_11_Cl_2_N_3_O. Z. Fur. Krist.—New Cryst. Struct..

[62] Shanmugavelan P., Sathishkumar M., Nagarajan S., Ponnuswamy A. (2012). A Facile Synthesis of 1,2,3-Triazolyl Indole Hybrids via SbCl_3_-Catalysed Michael Addition of Indoles to 1,2,3-Triazolyl Chalcones. J. Chem. Sci..

[63] Bhabha G., Ekiert D.C., Jennewein M., Zmasek C.M., Tuttle L.M., Kroon G., Dyson H.J., Godzik A., Wilson I.A., Wright P.E. (2013). Divergent Evolution of Protein Conformational Dynamics in Dihydrofolate Reductase. Nat. Struct. Mol. Biol..

[64] (2022). Schrödinger Release 2022-1: LigPrep.

[65] Friesner R.A., Murphy R.B., Repasky M.P., Frye L.L., Greenwood J.R., Halgren T.A., Sanschagrin P.C., Mainz D.T. (2006). Extra Precision Glide: Docking and Scoring Incorporating a Model of Hydrophobic Enclosure for Protein-Ligand Complexes. J. Med. Chem..

[66] Halgren T.A., Murphy R.B., Friesner R.A., Beard H.S., Frye L.L., Pollard W.T., Banks J.L. (2004). Glide: A New Approach for Rapid, Accurate Docking and Scoring. 2. Enrichment Factors in Database Screening. J. Med. Chem..

