# Supplementary material for: Ultrasound-Assisted Synthesis of Substituted Chalcone-Linked 1,2,3-Triazole Derivatives as Antiproliferative Agents: In Vitro Antitumor Activity and Molecular Docking Studies
Source: Int J Mol Sci. 2025 Apr 4;26(7):3389. doi: 10.3390/ijms26073389 (PMC11989946; doi:10.3390/ijms26073389)
Supplement: Supplementary file 1 [file ijms-26-03389-s001.zip › ijms-3503117-supplementary.pdf]

SUPPLEMENTARY MATERIALS

Ultrasound assisted synthesis of substituted chalcone linked-1,2,3-triazoles derivatives as antiproliferative agents. *In vitro* antitumor activity and molecular docking studies

Manuel Cáceres[1], Víctor Kesternich[1,*], Marcia Pérez-Fehrmann[1], Mariña Castroagudin[1], Ronald Nelson[1], Víctor Quezada[1], Philippe Christen[2], Alejandro Castro-Alvarez[3,*] and Juan G. Cárcamo[4,5]

1 Departamento de Química, Facultad de Ciencias, Universidad Católica del Norte, Avda. Angamos 0610, Antofagasta 1270709, Chile; manu.caceresv@gmail.com (M.C.), vkestern@ucn.cl (V.K.), maperez@ucn.cl (M.P.), marina.castroagudin@ce.ucn.cl (M.C.), rnelson@ucn.cl (R.N.), victor.quezada@ucn.cl (V.Q.).

2 School of Pharmaceutical Sciences, University of Geneva, University of Lausanne, Rue Michel-Servet 1, CH-1211 Geneva 4, Switzerland; philippe.christen@unige.ch Departamento de Ciencias Preclínicas, Facultad de Medicina, Universidad de La Frontera, Temuco 4811230, Chile; alejandro.castro.a@ufrontera.cl

3 Departamento de Ciencias Preclínicas, Facultad de Medicina, Universidad de La Frontera, Temuco 4811230, Chile; alejandro.castro.a@ufrontera.cl

4 Instituto de Bioquímica y Microbiología, Facultad de Ciencias, Universidad Austral de Chile, Valdivia. Chile, gcarcamo@uach.cl

5 Centro FONDAP, Interdisciplinary Center for Aquaculture Research (INCAR), Chile. gcarcamo@uach.cl

* Correspondence: vkestern@ucn.cl (V.K.) and alejandro.castro.a@ufrontera.cl (A.C-A.)

General contents

1. General information 2

2. Synthesis of benzyl acetyl triazole derivatives **2a-d** 3

3. Cytotoxicity assay 5

4. ^{1}H NMR, ^{13}C NMR/APT, HSQC, HMBC spectra of compounds. 26

1. General information

Melting points were determined on a Stuart SMP3 apparatus and are uncorrected. IR spectra were recorded on a Perkin-Elmer FT-IR Spectrometer Spectrum Two with KBr. NMR spectra were acquired in DMSO-d_6 or $CDCl_3$ with a Varian Unity Inova 500 MHz spectrometer. Chemical shifts are reported in parts per million (δ) relative to the residual solvent signals (DMSO-d_6: δ_H 2.50, δ_C 39.5 or $CDCl_3$; δ_H H 7.26, δ_C C 77.2) as internal standards for ^{1}H and ^{13}C NMR spectra. Coupling constants (*J*) are expressed in Hz. HRMS spectra were recorded using a Micromass-LCT Premier Time-of-Flight ESI spectrometer coupled with an ACQUITY UHPLC (ultra-high-performance liquid chromatography) interface system. The reactions were monitored by thin-layer chromatography (TLC) performed on silica gel Merck 60 F_{254}. The components were visualized under UV light (254 and 365 nm), and/or by treatment with phosphomolybdic acid reagent followed by heating. All starting materials and reagents were obtained from commercial suppliers.

Compounds **2a** [1], **2b** [2], **2c** [3], **2d** [4], **4m** [5], **4n** [5], **4o** [5] and **4p** [5] are known and their spectroscopic data were previously reported in the references indicated.

2. Synthesis of benzyl acetyl triazole derivatives 2a-d

General procedure for synthesis of the benzyl acetyl triazole derivatives (BAT). (exemplified for the synthesis of **2a**):

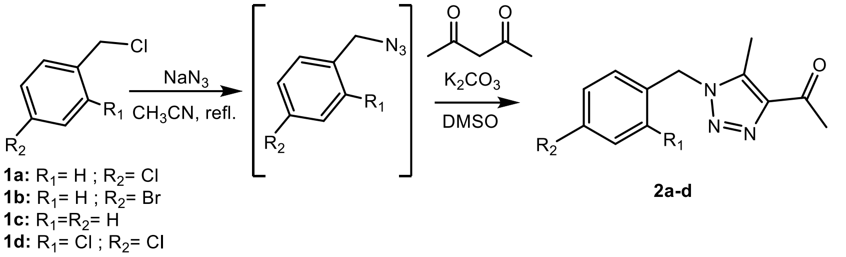

The experimental procedure was adapted from Chen *et al*. [6] and Nelson *et al*.[7]: A mixture of 12.0 g (74.5 mmol) of 4-chlorobenzyl chloride and 6.29 g (96.8 mmol) of sodium azide in 150 mL of acetonitrile was refluxed for 72 h. The reaction mixture was allowed to reach room temperature and 50 mL of water was added, followed by liquid-liquid extraction with chloroform (3 x 40 mL). The organic phase was dried over anhydrous sodium sulfate and concentrated under reduced pressure. The resulting viscous-yellowish intermediates were re-dissolved in 70 mL of DMSO, followed by the addition of 22.17 mL (214.8 mmol) of acetylacetone and 29.7 g (214.8 mmol) of potassium carbonate (K_2CO_3). The resulting mixture was stirred at room temperature for 48 h until reaction completion was confirmed by thin-layer chromatography. Then, 60 mL of saturated sodium chloride solution was added, and the resulting precipitate was filtered, washed with cold water, and dried. The product was purified by crystallization from ethyl acetate.

*1- (1-(4-chlorobenzyl) -5-methyl-1H-1,2,3-triazol-4-yl)-1-ethanone **(2a).***

Yellow crystals, m.p.: 65-66 °C (Lit. 63-65 °C [1]). (Rf: 0.564 eluent: hexane / AcOEt,1/1). IR (cm^{-1}) ν: 3063 and 3026 (CAr-H); 2967 and 2926 (Csp3-H); 1684 (C=O); 1597 (CAr-CAr); 1554 (N=N). ^{1}H-NMR ($CDCl_3$, 500 MHz) δ (ppm): 2.47 (3H, s), 2.69 (3H, s), 5.47 (2H, s), 7.11 (2H, d, *J*= 8.44 Hz), 7.32 (1H, d, *J*= 8.43 Hz). ^{13}C-NMR ($CDCl_3$, 125.7 MHz) δ (ppm): 9.3 (CH_3), 27.8 (CH_3), 51.1 (CH_2), 128.8 (2CH), 129.5 (2CH), 132.5 (C), 134.8 (C), 136.9 (C), 144.2 (C), 194.5 (CO). HRMS-ESI: m/z 250.07248 for $C_{12}H_{13}N_3OCl$ $[M+H]^+$, calculated: m/z: 250.07470.

*1- (1-(4-bromobenzyl)-5-methyl-1H-1,2,3-triazol-4-yl)-1-ethanone **(2b).***

Light-brown solid, m.p.: 89-90 °C (Lit. 86-87 °C [2]). IR (cm^{-1}) ν: 3063 y 3026 (CAr-H); 2988 and 2967 (Csp3-H); 1684 (C=O); 1592 (CAr-CAr); 1554 (N=N). ^{1}H-NMR ($CDCl_3$, 500 MHz) δ (ppm): 2.47 (3H, s), 2.69 (3H, s), 5.46 (2H, s), 7.06 (2H, d, *J*= 8.3 Hz), 7.48 (2H, d, *J*= 8.4 Hz). ^{13}C-NMR ($CDCl_3$, 125.7 MHz) δ (ppm): 9.3 (CH_3), 27.9 (CH_3), 51.2 (CH_2), 123.0 (C), 129.1 (2CH), 132.5 (2CH), 133.1 (C), 136.9 (C), 144.2 (C), 194.6 (C). HRMS-ESI: m/z 294.02307 for $C_{12}H_{12}N_3OBr$ $[M+H]^+$, calculated: m/z 294.02419.

*1- (1-benzyl-5-methyl-1H-1,2,3-triazol-4-yl)-1-ethanone **(2c).***

Yellow oil. IR (cm^{-1}) ν: 3067 and 3039 (CAr-H); 2958 and 2923 (Csp3-H); 1675 (C=O); 1586 (CAr-CAr); 1559 1562 (N=N). ^{1}H-NMR ($CDCl_3$, 500 MHz) δ (ppm): 2.50 (3H, s), 2.70 (3H, s), 5.58 (2H, s), 6.79 (1H, d, *J*= 8.3 Hz), 7.21 (1H, d, *J*= 8.4 Hz), 7.46 (1H, s). ^{13}C-NMR ($CDCl_3$, 125.7 MHz) δ (ppm): 9.2 (CH_3), 27.8

(CH_3), 48.2 (CH_2), 128.1 (2CH), 129.7 (CH), 129.9 (CH), 130.6 (C), 133.4 (C), 135.4 (CH), 137.3 (C), 144.0 (C), 194.4 (C). HRMS-ESI: m/z 216.1167 for $C_{12}H_{14}N_3O$ $[M+H]^+$, calculated: m/z 216.11368.

1-(1-(2,4-dichlorobenzyl)-5-methyl-1H-1,2,3-triazol-4-yl)-1-ethanone ***(2d).***

Yellow crystals, m.p.: 111-112 °C (Lit. 111 °C [4]). IR (cm^{-1}) ν: 3076 (CAr-H); 2974 and 2924 (Csp3-H); 1680 (C=O); 1588 (CAr-CAr); 1562 (N=N). ^{1}H-NMR ($CDCl_3$, 500 MHz) δ (ppm): 2.50 (3H, s); 2.70 (3H, s); 5.58 (2H, s); 6.79 (1H, d, *J*= 8.3 Hz); 7.21 (1H, d, *J*= 8.4 Hz); 7.46 (1H, s). ^{13}C-NMR ($CDCl_3$, 125.70 MHz) δ (ppm): 9.2 (CH_3); 27.8 (CH_3); 48.21 (CH_2); 128.1 (2CH); 129.7 (CH); 129.9 (CH); 130.6 (C); 133.4 (C); 135.4 (CH); 137.3 (C); 144.0 (C); 194.4 (C). HRMS-ESI: m/z 284.03560 for $C_{12}H_{12}N_3OCl_2$ $[M+H]^+$, calculated: m/z 284.03573.

1. González-Calderón, D.; Santillán-Iniesta, I.; González-González, C.A.; Fuentes-Benítes, A.; González-Romero, C. A Novel and Facile Synthesis of 1,4,5-Trisubstituted 1,2,3-Triazoles from Benzylic Alcohols through a One-Pot, Three-Component System. *Tetrahedron Lett* **2015**, *56*, 514–516, doi:10.1016/j.tetlet.2014.12.019.

2. Shafran, Y.M.; Beryozkina, T. V.; Efimov, I. V.; Bakulev, V.A. Synthesis of β-Azolyl- and β-Azolylcarbonylenamines and Their Reactions with Aromatic Azides. *Chem. Heterocycl. Compd. (N Y)* **2019**, *55*, 704–715, doi:10.1007/s10593-019-02525-2.

3. Jin, G.; Zhang, J.; Fu, D.; Wu, J.; Cao, S. One-Pot, Three-Component Synthesis of 1,4,5-Trisubstituted 1,2,3-Triazoles Starting from Primary Alcohols. *Eur. J. Org. Chem.* **2012**, 5446–5449, doi:10.1002/ejoc.201200830.

4. Pérez-Ferhmann, M.; Kesternich, V.; Cáceres, M.; Salazar, F.; Cárdenas, A.; Cataldo, F.; Brito, I. Crystal Structure of 1-(1-(2,4-Dichlorobenzyl)-5-Methyl-1*H*-1,2,3-Triazol-4-yl)Ethanone, $C_{12}H_{11}Cl_2N_3O$. *Zeitschrift fur Kristallographie - Z. Kristallogr. - New Cryst. Struct.* **2014**, *229*, 367–368, doi:10.1515/ncrs-2014-0191.

5. Shanmugavelan, P.; Sathishkumar, M.; Nagarajan, S.; Ponnuswamy, A. A Facile Synthesis of 1,2,3-Triazolyl Indole Hybrids via $SbCl_3$-Catalysed Michael Addition of Indoles to 1,2,3-Triazolyl Chalcones. *J. Chem. Sci.* **2012**, *124*, 941–950, doi:10.1007/s12039-012-0281-x.

6. Chen, X.B.; Shi, D.Q. Synthesis and Biological Activity of Novel Phosphonate Derivatives Containing of Pyridyl and 1,2,3-Triazole Rings. *Phosphorus Sulfur Silicon Relat. Elem.* **2008**, *183*, 1134–1144, doi:10.1080/10426500701578522.

7. Nelson, R.; Kesternich, V.; Pérez-Fehrmann, M.; Jaldin, S.; Marcourt, L.; Christen, P. Regiospecific Synthesis of 1,4,5-Trisubstituted 1,2,3-Triazoles via Enolate-Azide Cycloaddition between 1,3-Dicarbonyl Compounds and Aryl Azides. *J. Chem. Res.* **2016**, *40*, 453–457, doi:10.3184/174751916X14656662266973.

3. Cytotoxicity assay

Compound 4a

U937

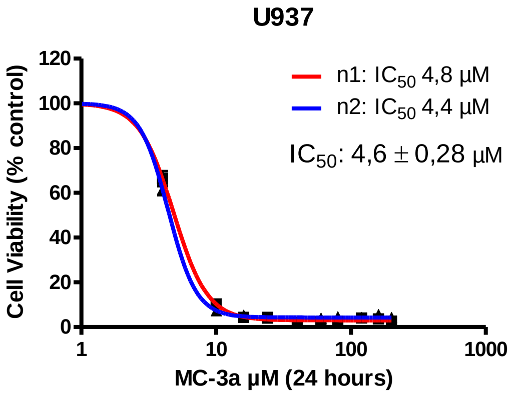

T98G

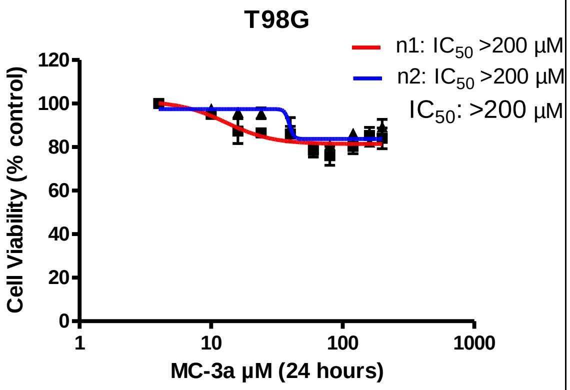

Gb-d1

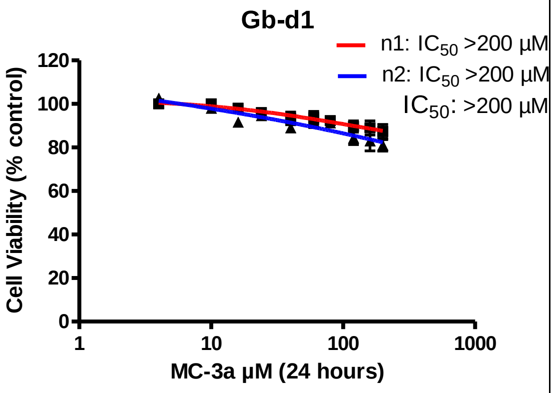

Hela

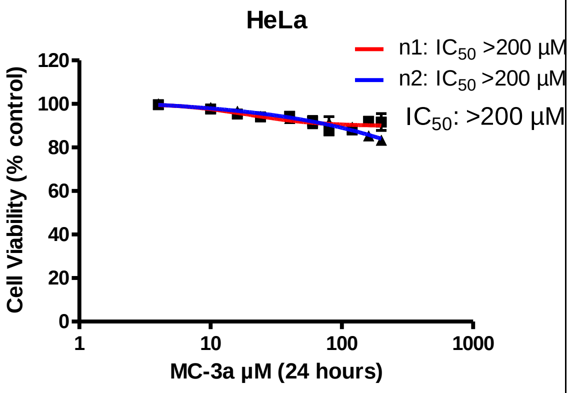

G-415

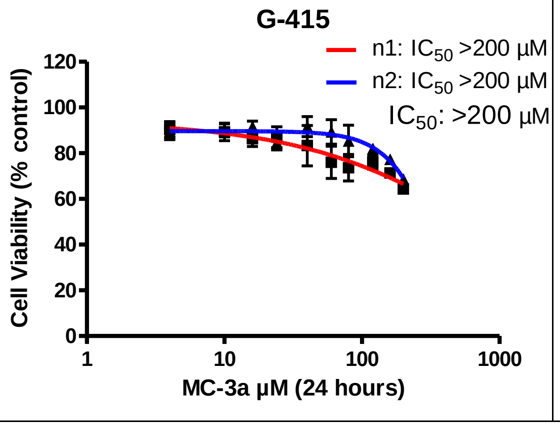

Figure S1: Cytotoxicity curves of compound 4a

Compound 4b

U937

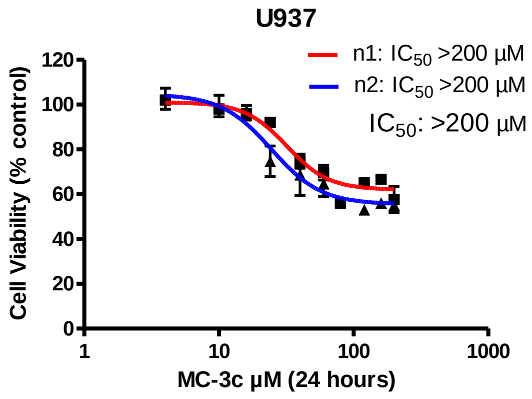

T98G

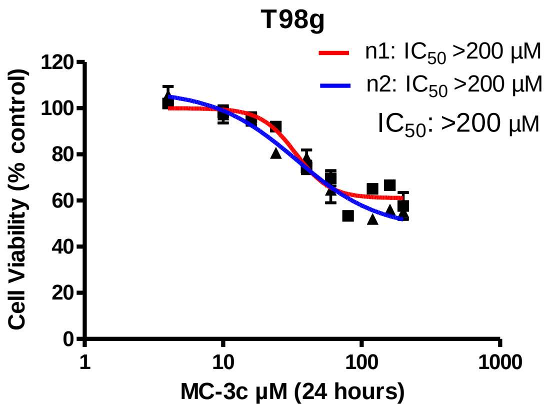

Gb-d1

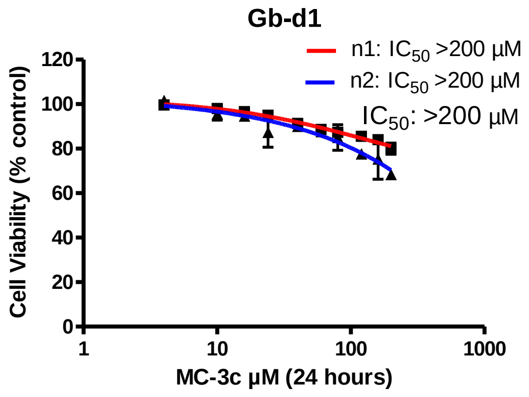

Hela

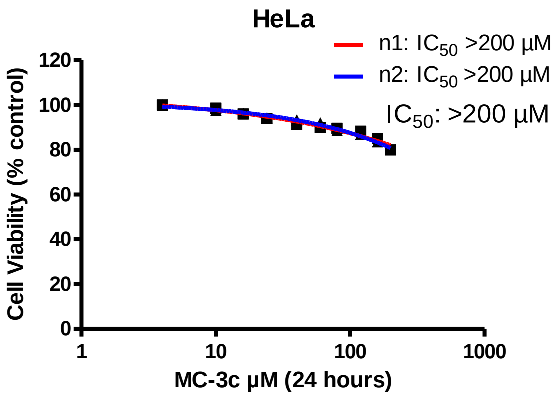

G-415

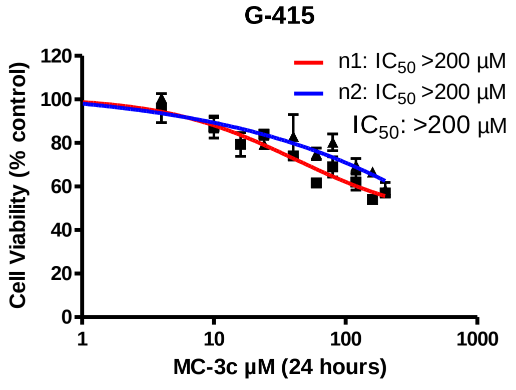

Figure S2: Cytotoxicity curves of compound 4b

Compound 4c

U937	T98G
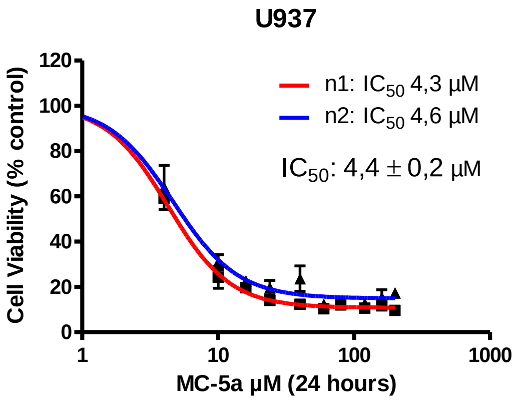	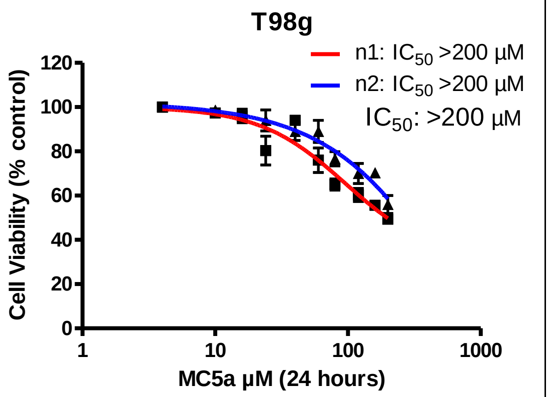
**Gb-d1**	**Hela**
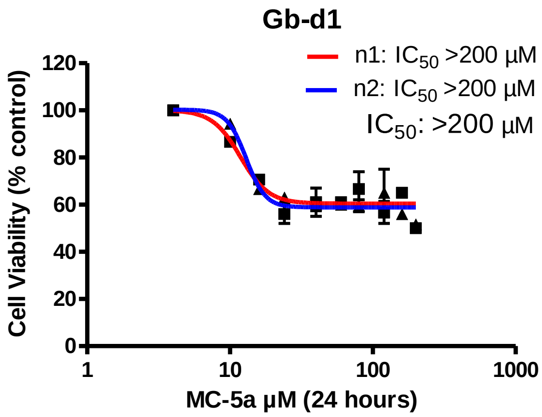	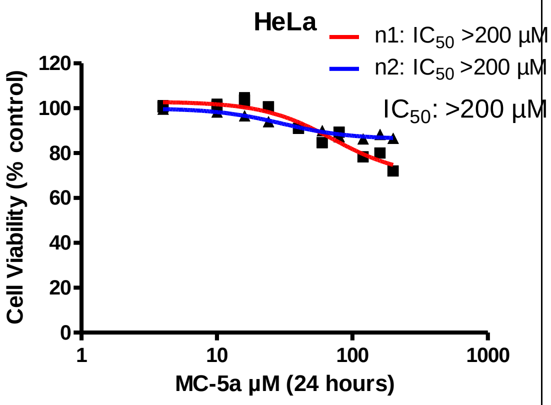
**G-415**	
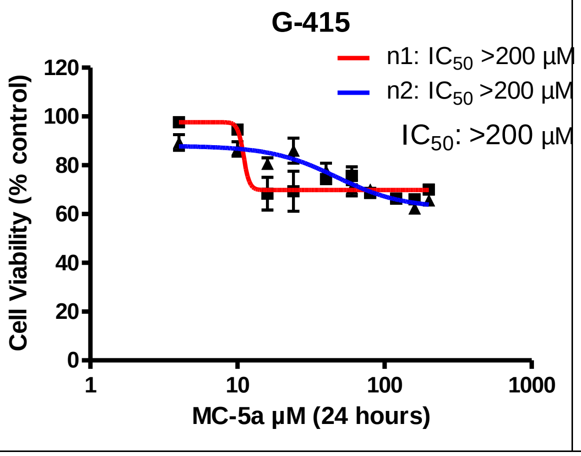	

Figure S3: Cytotoxicity curves of compound 4c

Compound 4d

U937

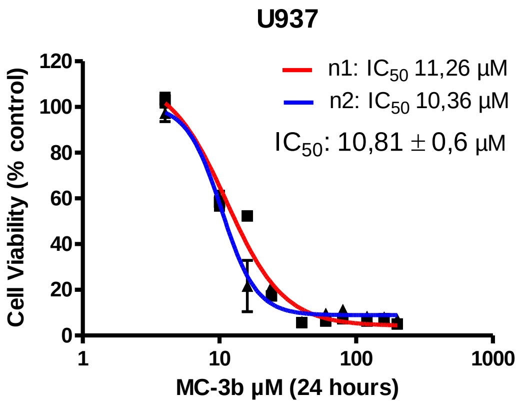

T98G

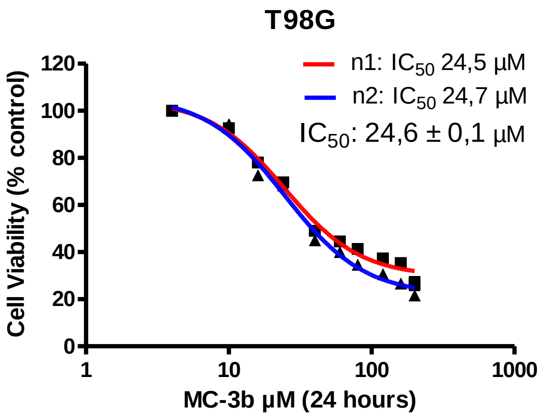

Gb-d1

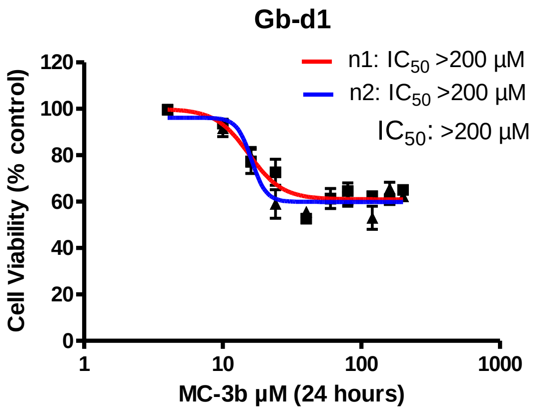

Hela

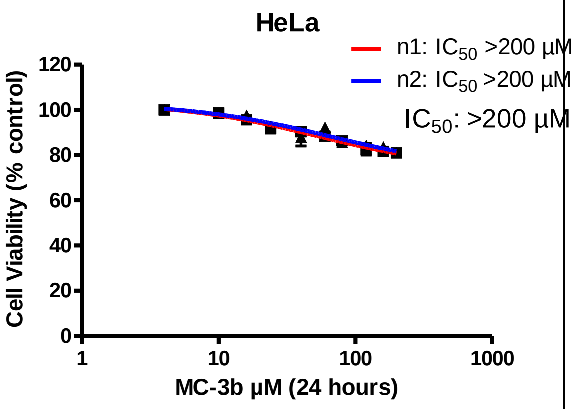

G-415

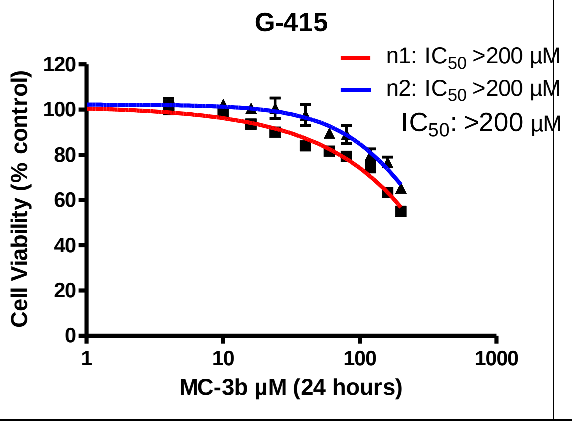

Figure S4: Cytotoxicity curves of compound 4d

Compound 4e

U937

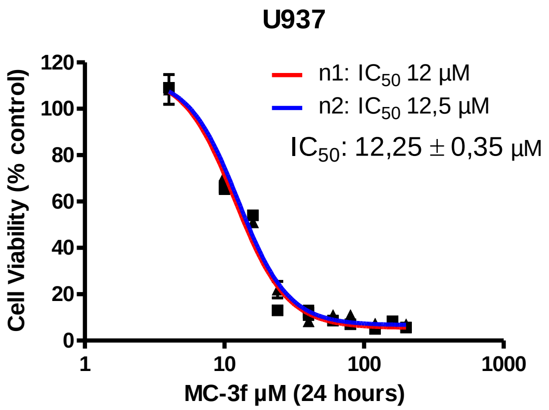

T98G

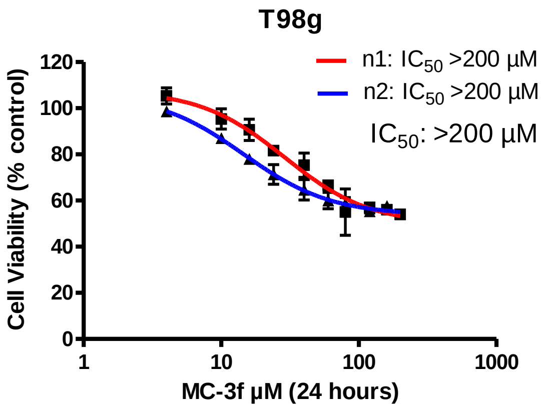

Gb-d1

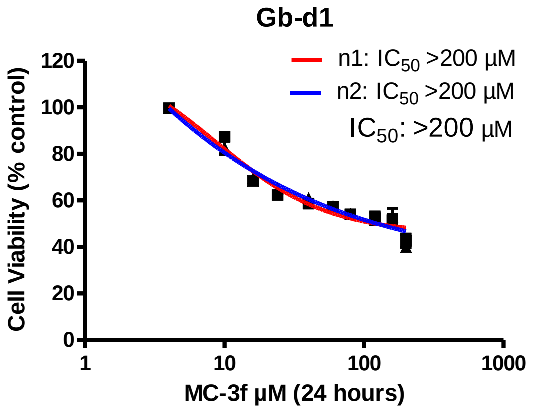

Hela

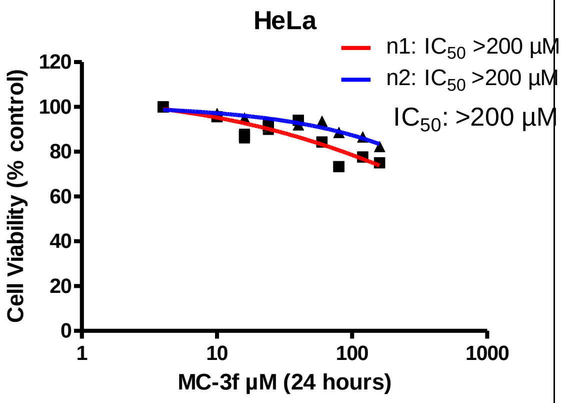

G-415

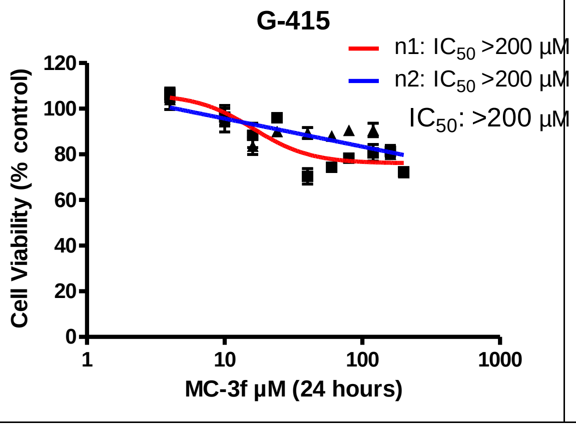

Figure S5: Cytotoxicity curves of compound 4e

Compound 4f

U937	T98G
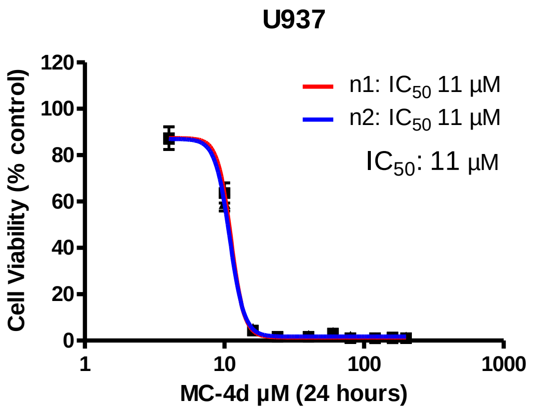	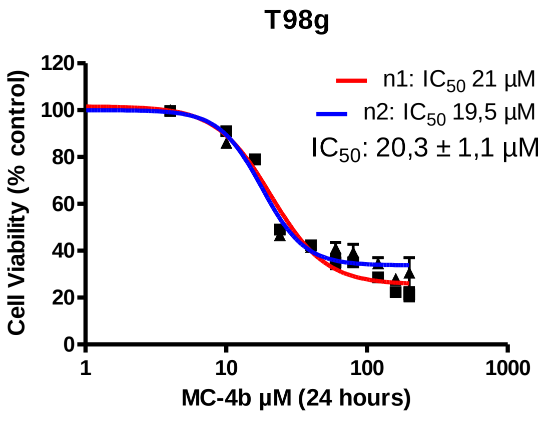
**Gb-d1**	**Hela**
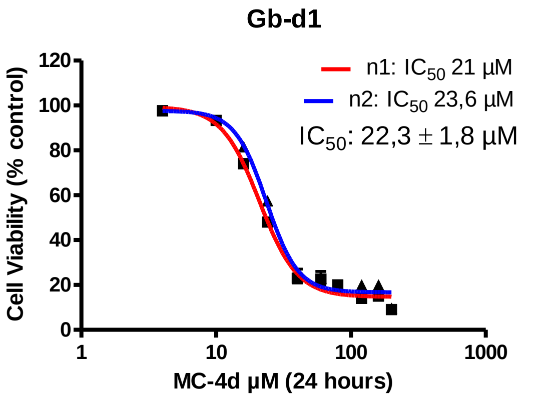	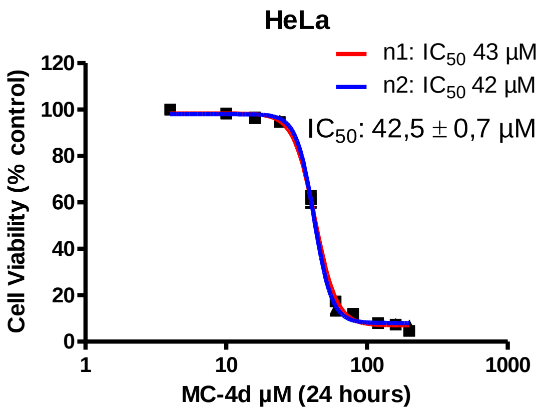
**G-415**	
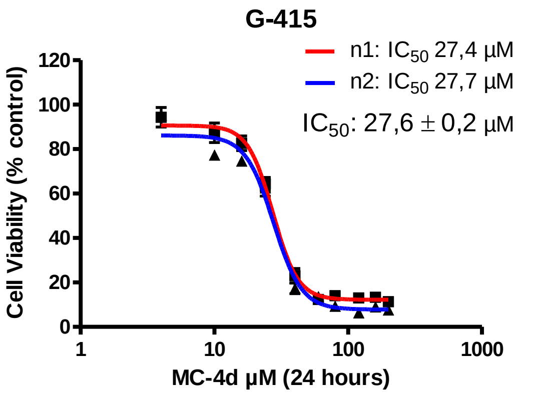	

Figure S6: Cytotoxicity curves of compound 4f

Compound 4g

U937

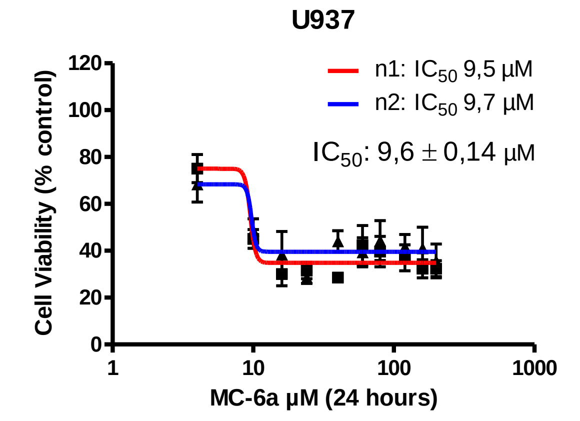

T98G

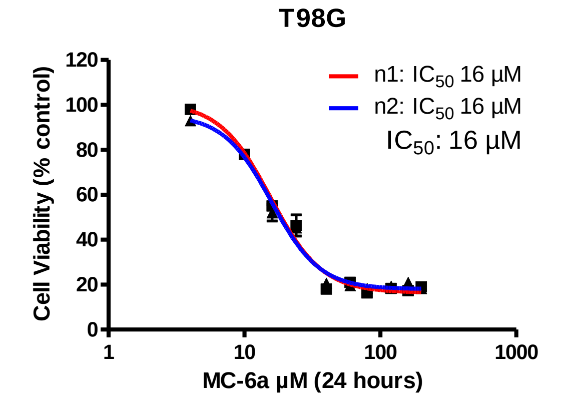

Gb-d1

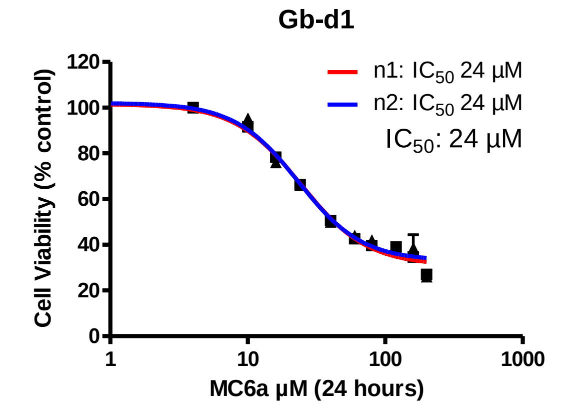

Hela

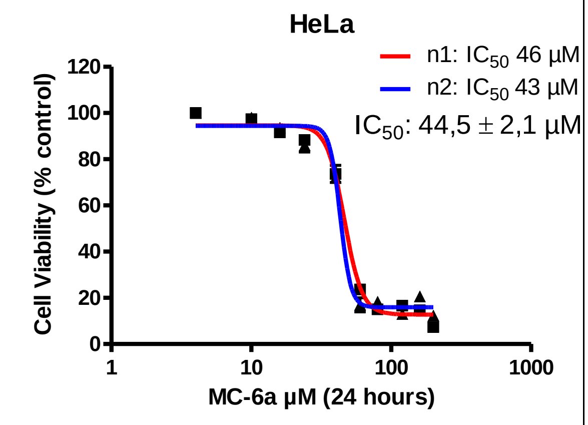

G-415

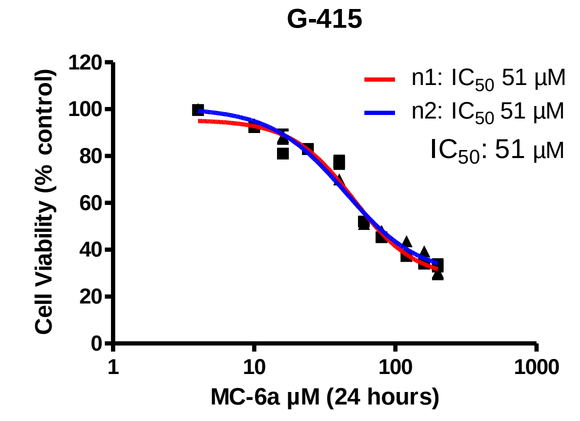

Figure S7: Cytotoxicity curves of compound 4g

Compound 4h

U937	T98G
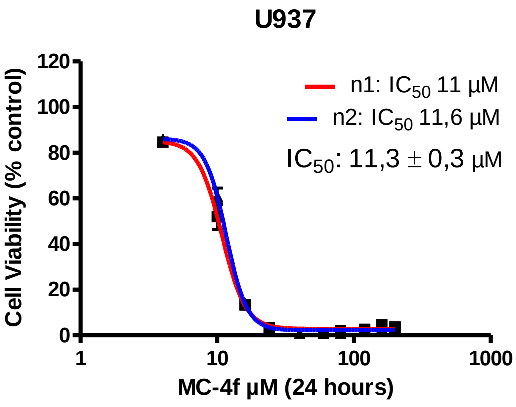	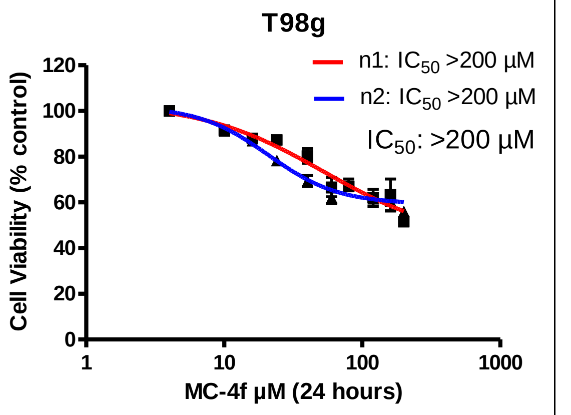
**Gb-d1**	**Hela**
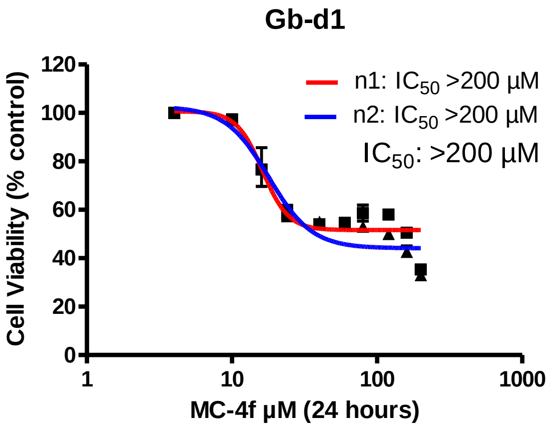	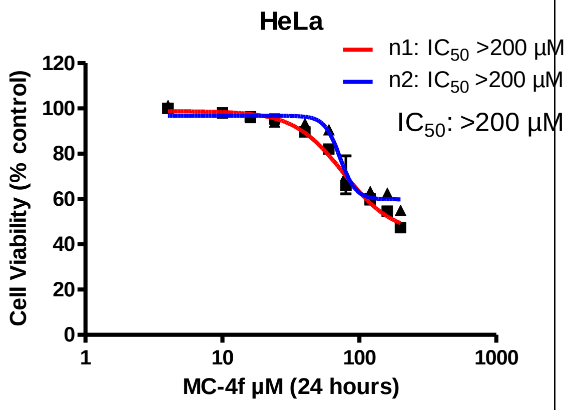
**G-415**	
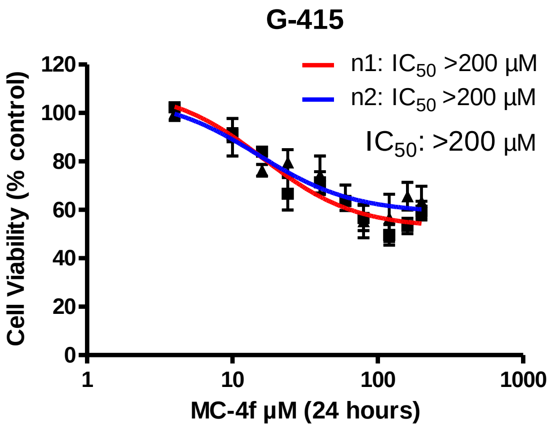	

Figure S8: Cytotoxicity curves of compound 4h

Compound 4i

U937	T98G
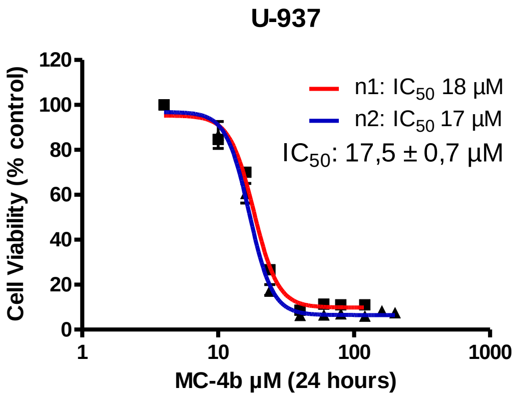	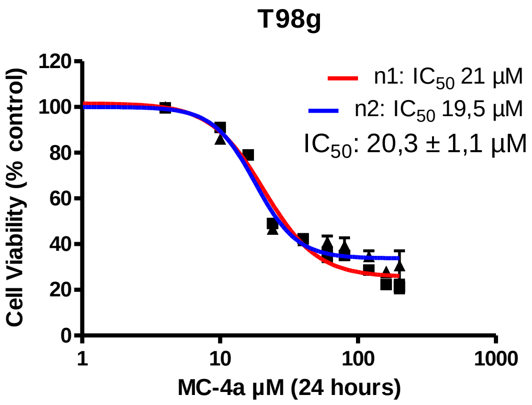
**Gb-d1**	**Hela**
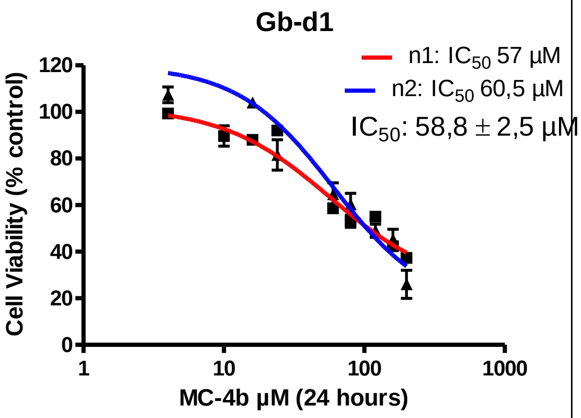	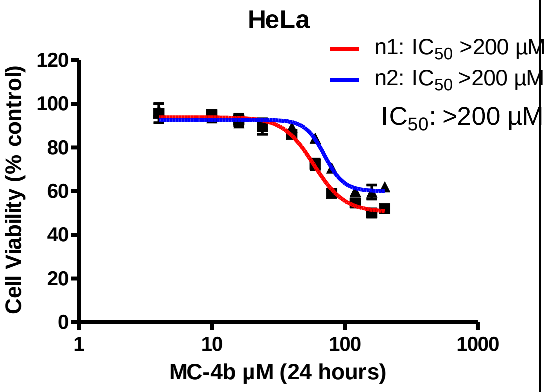
**G-415**	
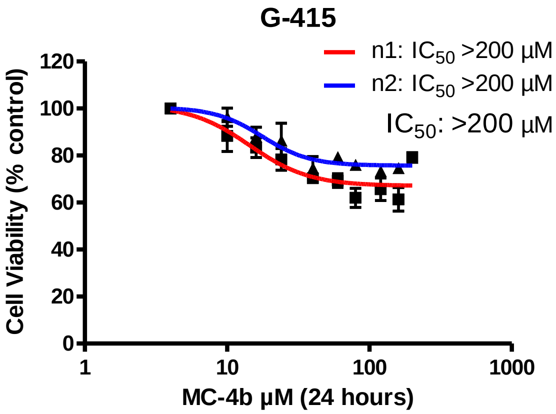	

Figure S9: Cytotoxicity curves of compound 4i

Compound 4j

U937

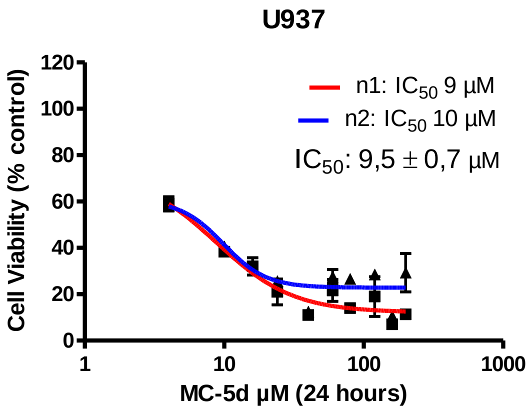

T98G

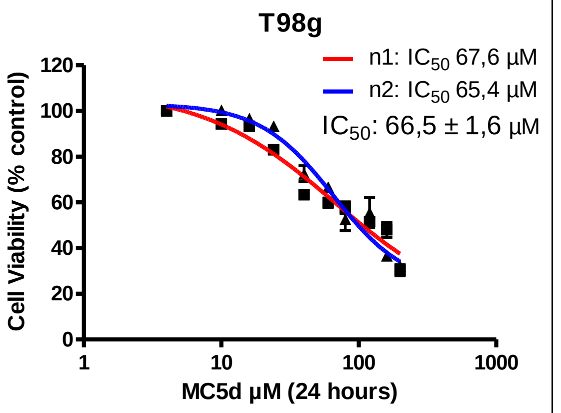

Gb-d1

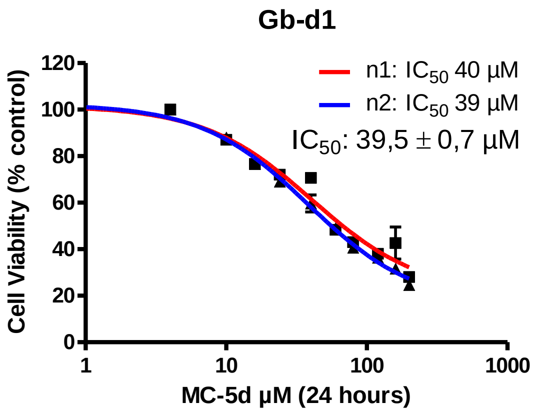

Hela

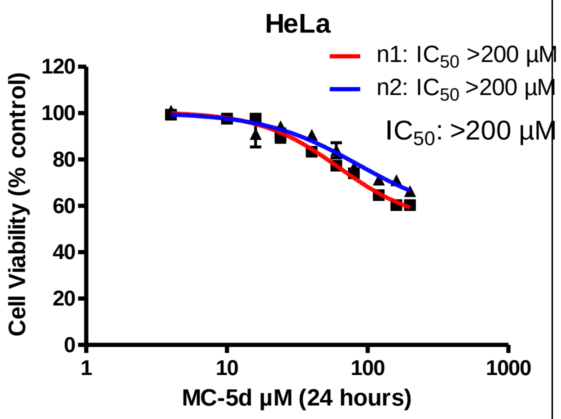

G-415

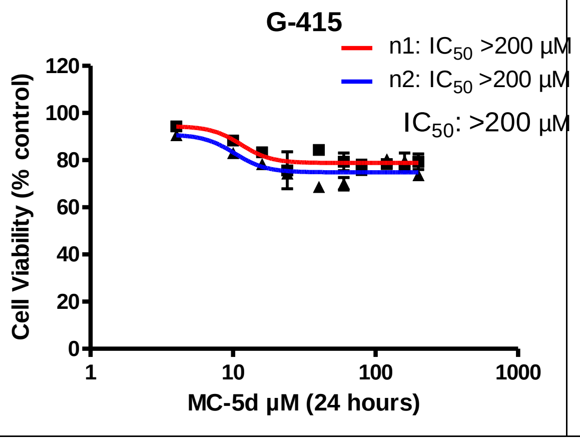

Figure S10: Cytotoxicity curves of compound 4j

Compound 4k

U937

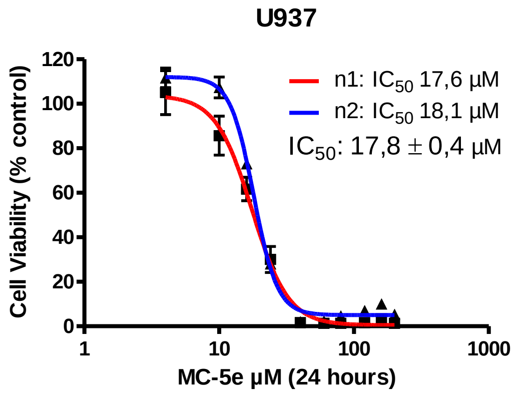

T98G

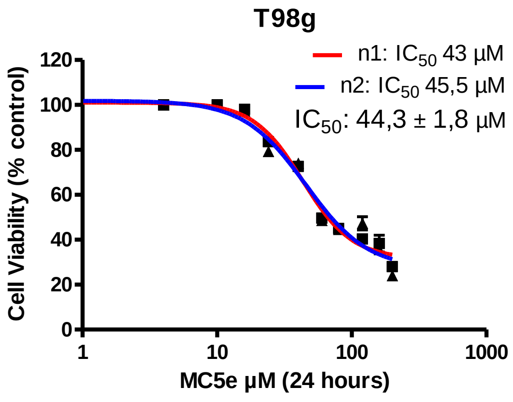

Gb-d1

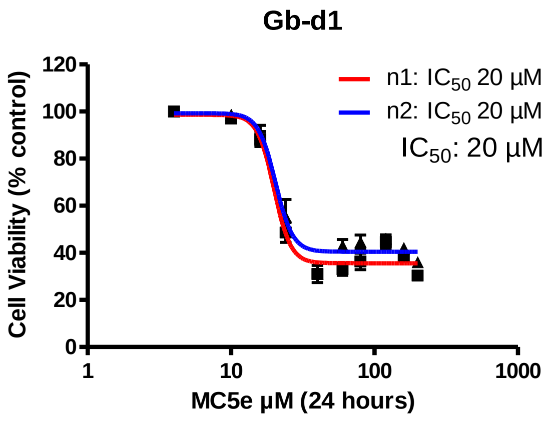

Hela

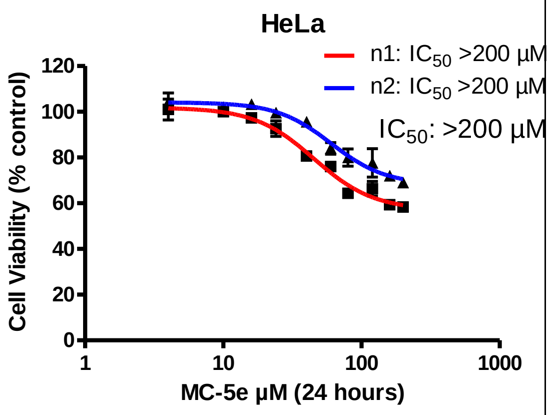

G-415

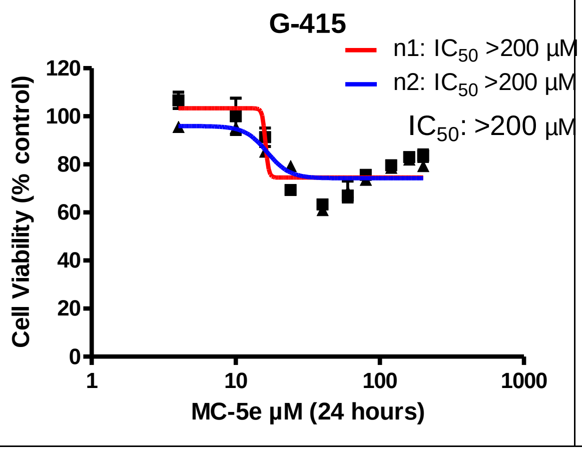

Figure S11: Cytotoxicity curves of compound 4k

Compound 4l

U937	T98G
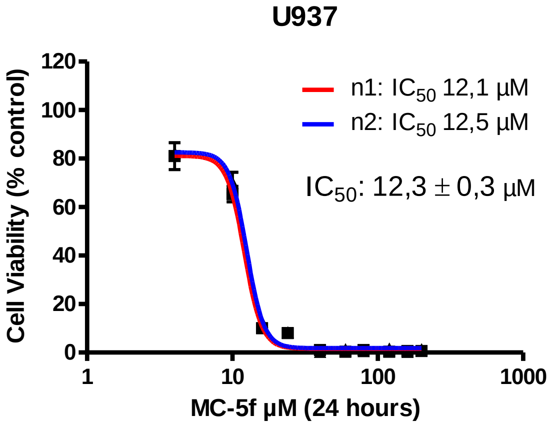	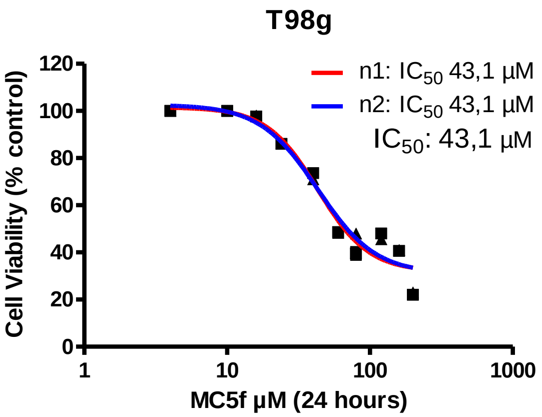
**Gb-d1**	**Hela**
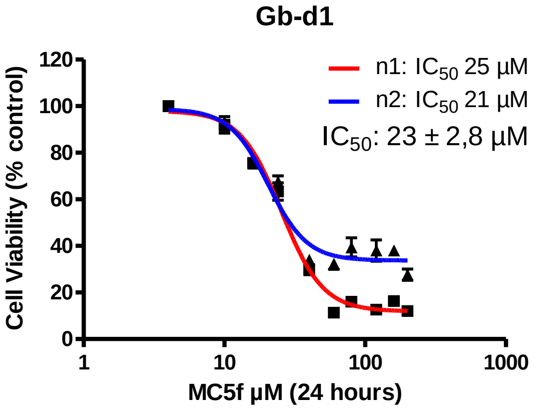	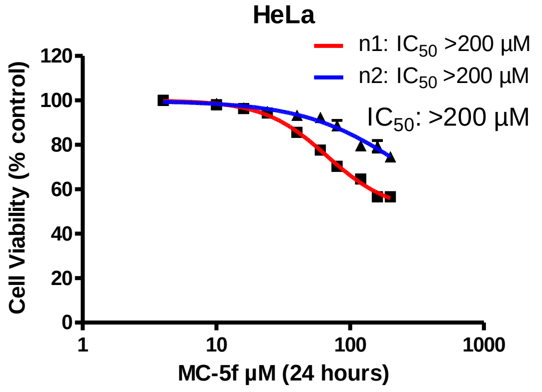
**G-415**	
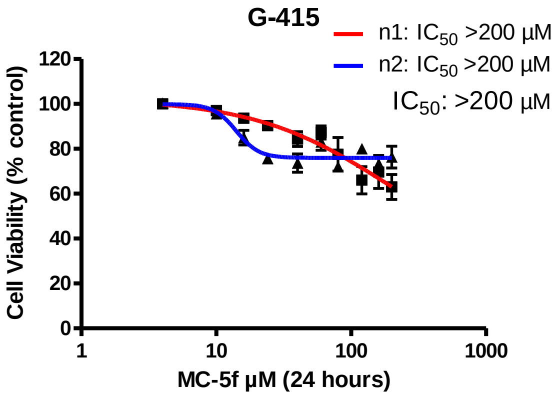	

Figure S12: Cytotoxicity curves of compound 4l

Compound 4m

U937	T98G
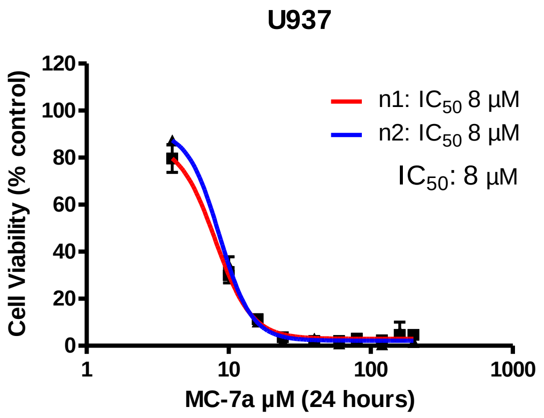	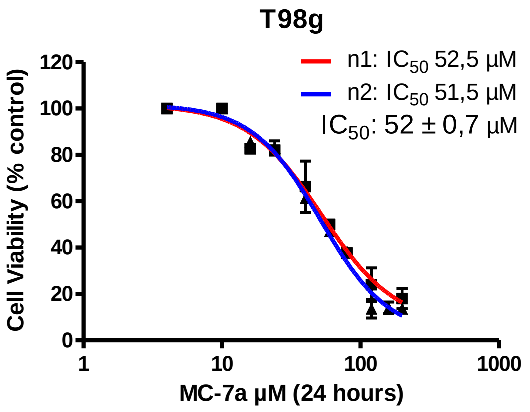
**Gb-d1**	**Hela**
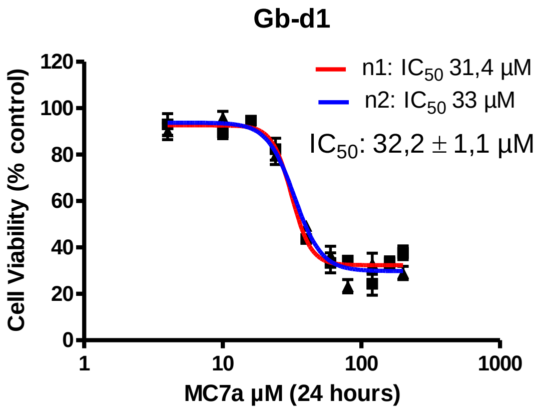	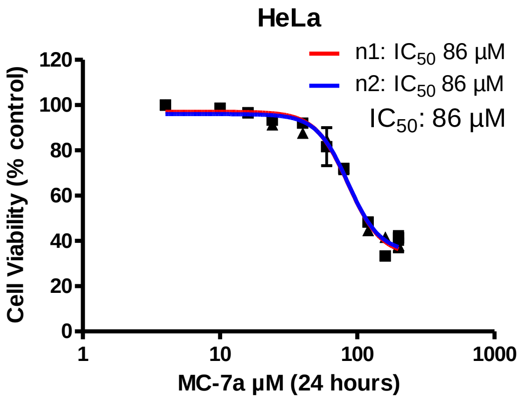
**G-415**	
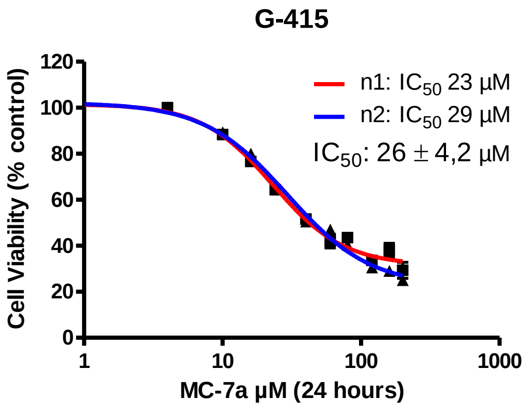	

Figure S13: Cytotoxicity curves of compound 4m

Compound 4n

U937	T98G
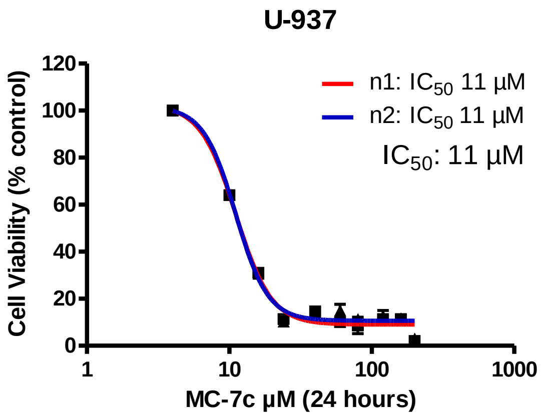	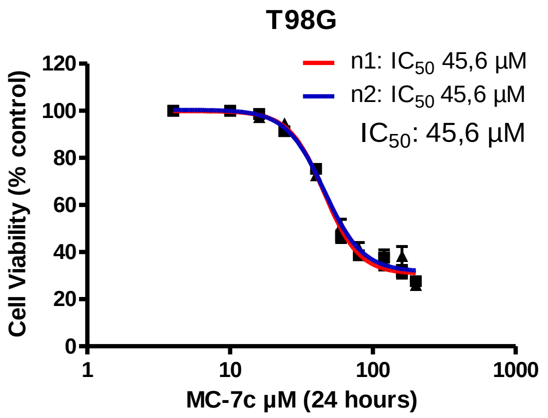
**Gb-d1**	**Hela**
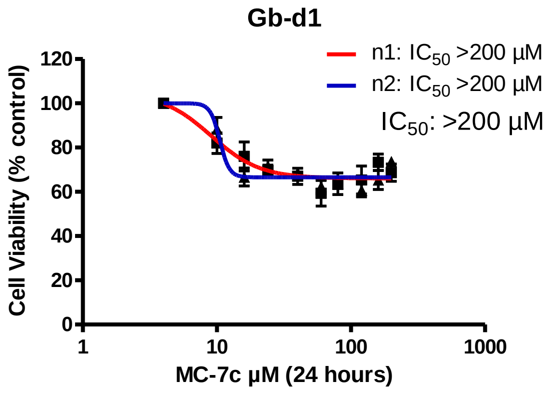	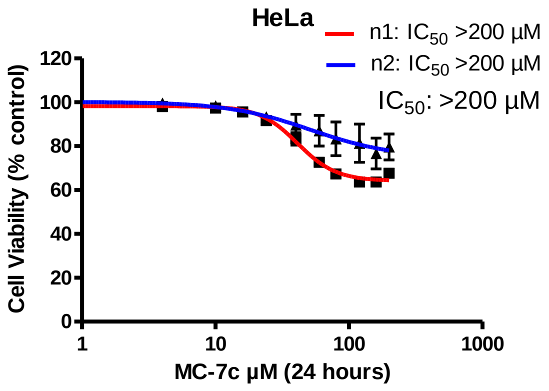
**G-415**	
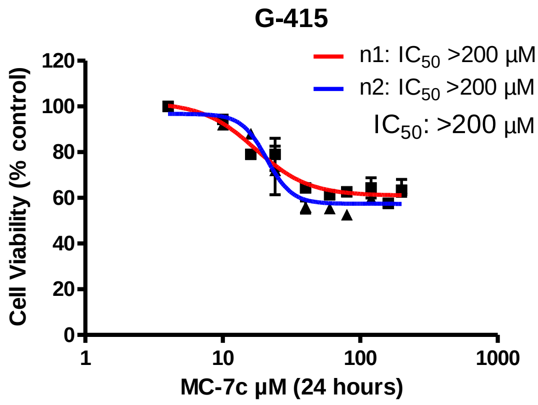	

Figure S14: Cytotoxicity curves of compound 4n

Compound 4o

U937	T98G
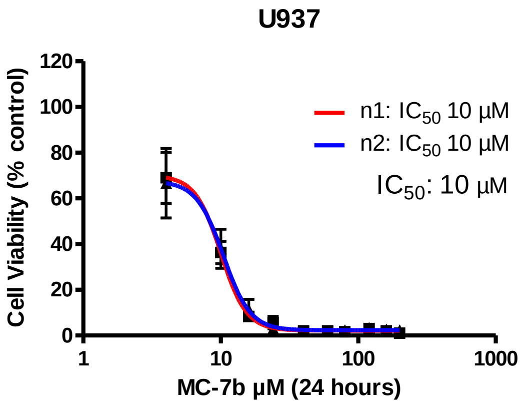	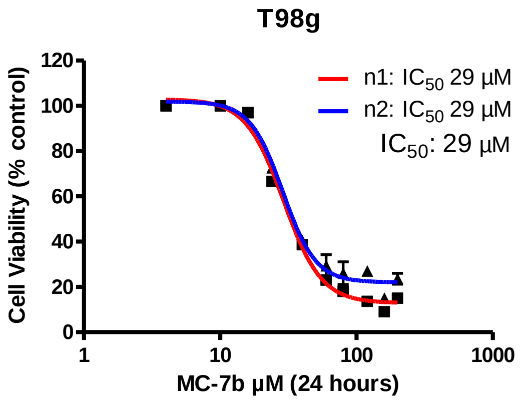
**Gb-d1**	**Hela**
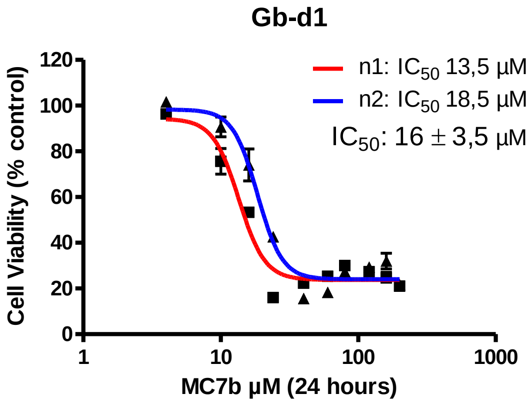	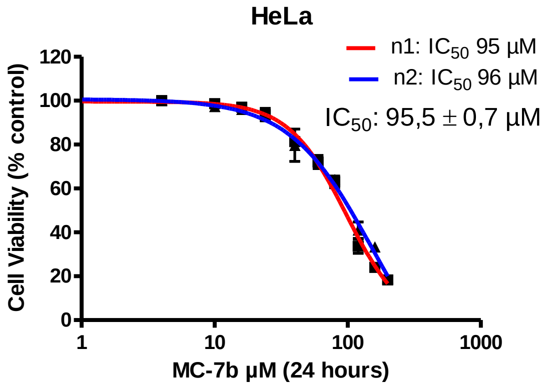
**G-415**	
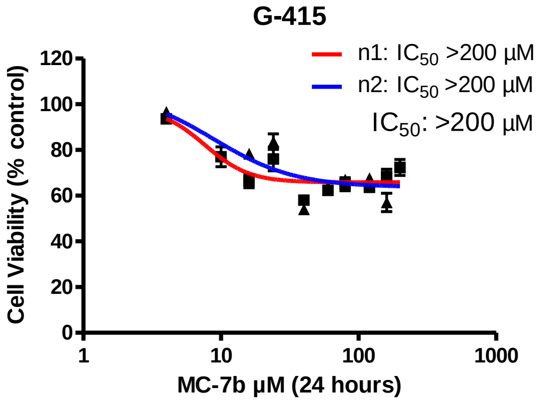	

Figure S15: Cytotoxicity curves of compound 4o

Compound 4p

U937	T98G
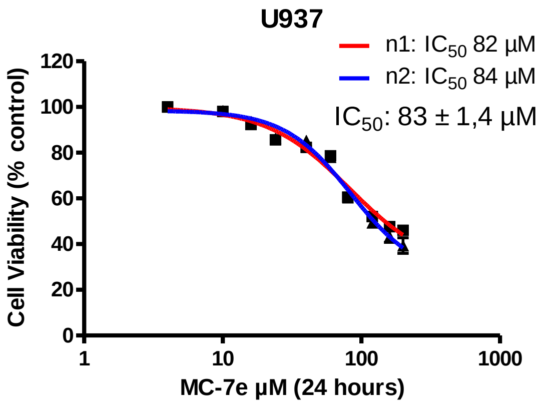	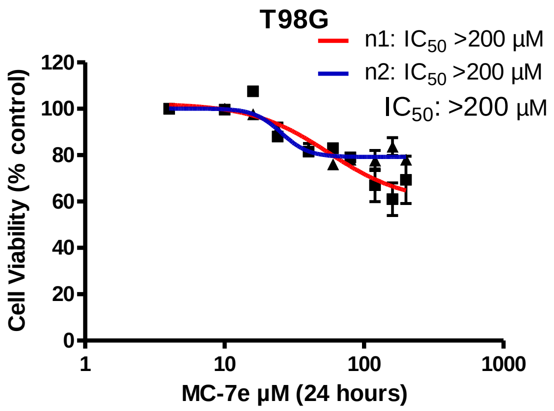
Gb-d1	Hela
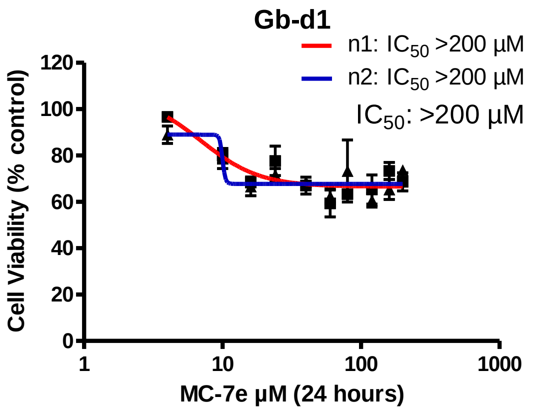	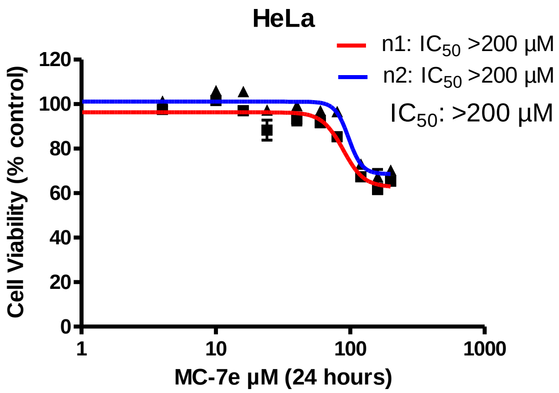
G-415	
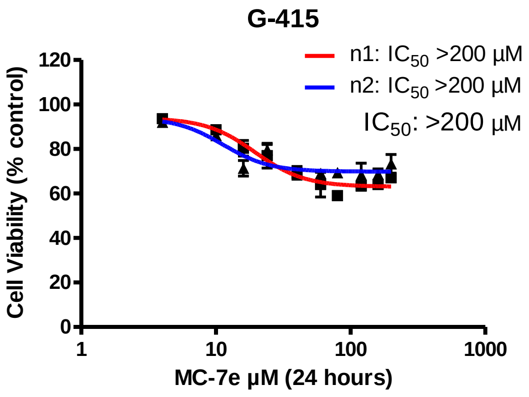	

Figure S16: Cytotoxicity curves of compound 4p

Compound 4q

U937

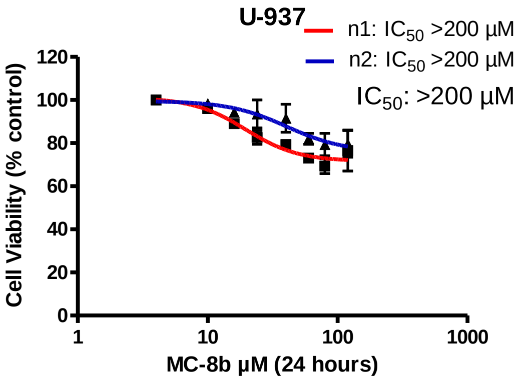

T98G

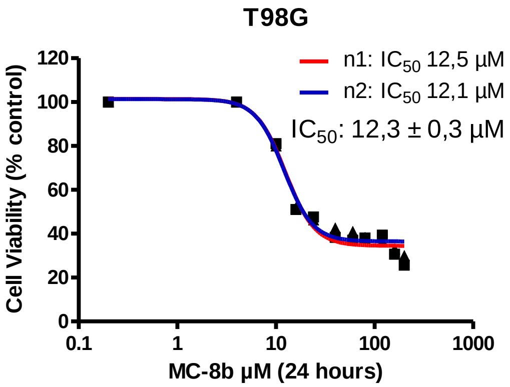

Gb-d1

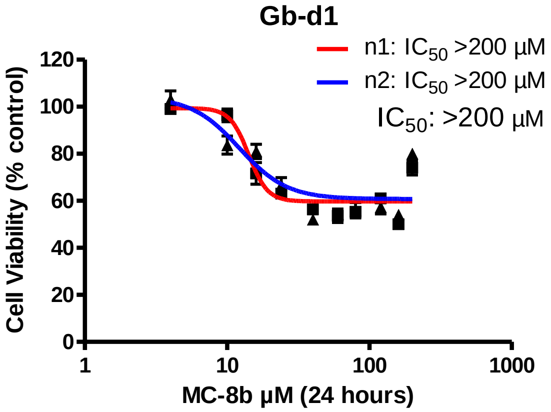

Hela

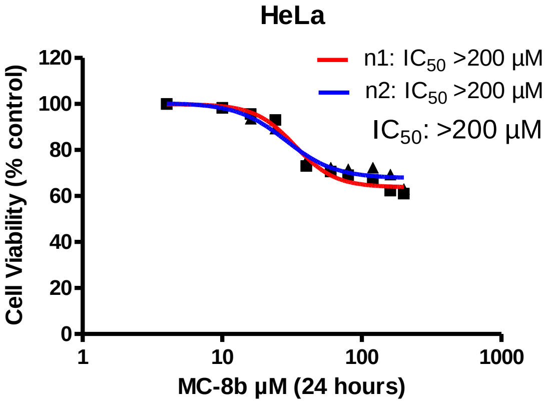

G-415

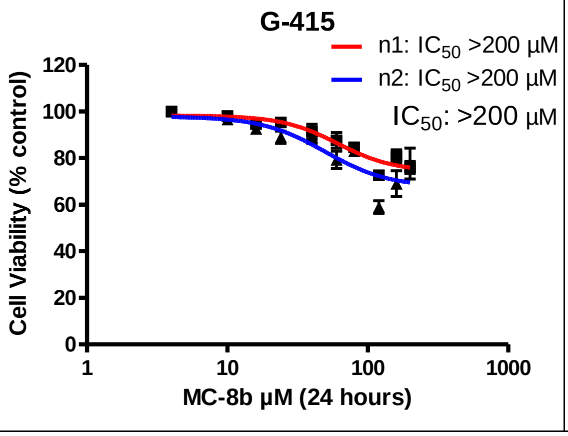

Figure S17: Cytotoxicity curves of compound 4q

Compound 4r

U937	T98G
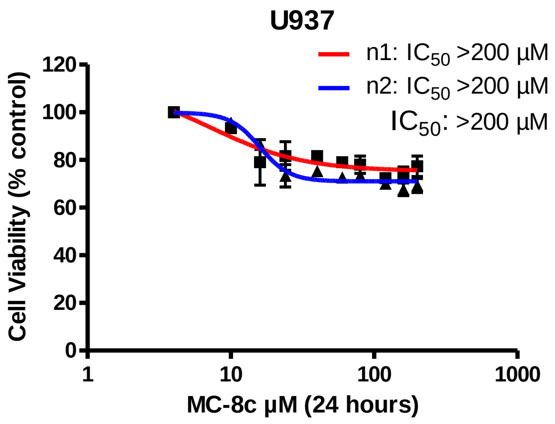	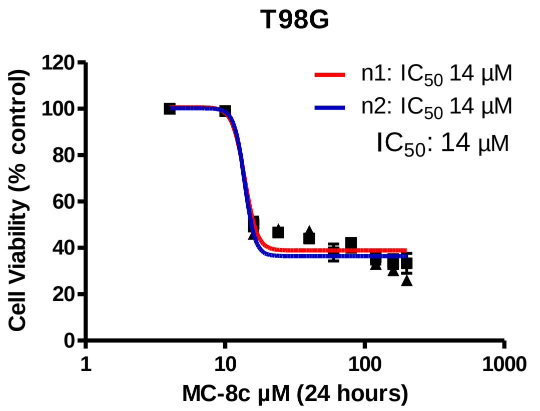
**Gb-d1**	**Hela**
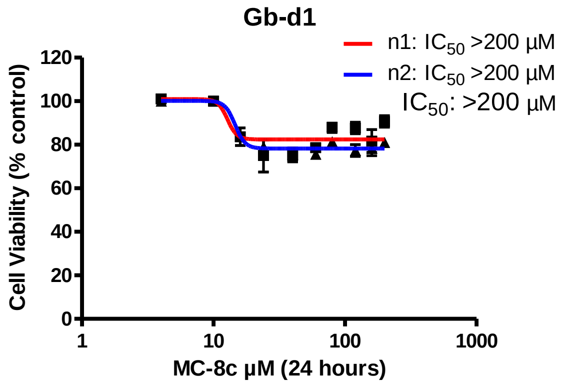	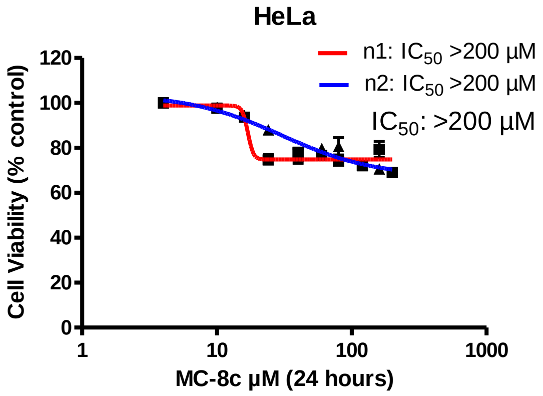
**G-415**	
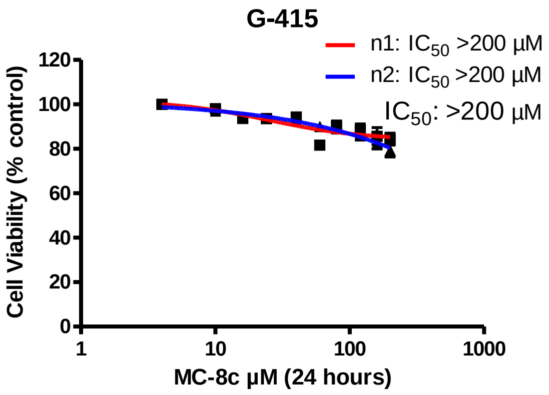	

Figure S18: Cytotoxicity curves of compound 4r

Compound 4s

U937

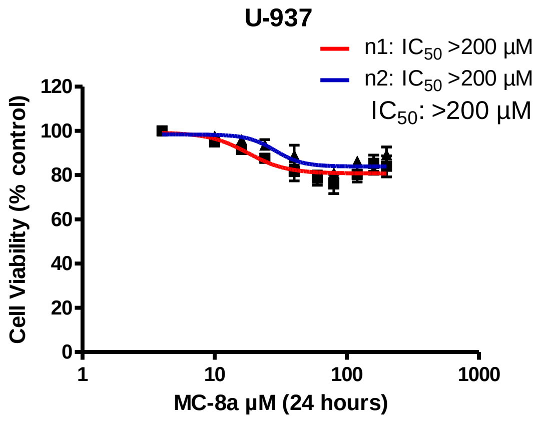

T98G

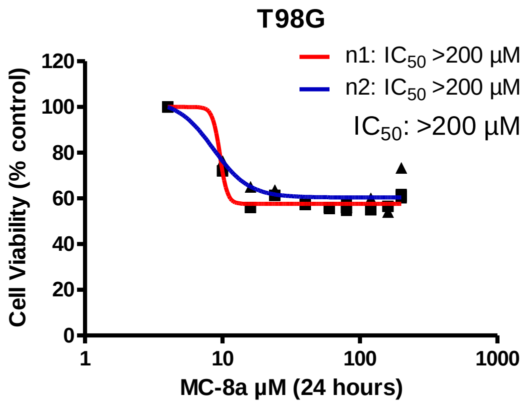

Gb-d1

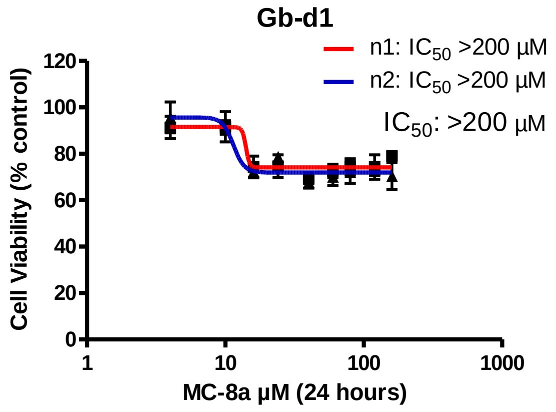

Hela

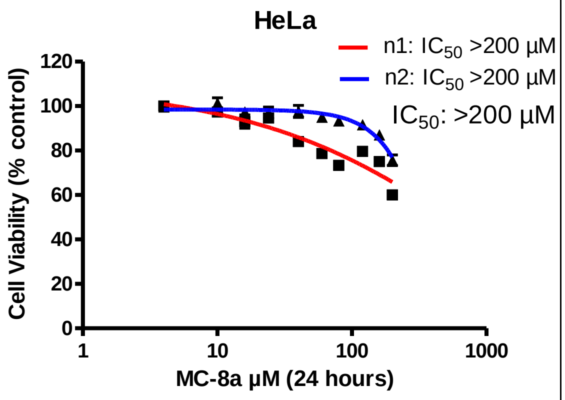

G-415

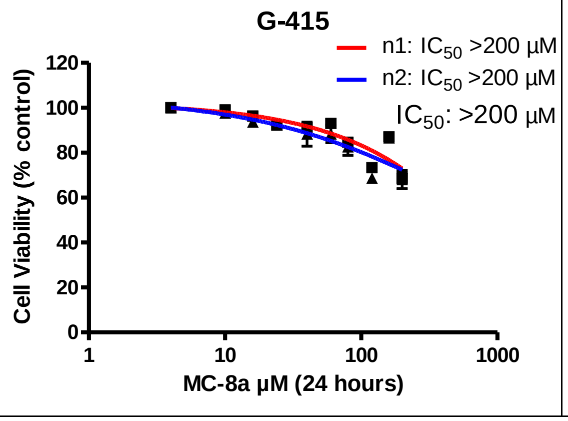

Figure S19: Cytotoxicity curves of compound 4s

Compound 4t

U937

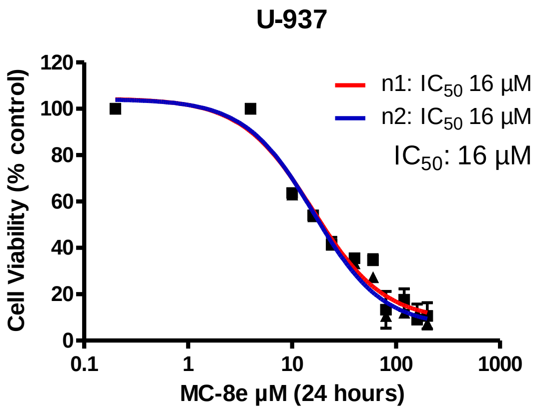

T98G

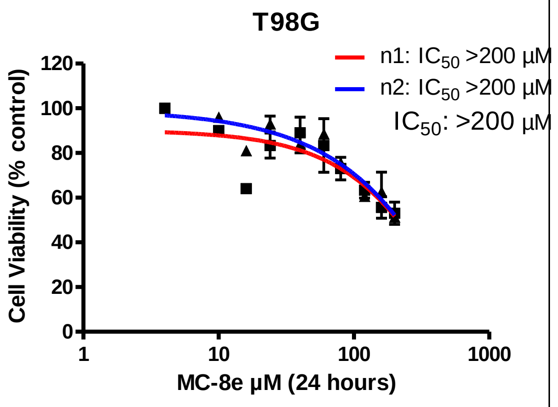

Gb-d1

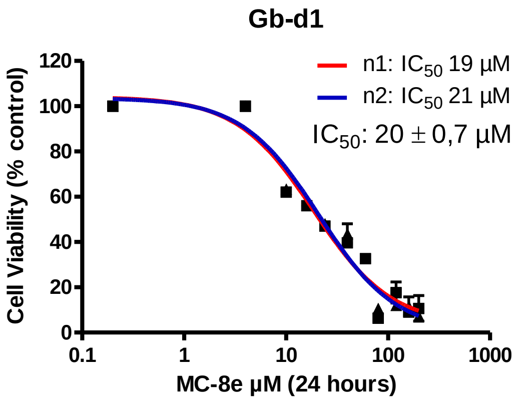

Hela

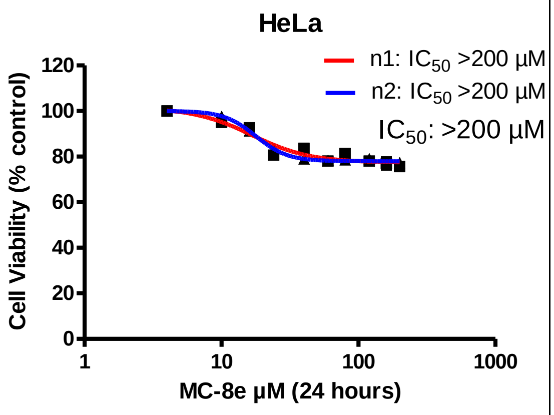

G-415

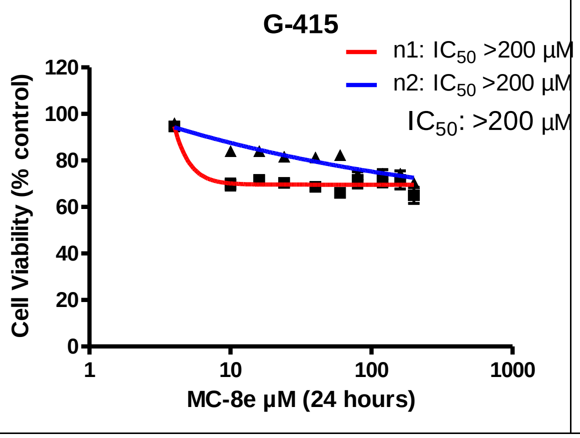

Figure S20: Cytotoxicity curves of compound 4t

Compound 4v

U937	T98G
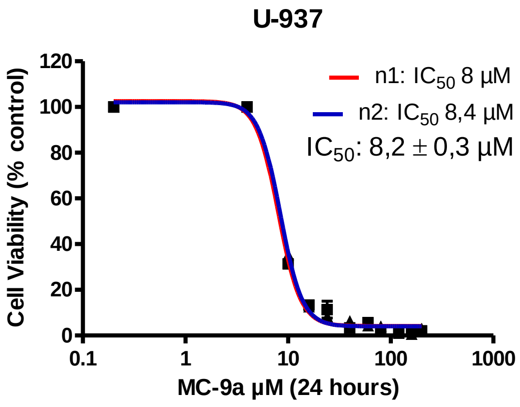	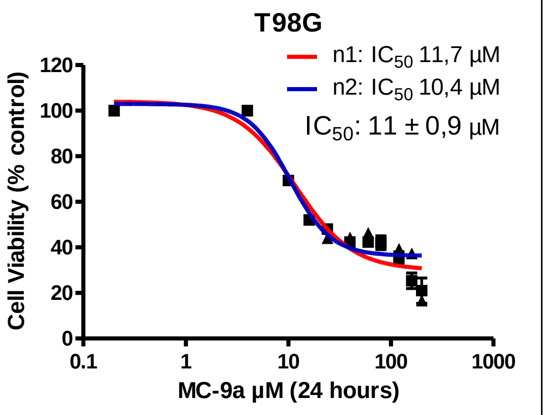
**Gb-d1**	**Hela**
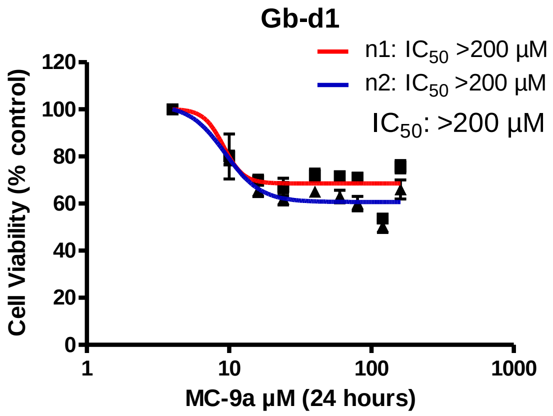	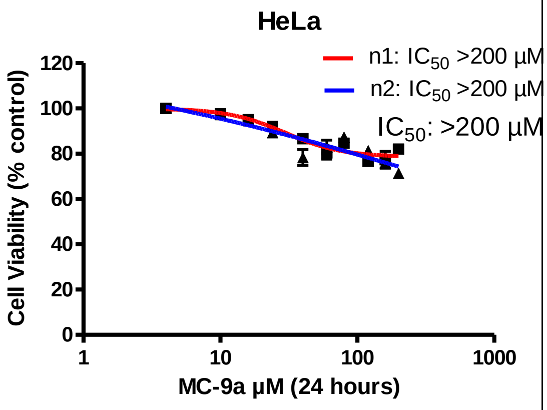
**G-415**	
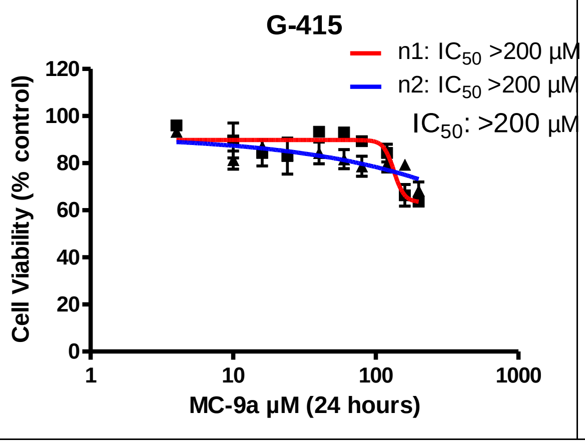	

Figure S21: Cytotoxicity curves of compound 4v

4. ^{1}H NMR, ^{13}C NMR/APT, HSQC, HMBC spectra of compounds.

Compound 2a

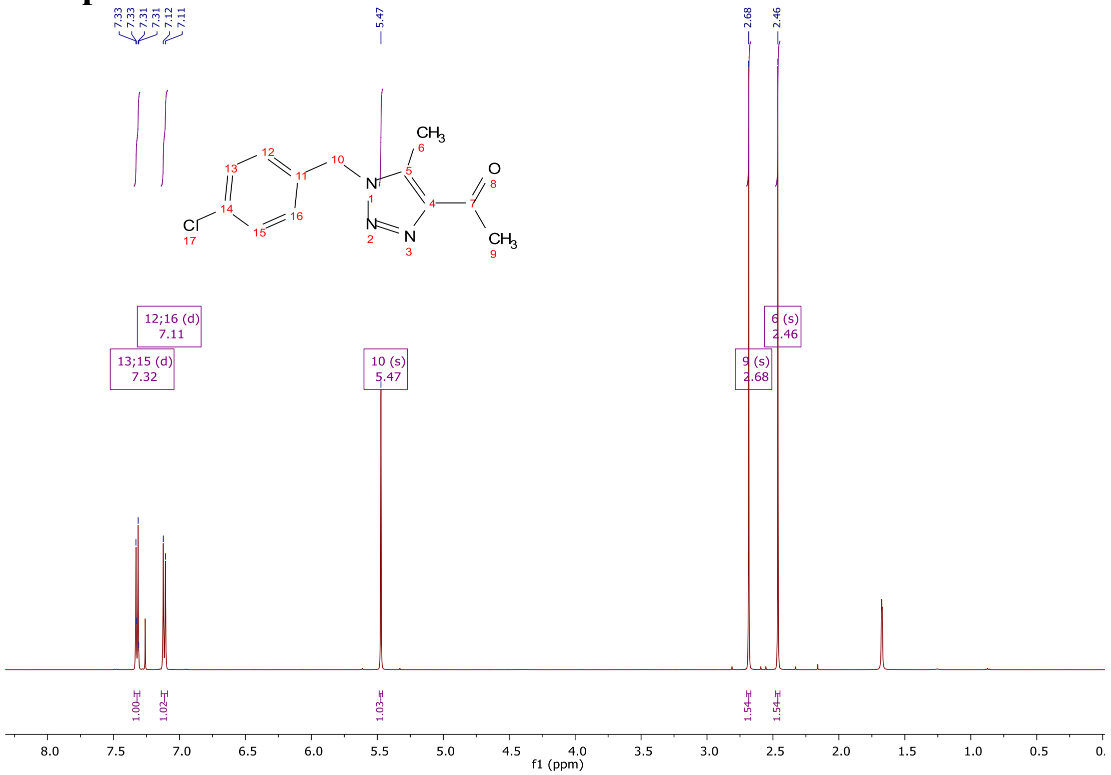

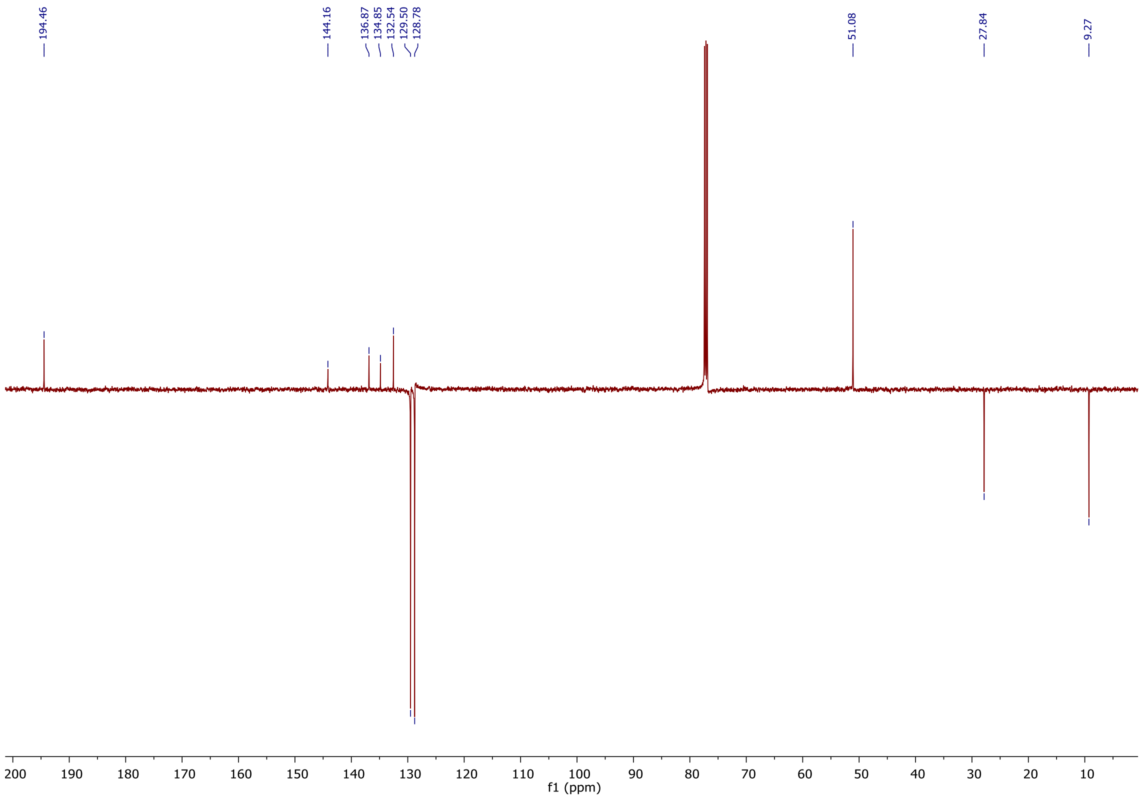

Compound 2b

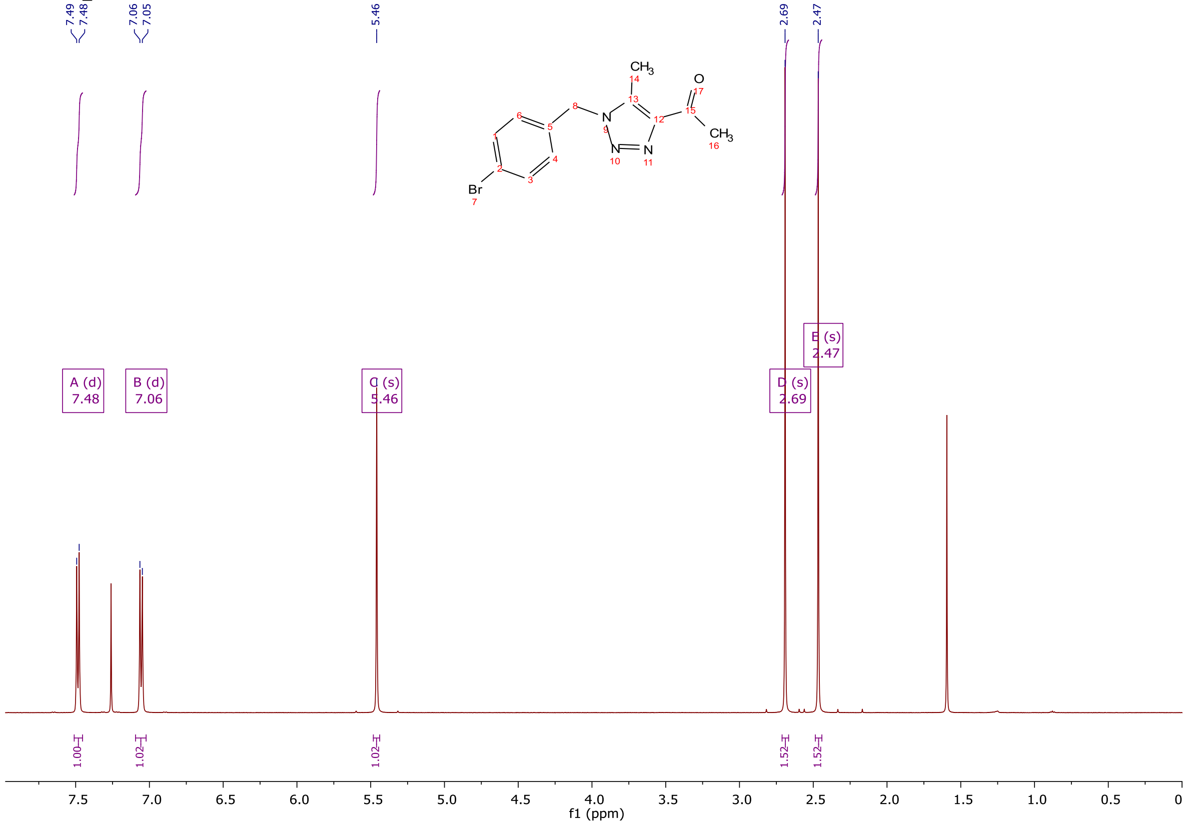

7.49
7.48
7.06
7.05
5.46
2.69
2.47
CH3
O
N
N
N
CH3
Br
A (d) 7.48
B (d) 7.06
C (s) 5.46
D (s) 2.69
E (s) 2.47
1.00
1.02
1.02
1.52
1.52
7.5 7.0 6.5 6.0 5.5 5.0 4.5 4.0 3.5 3.0 2.5 2.0 1.5 1.0 0.5 0
f1 (ppm)

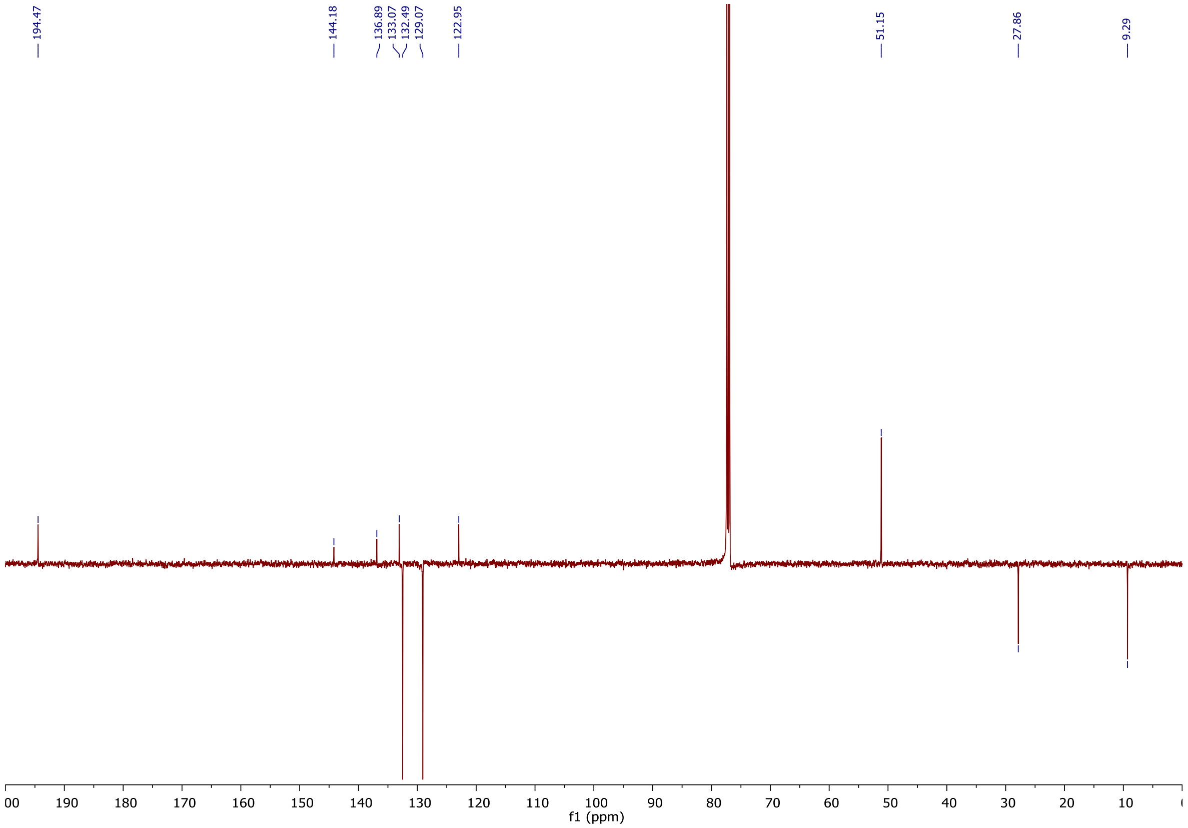

194.47
144.18
136.89
133.07
132.49
129.07
122.95
51.15
27.86
9.29
190 180 170 160 150 140 130 120 110 100 90 80 70 60 50 40 30 20 10
f1 (ppm)

Compound 2c

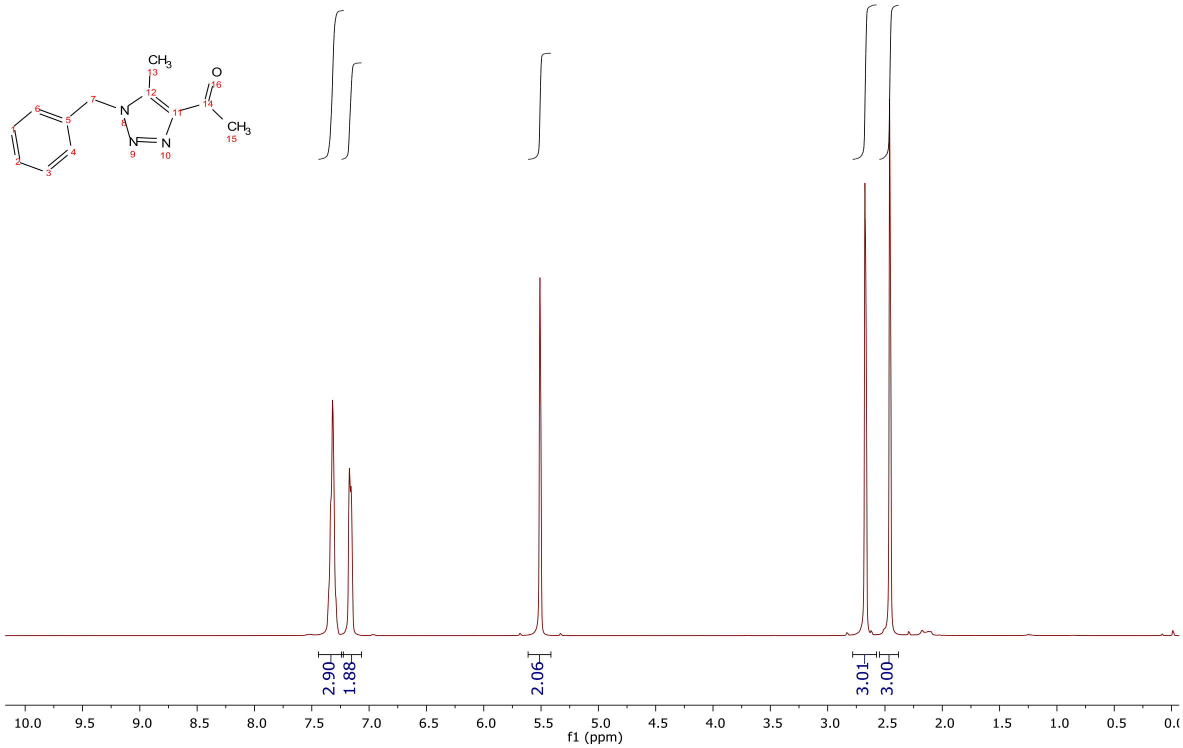
CH3
O
N
N
N
CH3
2.90
1.88
2.06
3.01
3.00
f1 (ppm)

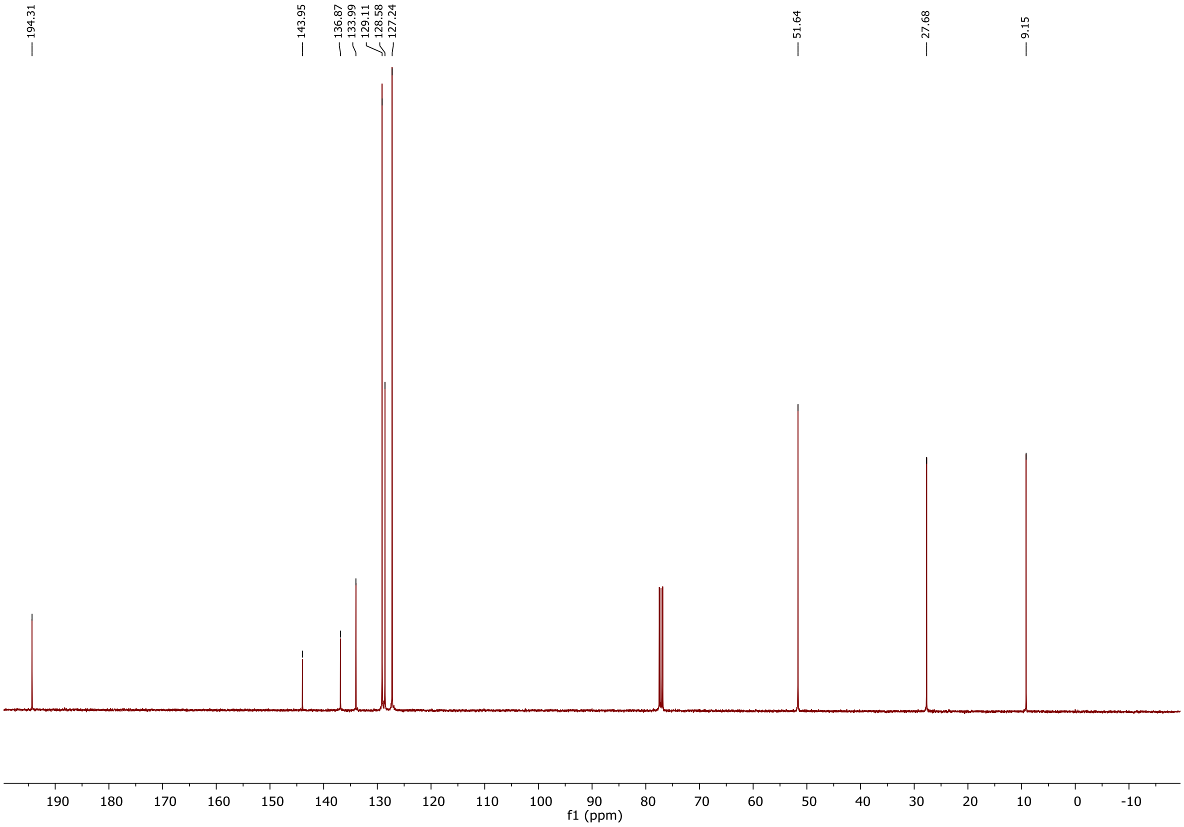
194.31
143.95
136.87
133.99
129.11
128.58
127.24
51.64
27.68
9.15
f1 (ppm)

Compound 2d

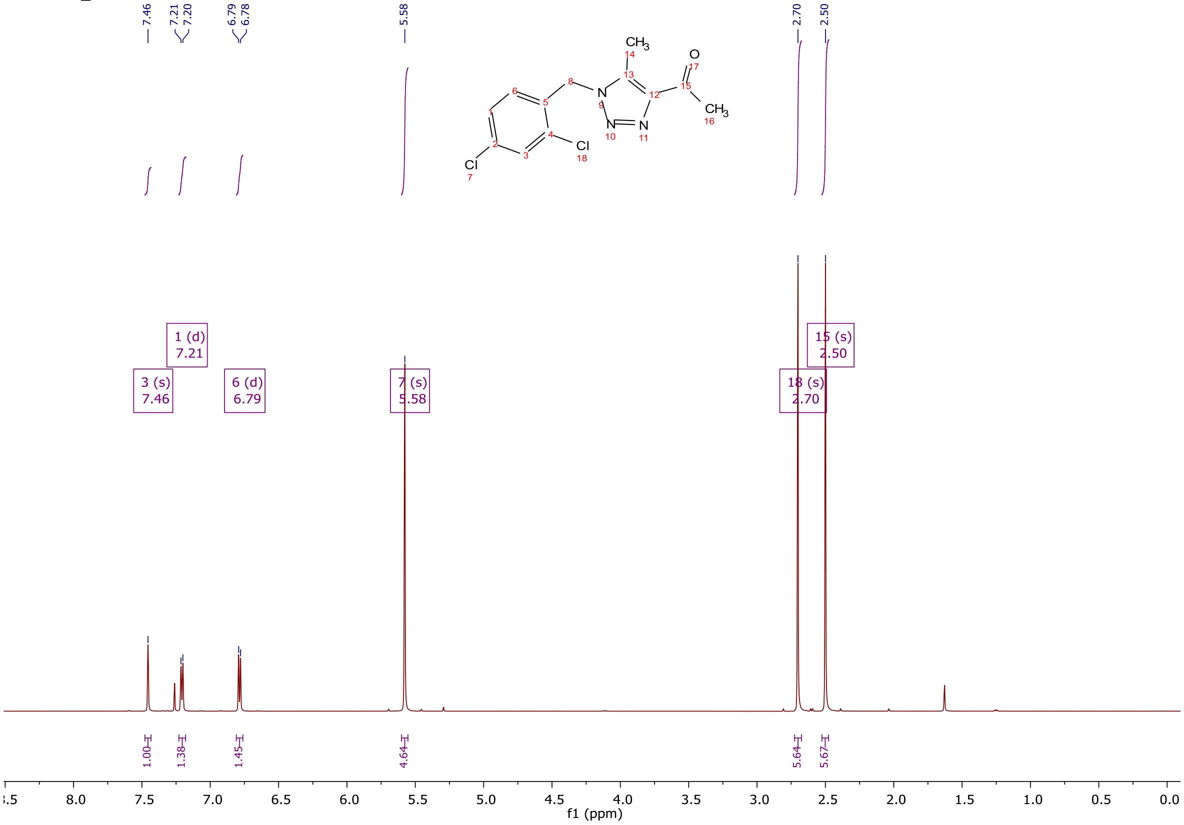
7.46
7.21
7.20
6.79
6.78
5.58
2.70
2.50
CH3
O
N
N
N
CH3
Cl
Cl
1 (d)
7.21
3 (s)
7.46
6 (d)
6.79
7 (s)
5.58
18 (s)
2.70
15 (s)
2.50
1.00
1.38
1.45
4.64
5.64
5.67
f1 (ppm)

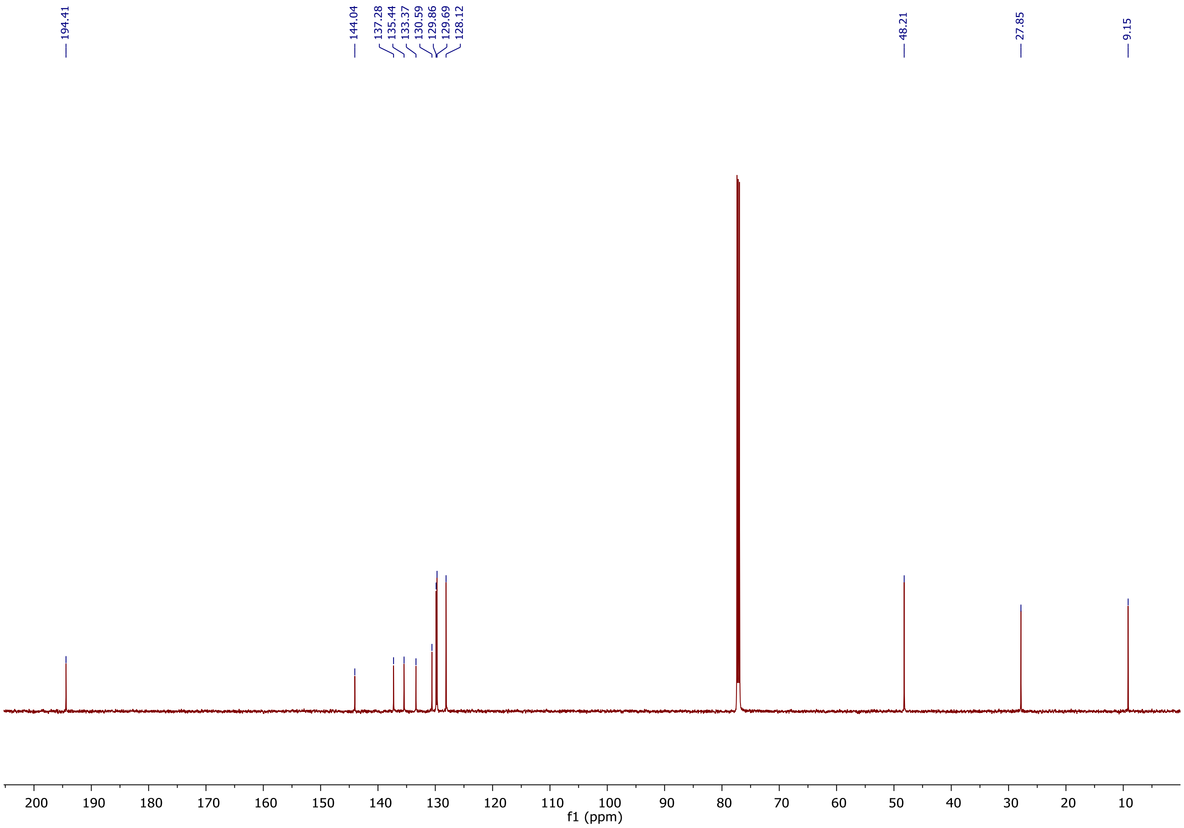
194.41
144.04
137.28
135.44
133.37
130.59
129.86
129.69
128.12
48.21
27.85
9.15
f1 (ppm)

Compound 4a

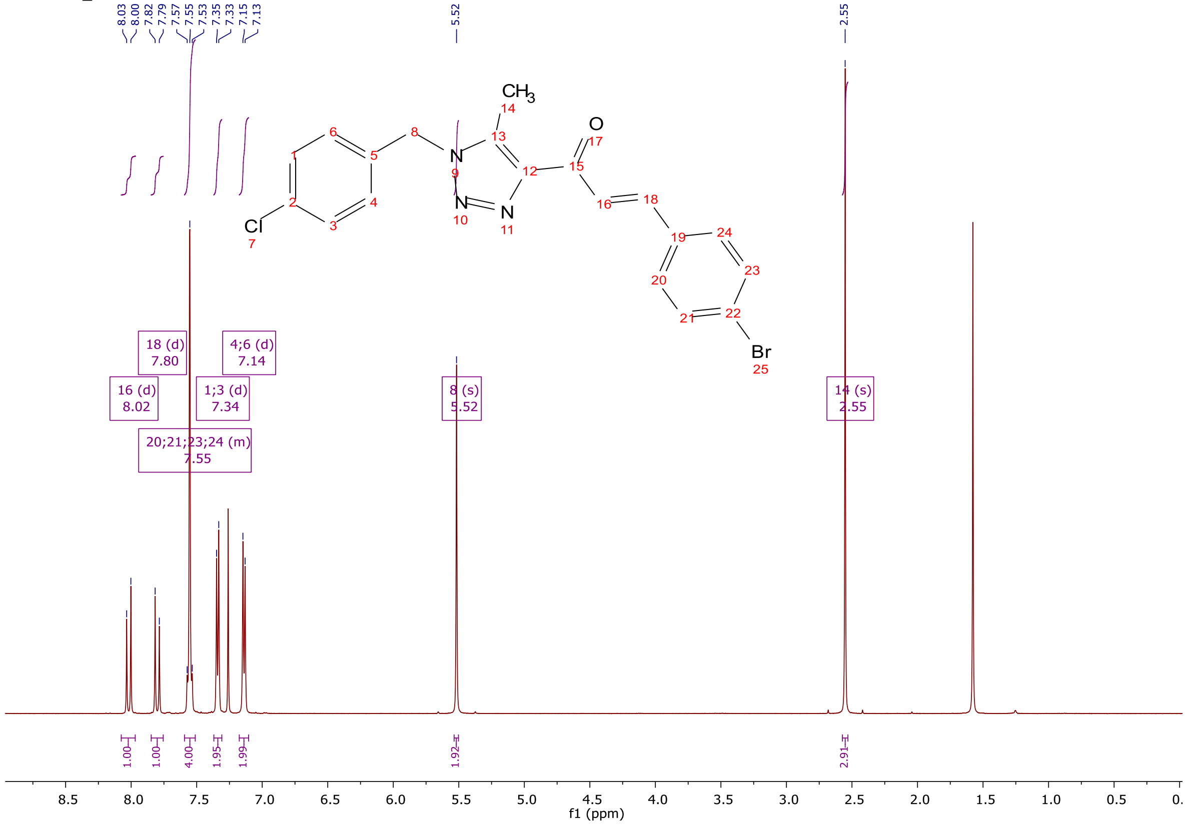
8.03
8.00
7.82
7.79
7.57
7.55
7.53
7.35
7.33
7.15
7.13
5.52
2.55
CH3
O
N
N
N
Cl
Br
18 (d)
7.80
4;6 (d)
7.14
16 (d)
8.02
1;3 (d)
7.34
8 (s)
5.52
14 (s)
2.55
20;21;23;24 (m)
7.55
1.00
1.00
4.00
1.95
1.99
1.92
2.91
f1 (ppm)

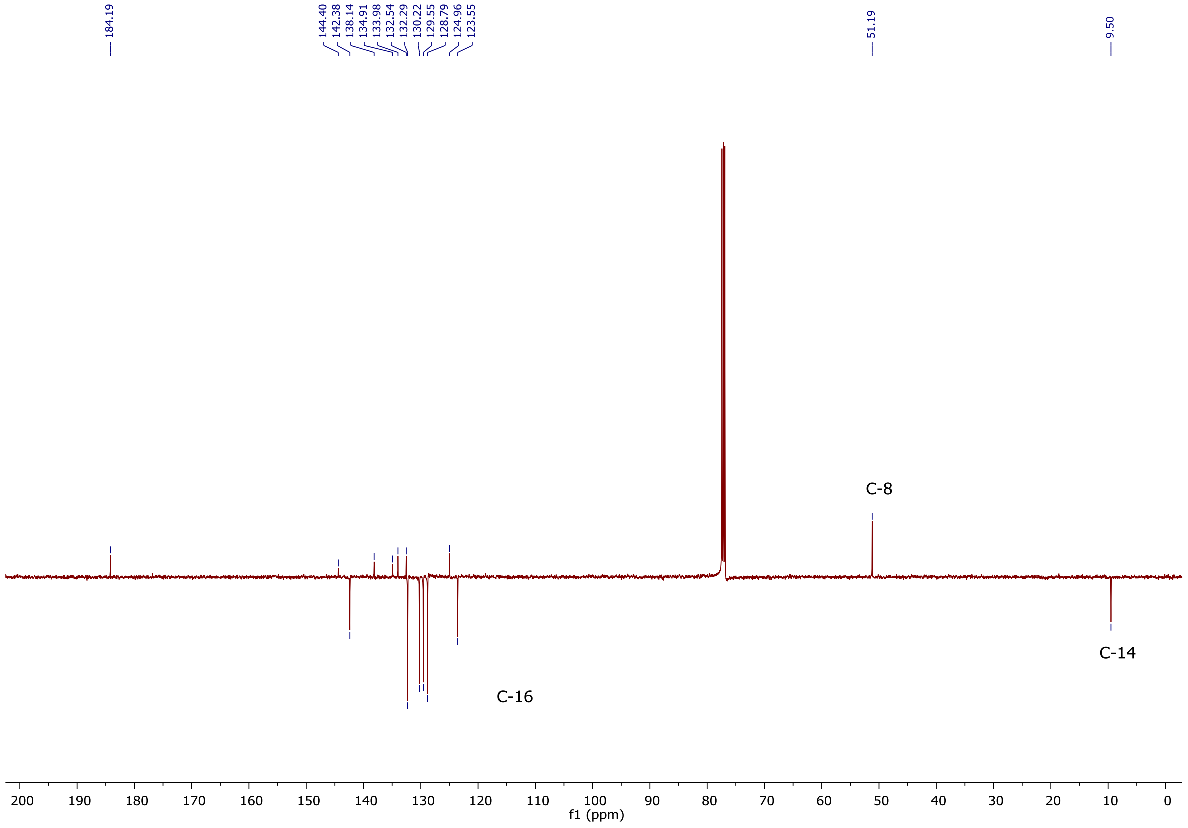
184.19
144.40
142.38
138.14
134.91
133.98
132.54
132.29
130.22
129.55
128.79
124.96
123.55
51.19
9.50
C-8
C-16
C-14
f1 (ppm)

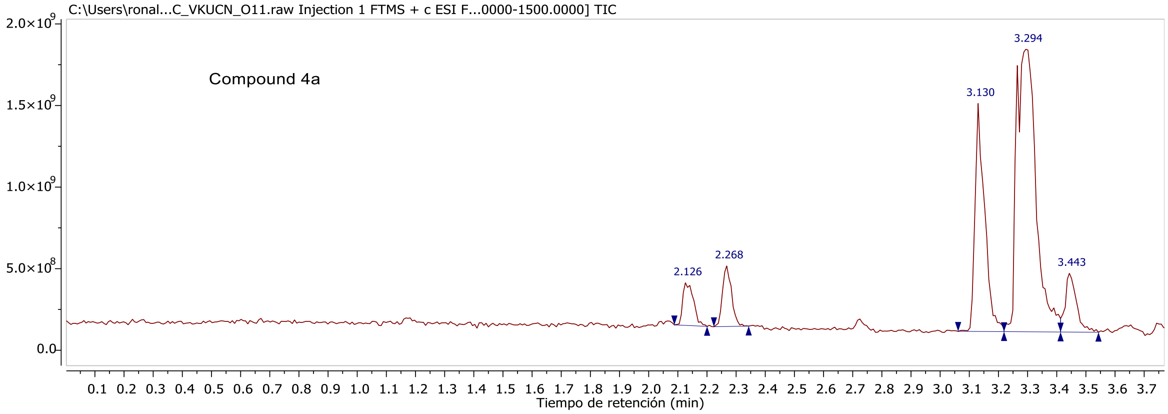
C:\Users\ronal...C_VKUCN_O11.raw Injection 1 FTMS + c ESI F...0000-1500.0000] TIC
Compound 4a
2.126
2.268
3.130
3.294
3.443
Tiempo de retención (min)

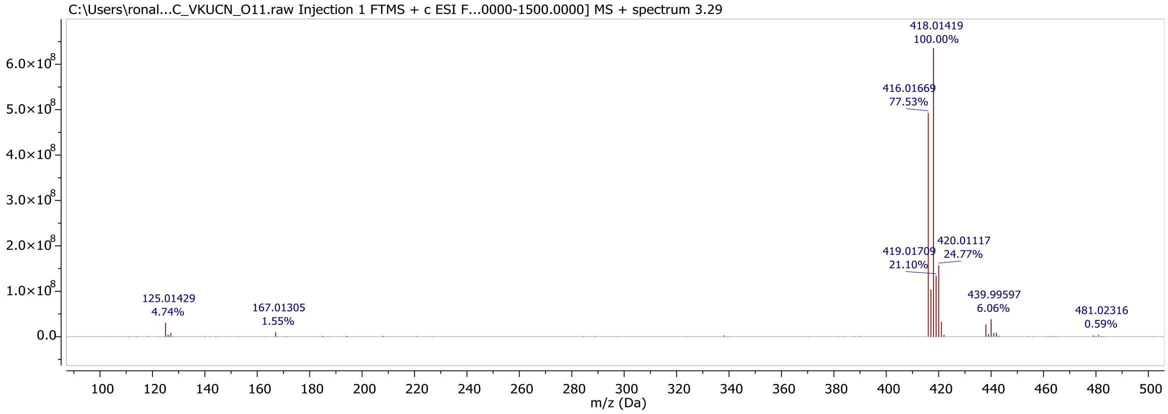
C:\Users\ronal...C_VKUCN_O11.raw Injection 1 FTMS + c ESI F...0000-1500.0000] MS + spectrum 3.29
418.01419
100.00%
416.01669
77.53%
420.01117
24.77%
419.01709
21.10%
125.01429
4.74%
167.01305
1.55%
439.99597
6.06%
481.02316
0.59%
m/z (Da)

Compound 4b

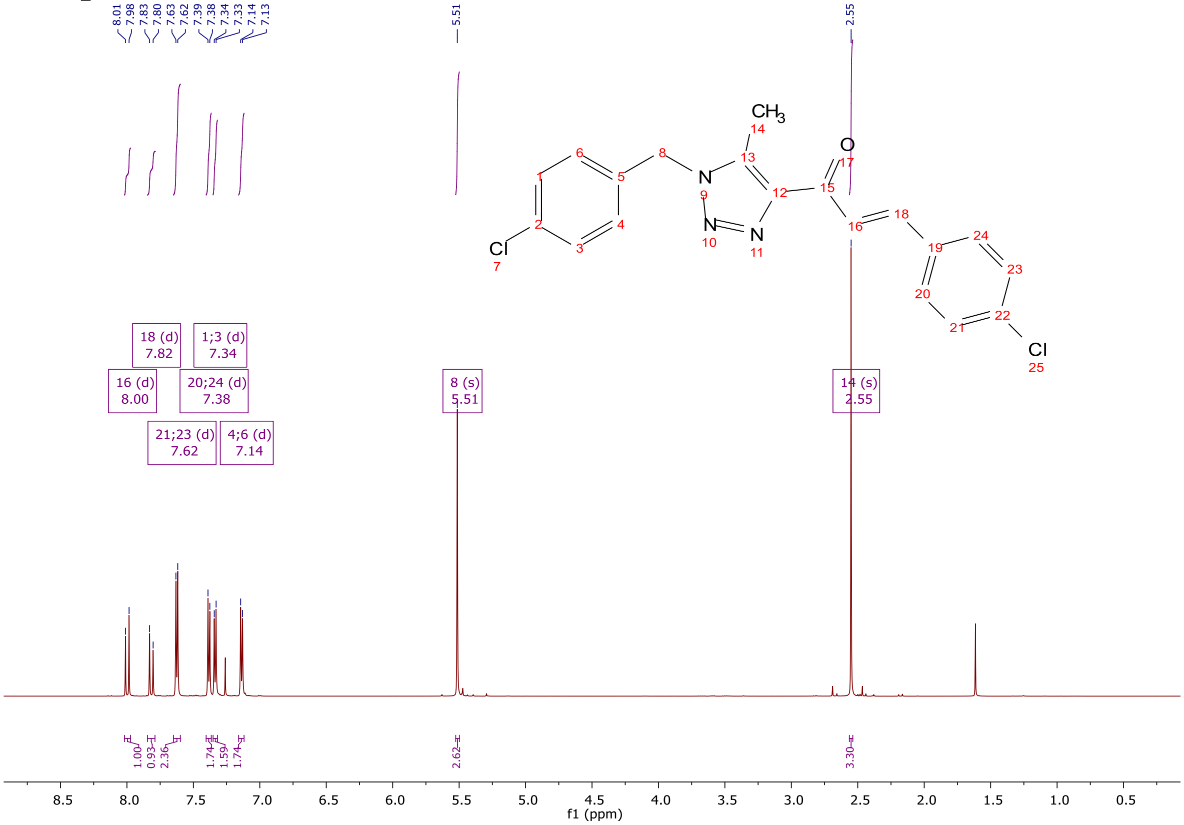

8.01
7.98
7.83
7.80
7.63
7.62
7.39
7.38
7.34
7.33
7.14
7.13
5.51
2.55
18 (d) 7.82
1;3 (d) 7.34
16 (d) 8.00
20;24 (d) 7.38
8 (s) 5.51
14 (s) 2.55
21;23 (d) 7.62
4;6 (d) 7.14
1.00
0.93
2.36
1.74
1.59
1.74
2.62
3.30
f1 (ppm)

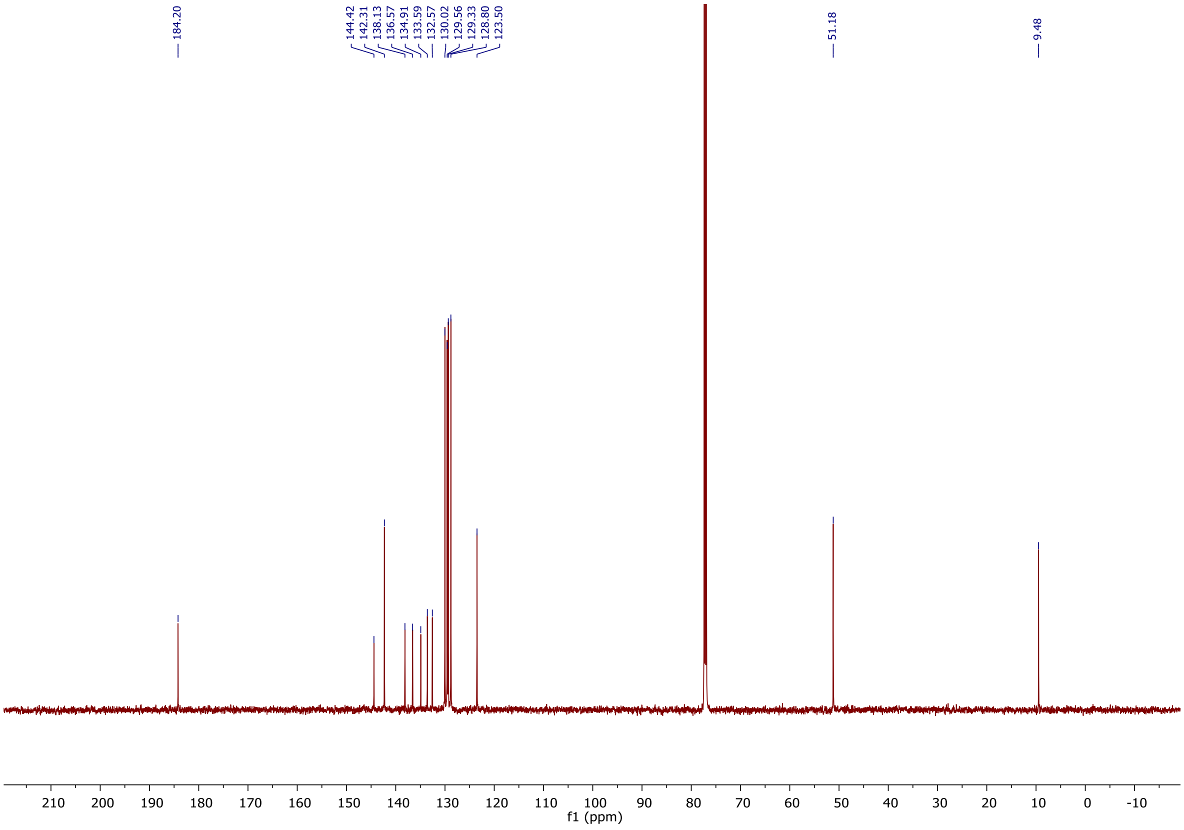

184.20
144.42
142.31
138.13
136.57
134.91
133.59
132.57
130.02
129.56
129.33
128.80
123.50
51.18
9.48
f1 (ppm)

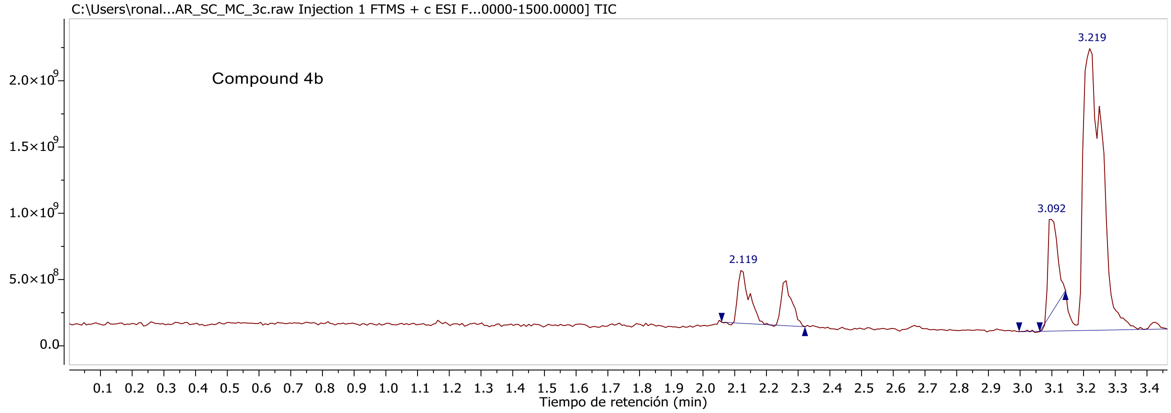
C:\Users\ronal...AR_SC_MC_3c.raw Injection 1 FTMS + c ESI F...0000-1500.0000] TIC
Compound 4b
3.219
3.092
2.119
Tiempo de retención (min)

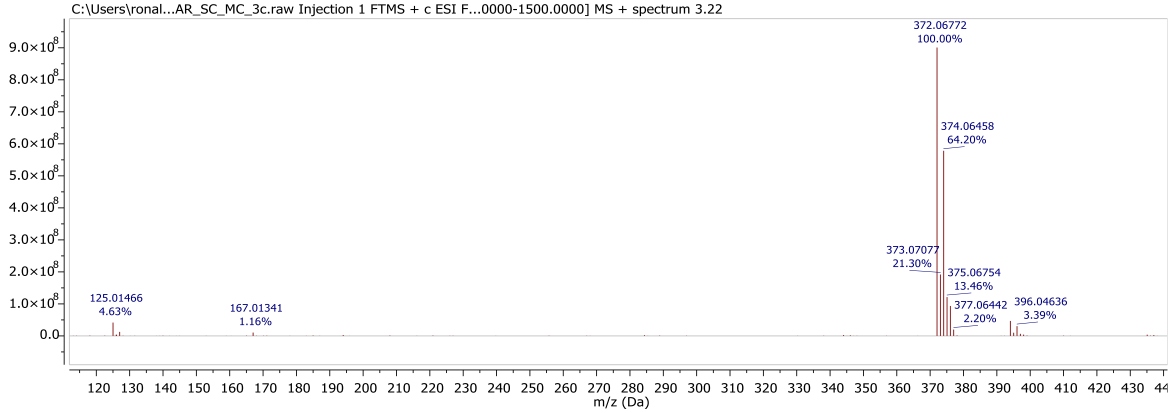
C:\Users\ronal...AR_SC_MC_3c.raw Injection 1 FTMS + c ESI F...0000-1500.0000] MS + spectrum 3.22
372.06772
100.00%
374.06458
64.20%
373.07077
21.30%
375.06754
13.46%
377.06442
2.20%
396.04636
3.39%
125.01466
4.63%
167.01341
1.16%
m/z (Da)

Compound 4c

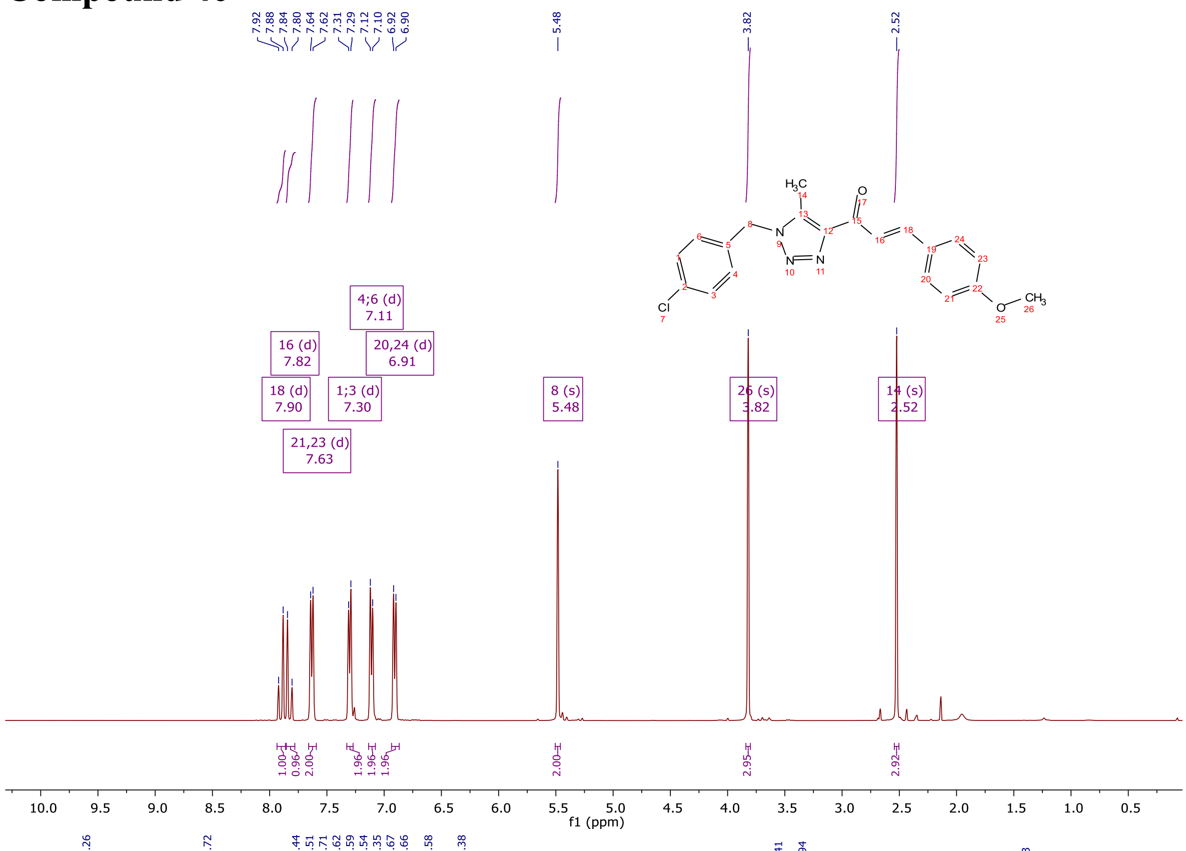
7.92
7.88
7.84
7.80
7.64
7.62
7.31
7.29
7.12
7.10
6.92
6.90
5.48
3.82
2.52
4;6 (d)
7.11
16 (d)
7.82
20,24 (d)
6.91
18 (d)
7.90
1;3 (d)
7.30
8 (s)
5.48
26 (s)
3.82
14 (s)
2.52
21,23 (d)
7.63
1.00
0.96
2.00
1.96
1.96
1.96
2.00
2.95
2.92
f1 (ppm)

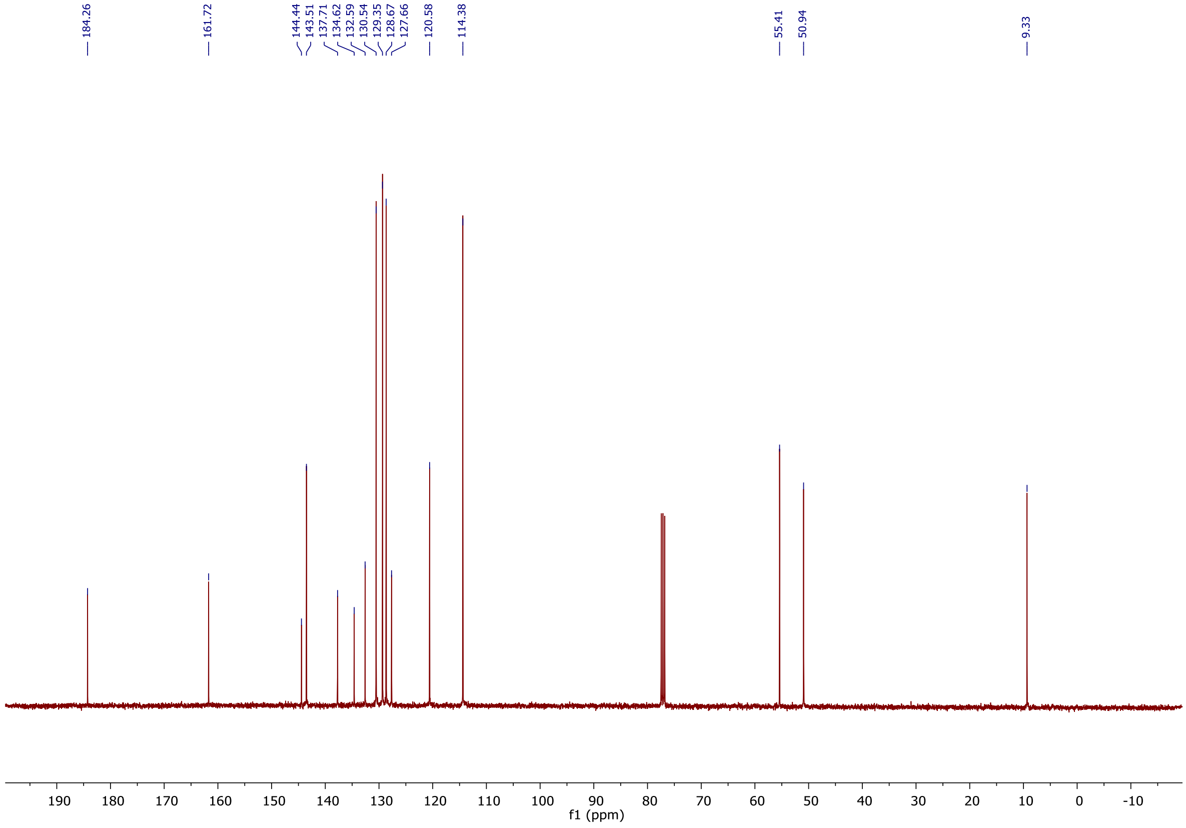
184.26
161.72
144.44
143.51
137.71
134.62
132.59
130.54
129.35
128.67
127.66
120.58
114.38
55.41
50.94
9.33
f1 (ppm)

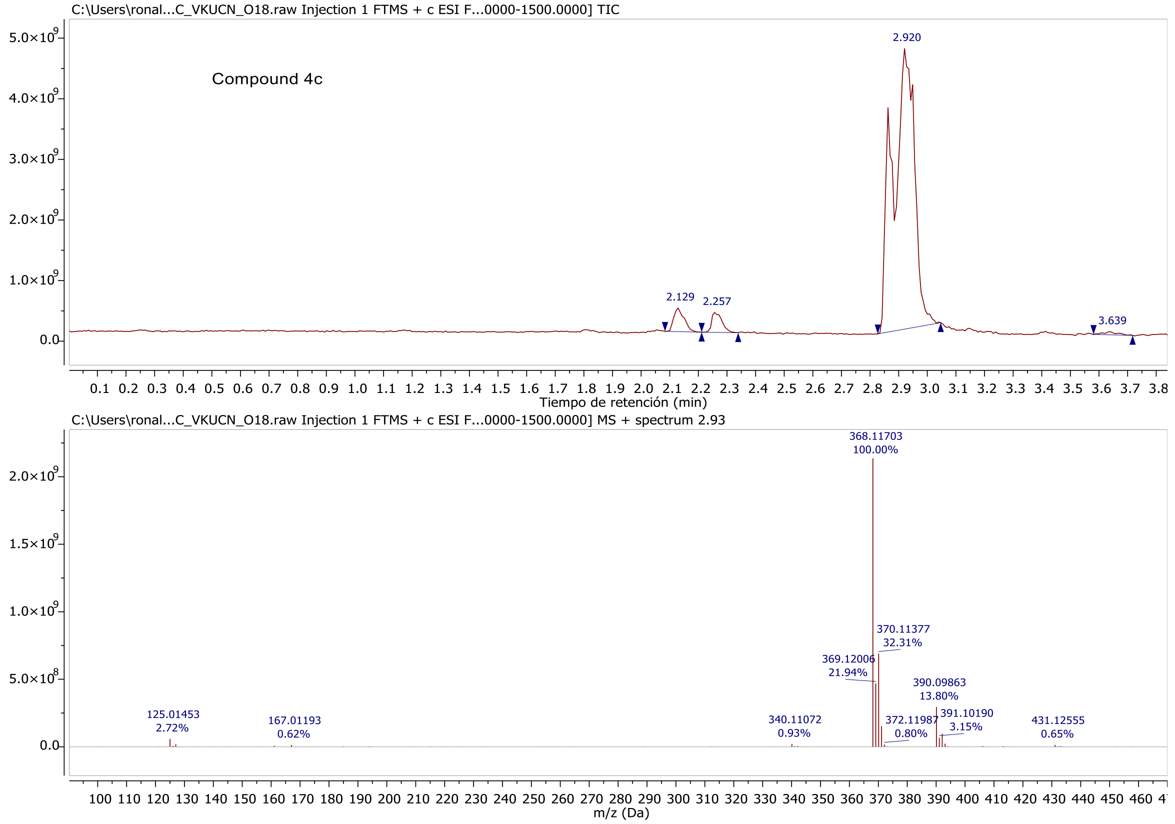

C:\Users\ronal...C_VKUCN_O18.raw Injection 1 FTMS + c ESI F...0000-1500.0000] TIC
Compound 4c
2.920
2.129
2.257
3.639
Tiempo de retención (min)
C:\Users\ronal...C_VKUCN_O18.raw Injection 1 FTMS + c ESI F...0000-1500.0000] MS + spectrum 2.93
368.11703
100.00%
370.11377
32.31%
369.12006
21.94%
390.09863
13.80%
125.01453
2.72%
167.01193
0.62%
340.11072
0.93%
372.11987
0.80%
391.10190
3.15%
431.12555
0.65%
m/z (Da)

Compound 4d

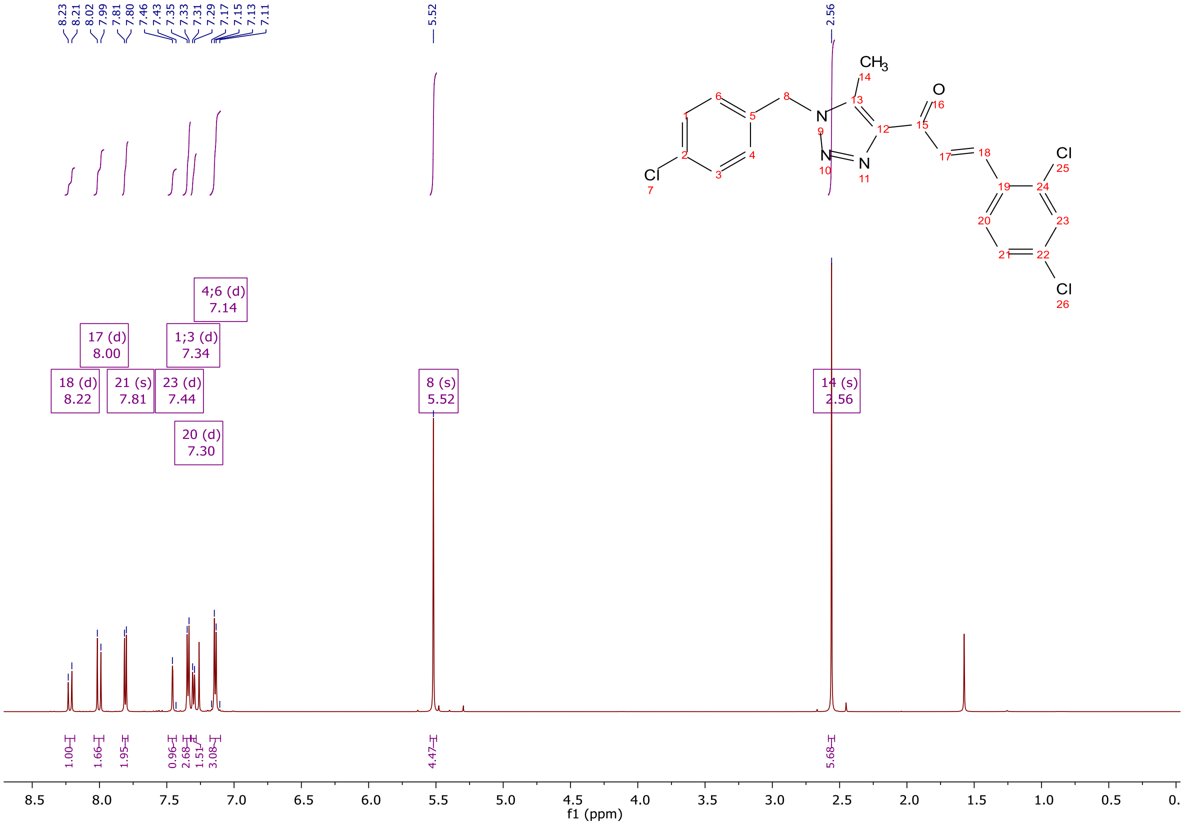

8.23
8.21
8.02
7.99
7.81
7.80
7.46
7.43
7.35
7.33
7.31
7.29
7.17
7.15
7.13
7.11
5.52
2.56
4;6 (d)
7.14
17 (d)
8.00
1;3 (d)
7.34
18 (d)
8.22
21 (s)
7.81
23 (d)
7.44
20 (d)
7.30
8 (s)
5.52
14 (s)
2.56
1.00
1.66
1.95
0.96
2.68
1.51
3.08
4.47
5.68
f1 (ppm)

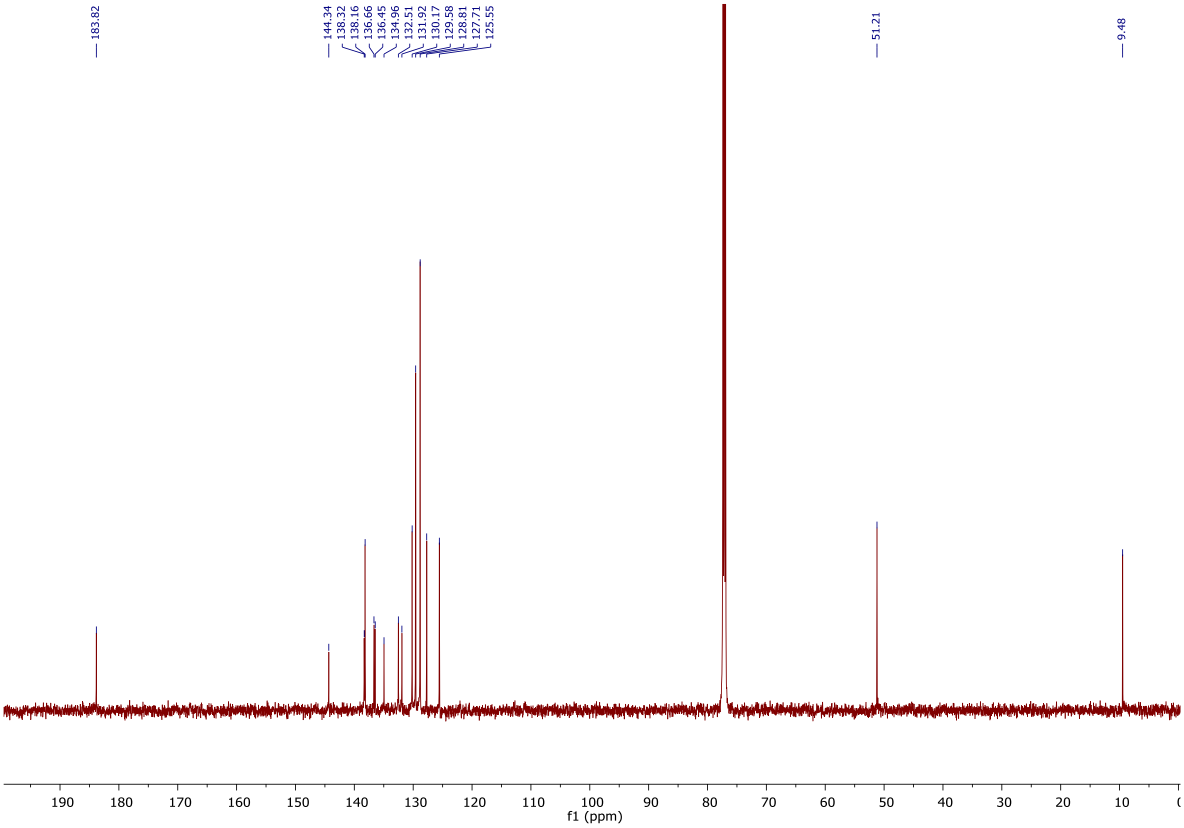

183.82
144.34
138.32
138.16
136.66
136.45
134.96
132.51
131.92
130.17
129.58
128.81
127.71
125.55
51.21
9.48
f1 (ppm)

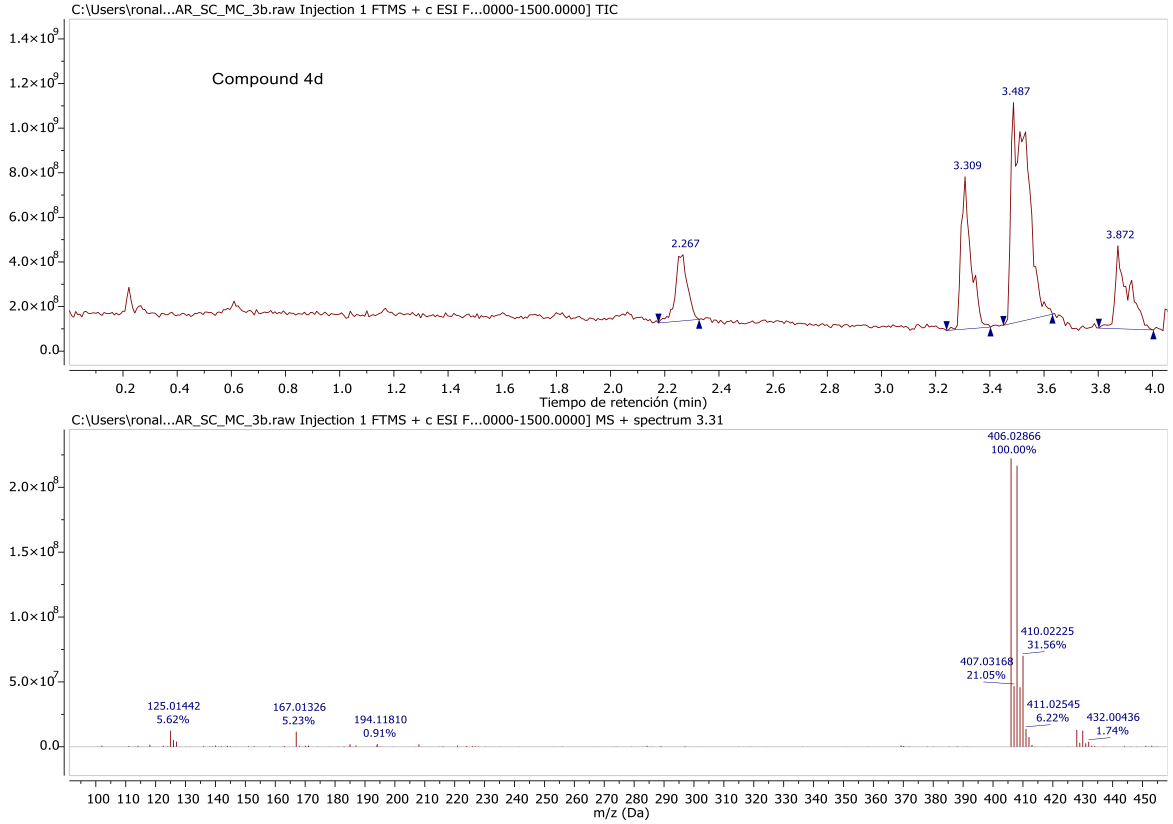

C:\Users\ronal...AR_SC_MC_3b.raw Injection 1 FTMS + c ESI F...0000-1500.0000] TIC
Compound 4d
2.267
3.309
3.487
3.872
Tiempo de retención (min)
C:\Users\ronal...AR_SC_MC_3b.raw Injection 1 FTMS + c ESI F...0000-1500.0000] MS + spectrum 3.31
406.02866
100.00%
410.02225
31.56%
407.03168
21.05%
411.02545
6.22%
432.00436
1.74%
125.01442
5.62%
167.01326
5.23%
194.11810
0.91%
m/z (Da)

Compound 4e

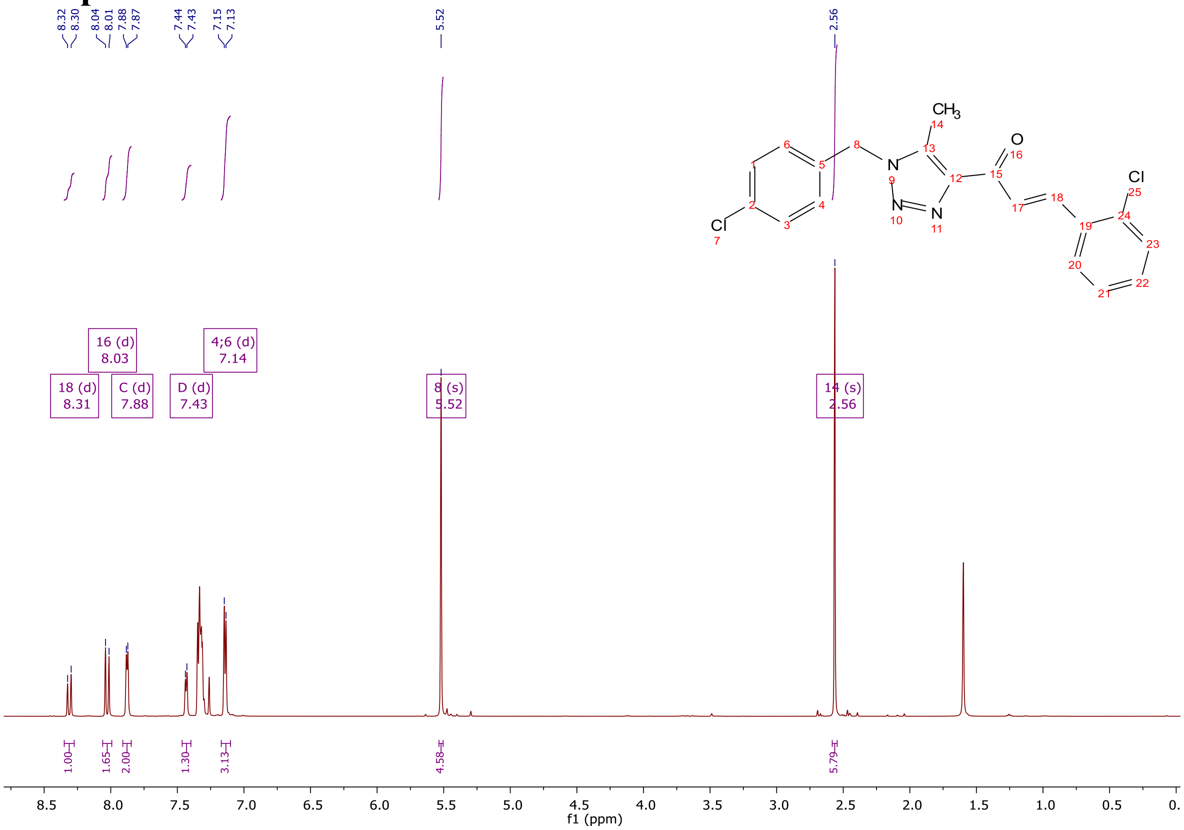

8.32
8.30
8.04
8.01
7.88
7.87
7.44
7.43
7.15
7.13
5.52
2.56
CH3
O
Cl
Cl
N
N
N
16 (d)
8.03
4;6 (d)
7.14
18 (d)
8.31
C (d)
7.88
D (d)
7.43
8 (s)
5.52
14 (s)
2.56
1.00
1.65
2.00
1.30
3.13
4.58
5.79
f1 (ppm)

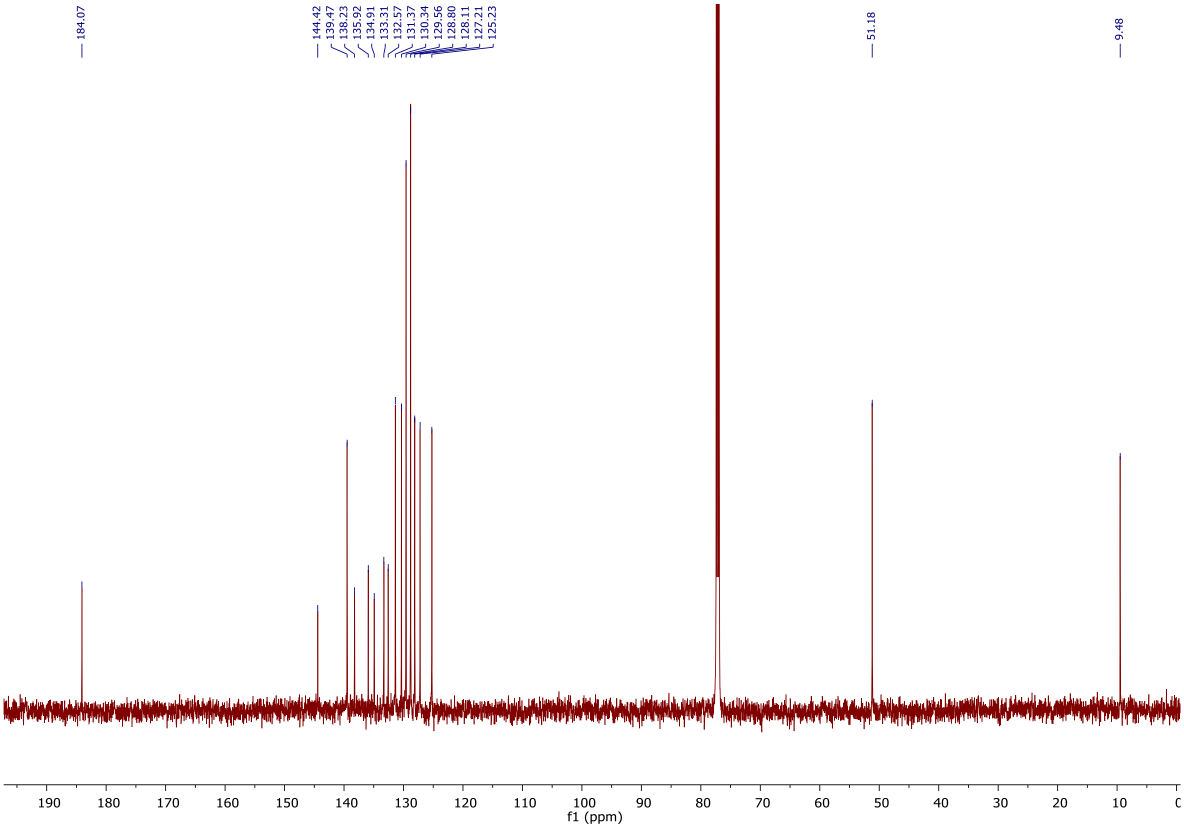

184.07
144.42
139.47
138.23
135.92
134.91
133.31
132.57
131.37
130.34
129.56
128.80
128.11
127.21
125.23
51.18
9.48
f1 (ppm)

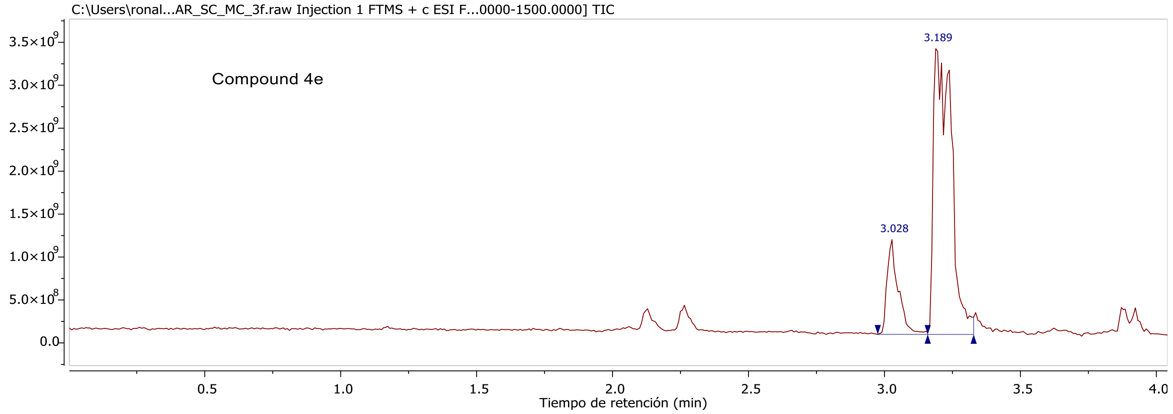
C:\Users\ronal...AR_SC_MC_3f.raw Injection 1 FTMS + c ESI F...0000-1500.0000] TIC
Compound 4e
3.189
3.028
Tiempo de retención (min)

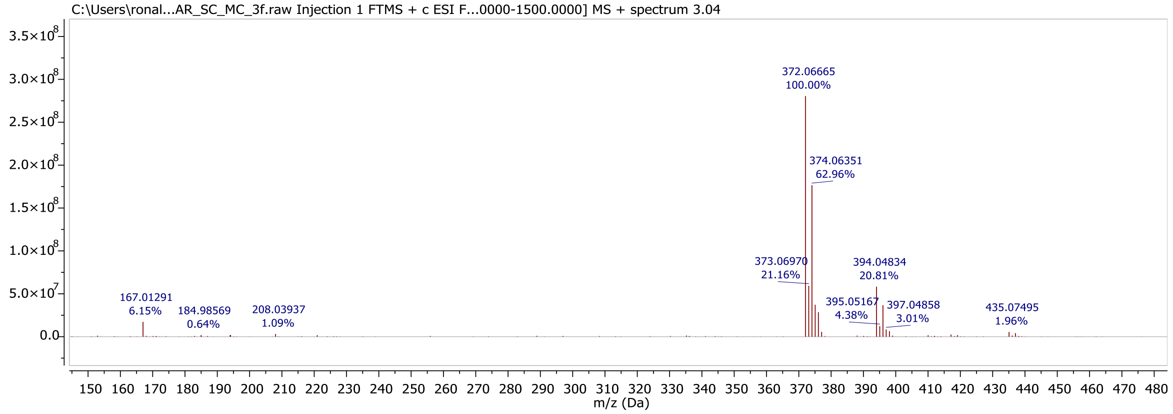
C:\Users\ronal...AR_SC_MC_3f.raw Injection 1 FTMS + c ESI F...0000-1500.0000] MS + spectrum 3.04
372.06665
100.00%
374.06351
62.96%
373.06970
21.16%
394.04834
20.81%
395.05167
4.38%
397.04858
3.01%
435.07495
1.96%
167.01291
6.15%
184.98569
0.64%
208.03937
1.09%
m/z (Da)

Compound 4f

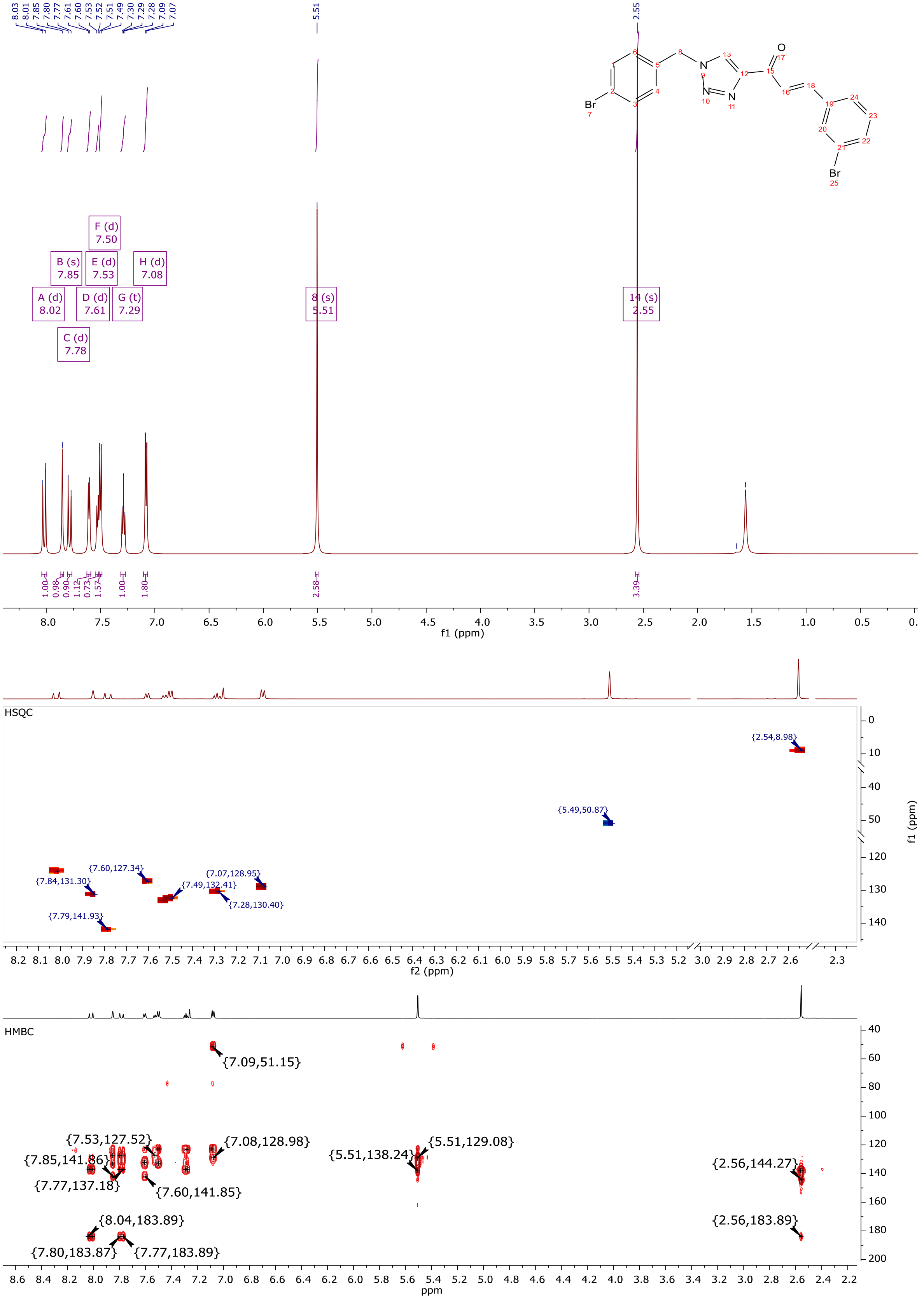

A (d) 8.02
B (s) 7.85
C (d) 7.78
D (d) 7.61
E (d) 7.53
F (d) 7.50
G (t) 7.29
H (d) 7.08
8 (s) 5.51
14 (s) 2.55
f1 (ppm)
HSQC
{2.54,8.98}
{5.49,50.87}
{7.60,127.34}
{7.84,131.30}
{7.07,128.95}
{7.49,132.41}
{7.28,130.40}
{7.79,141.93}
f2 (ppm)
HMBC
{7.09,51.15}
{7.53,127.52}
{7.08,128.98}
{7.85,141.86}
{7.77,137.18}
{7.60,141.85}
{5.51,138.24}
{5.51,129.08}
{2.56,144.27}
{8.04,183.89}
{2.56,183.89}
{7.80,183.87}
{7.77,183.89}
ppm

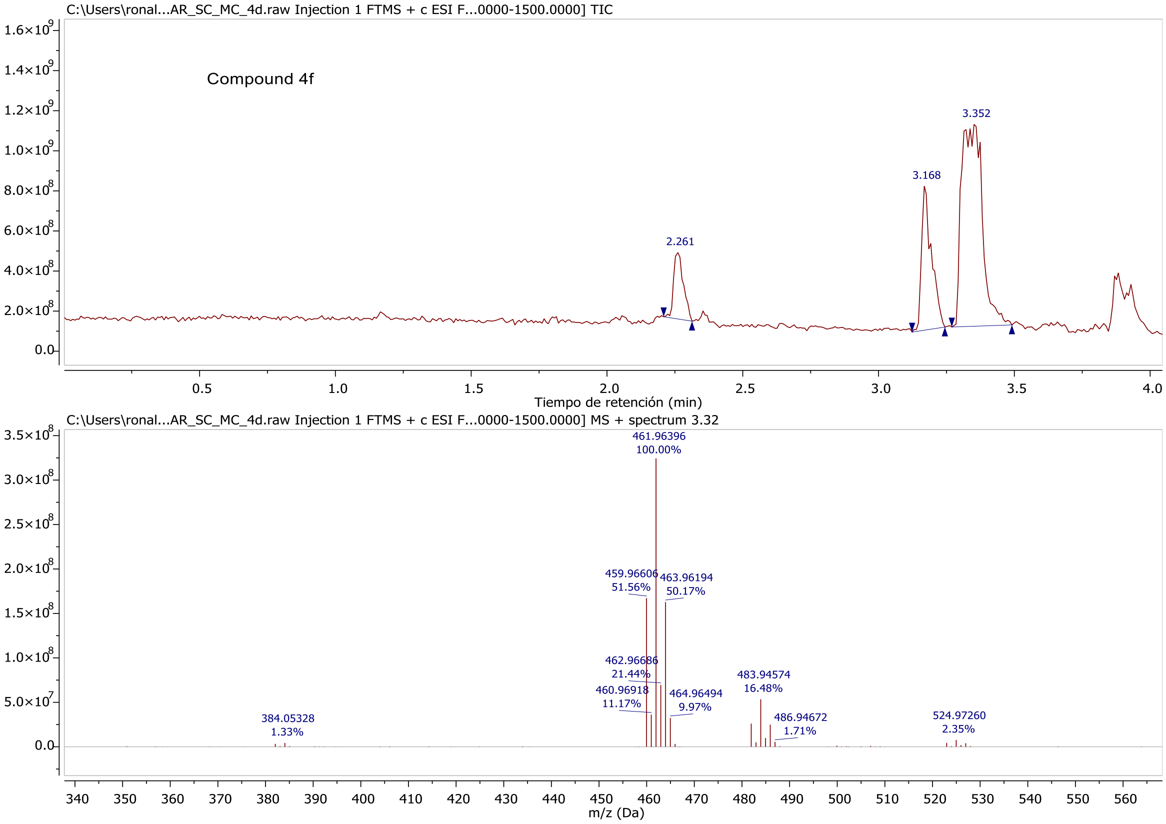
C:\Users\ronal...AR_SC_MC_4d.raw Injection 1 FTMS + c ESI F...0000-1500.0000] TIC
Compound 4f
2.261
3.168
3.352
Tiempo de retención (min)
C:\Users\ronal...AR_SC_MC_4d.raw Injection 1 FTMS + c ESI F...0000-1500.0000] MS + spectrum 3.32
461.96396 100.00%
459.96606 51.56%
463.96194 50.17%
462.96686 21.44%
460.96918 11.17%
464.96494 9.97%
483.94574 16.48%
486.94672 1.71%
384.05328 1.33%
524.97260 2.35%
m/z (Da)

Compound 4g

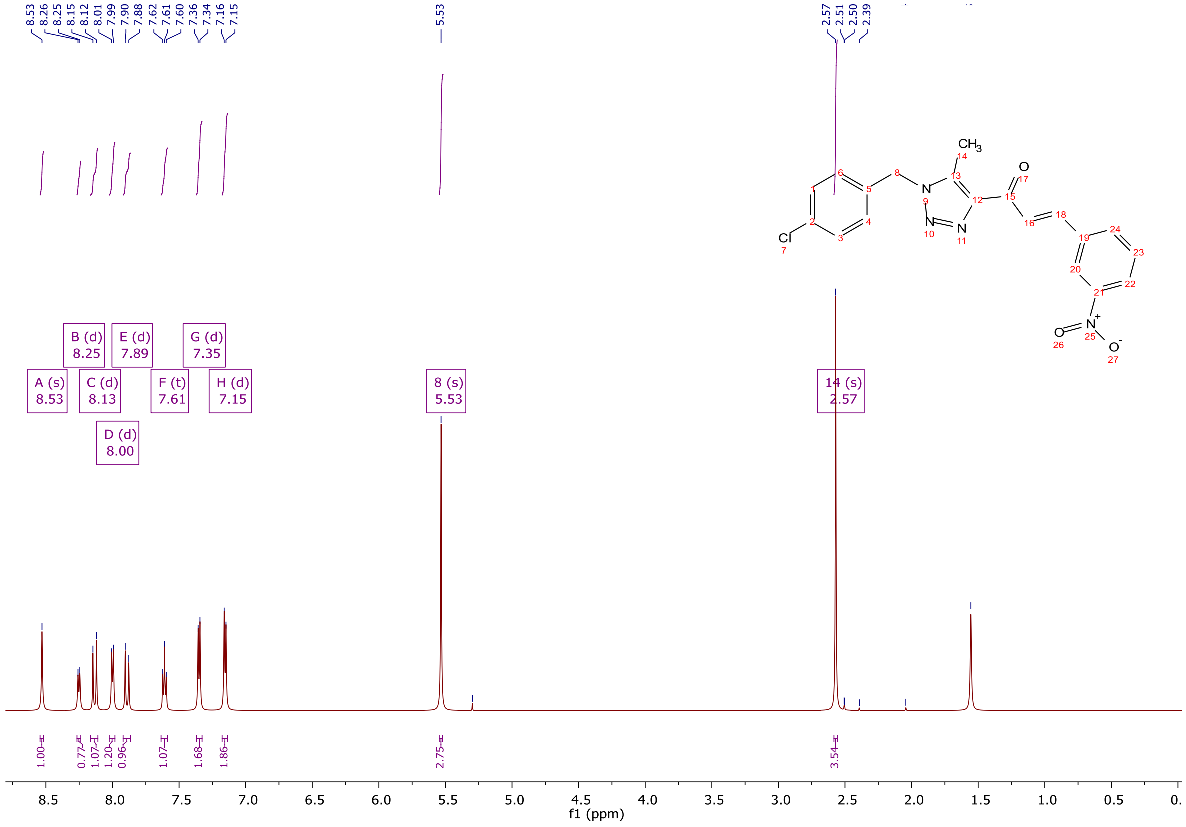

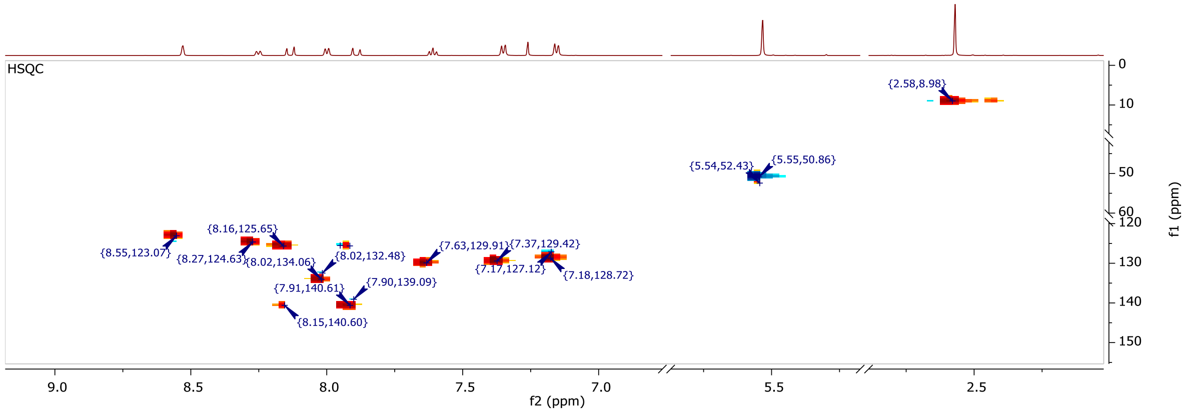

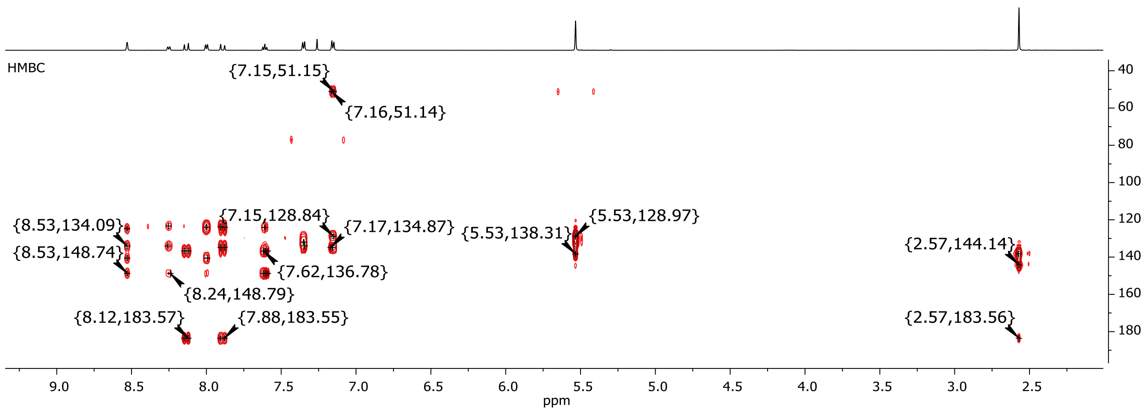

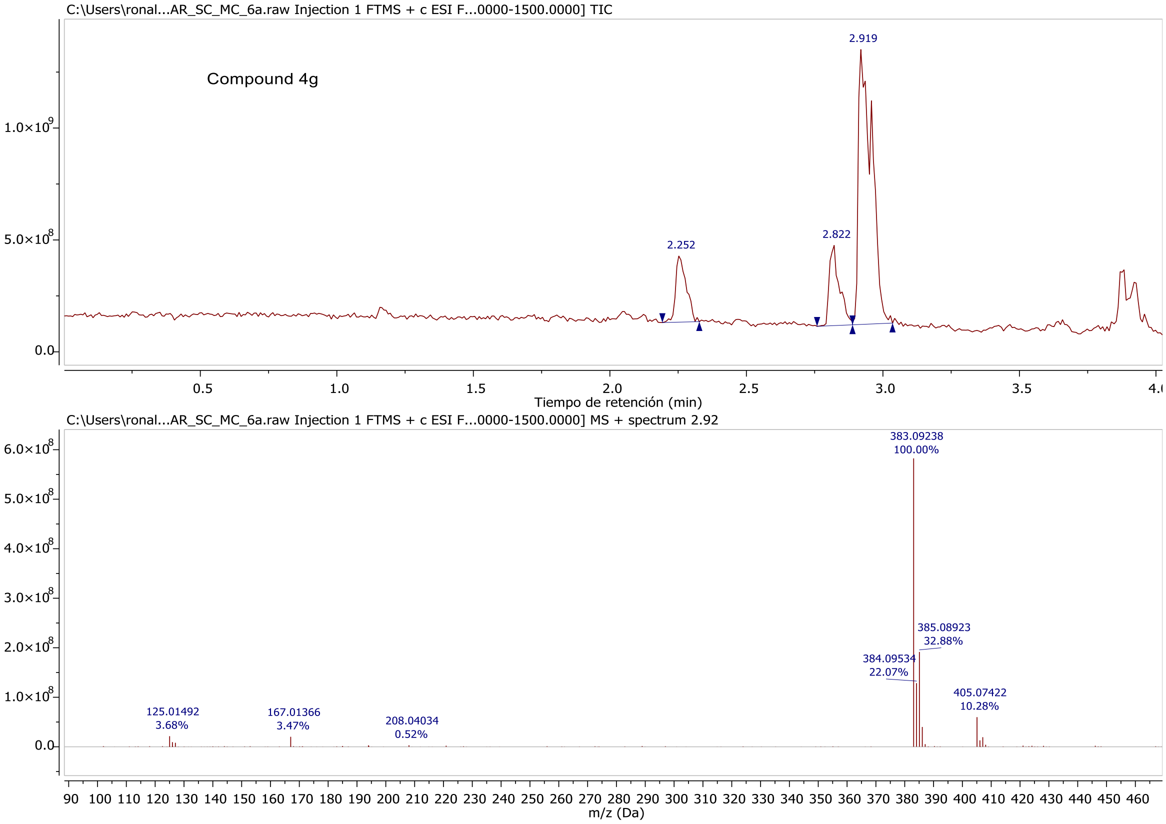
C:\Users\ronal...AR_SC_MC_6a.raw Injection 1 FTMS + c ESI F...0000-1500.0000] TIC
Compound 4g
2.919
2.822
2.252
Tiempo de retención (min)
C:\Users\ronal...AR_SC_MC_6a.raw Injection 1 FTMS + c ESI F...0000-1500.0000] MS + spectrum 2.92
383.09238
100.00%
385.08923
32.88%
384.09534
22.07%
405.07422
10.28%
125.01492
3.68%
167.01366
3.47%
208.04034
0.52%
m/z (Da)

Compound 4h

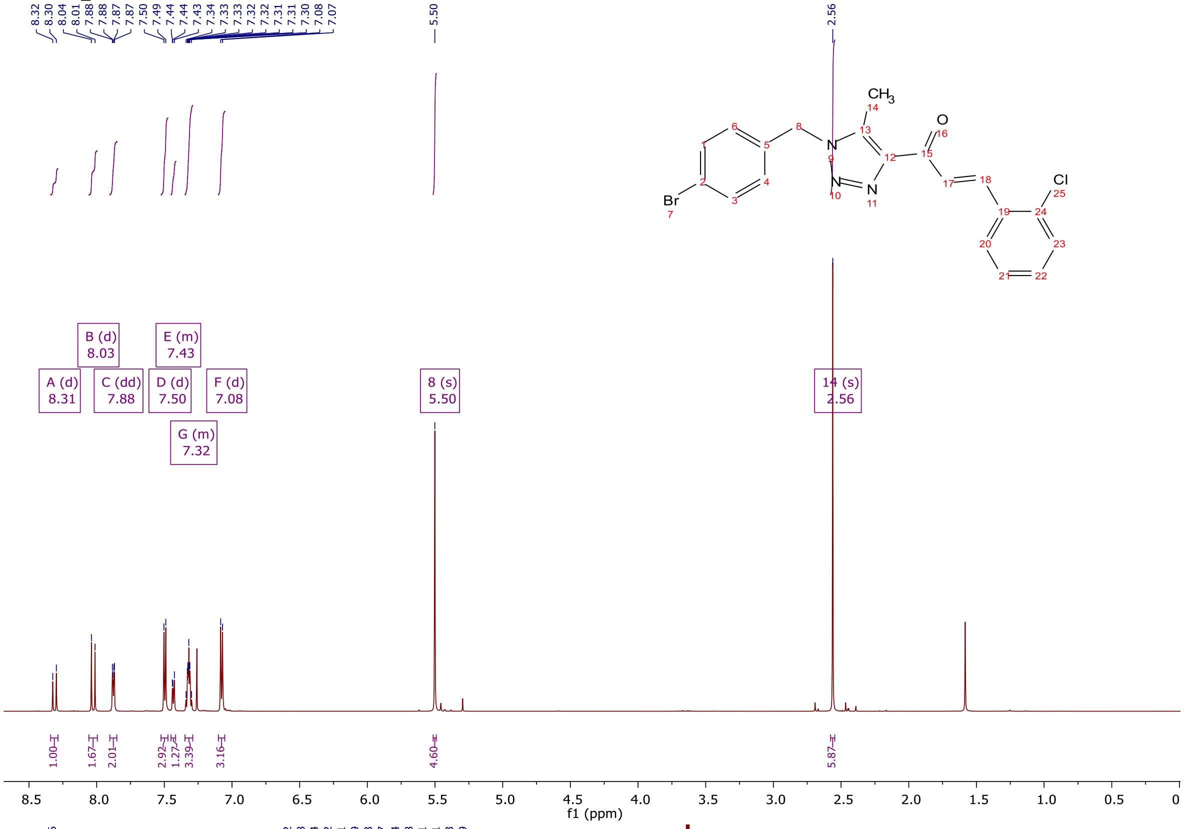

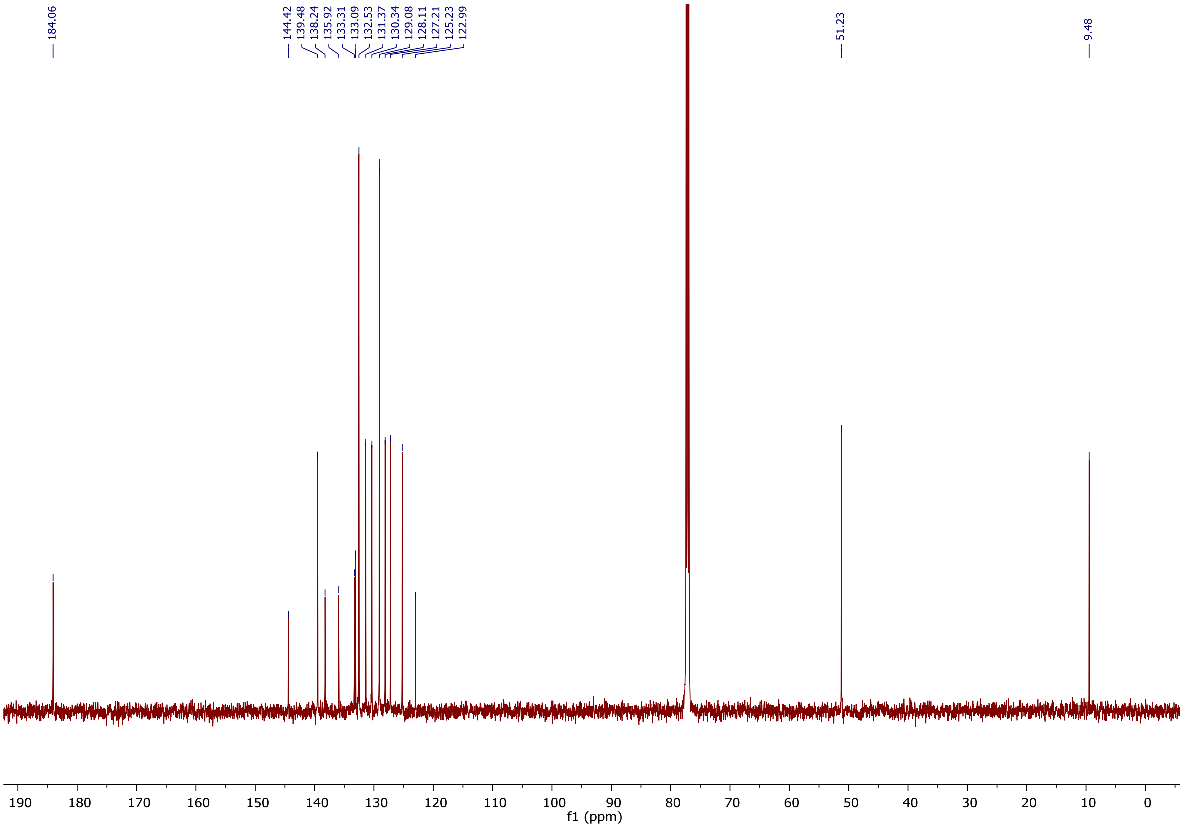

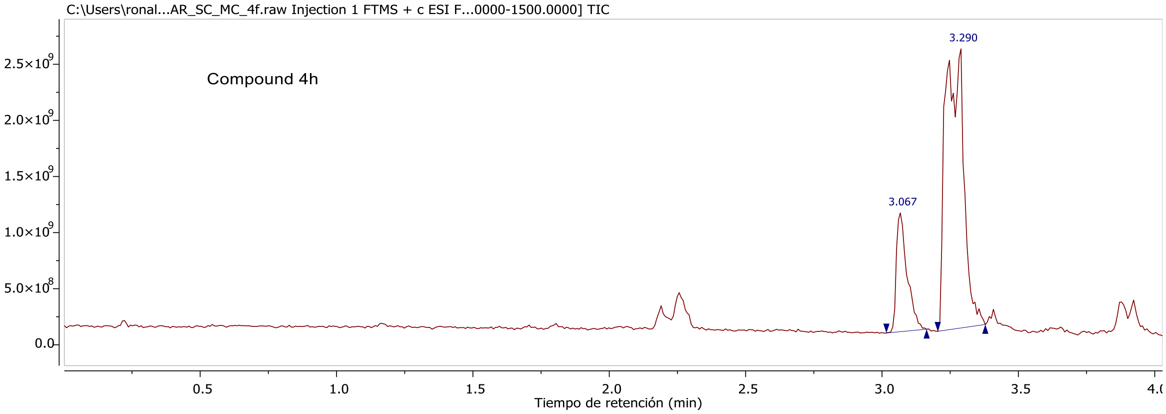
C:\Users\ronal...AR_SC_MC_4f.raw Injection 1 FTMS + c ESI F...0000-1500.0000] TIC
Compound 4h
3.290
3.067
Tiempo de retención (min)

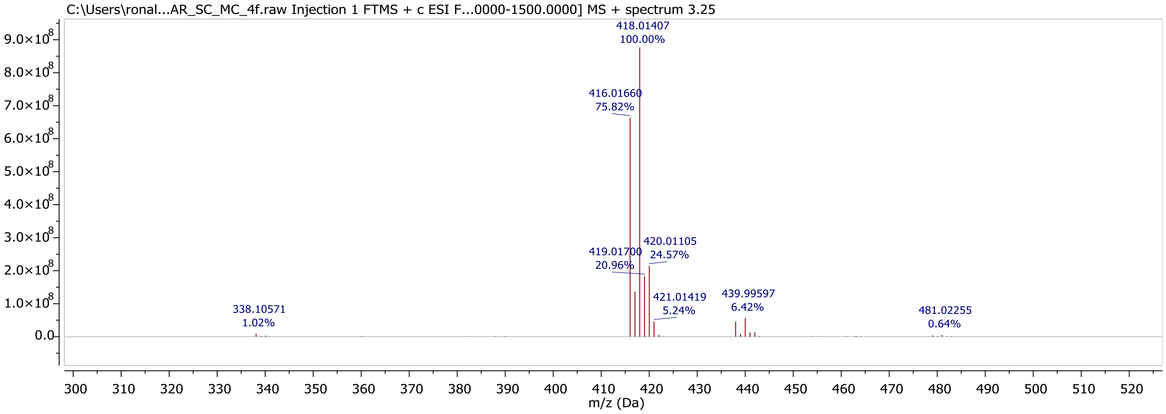
C:\Users\ronal...AR_SC_MC_4f.raw Injection 1 FTMS + c ESI F...0000-1500.0000] MS + spectrum 3.25
418.01407
100.00%
416.01660
75.82%
420.01105
24.57%
419.01700
20.96%
421.01419
5.24%
439.99597
6.42%
338.10571
1.02%
481.02255
0.64%
m/z (Da)

Compound 4i

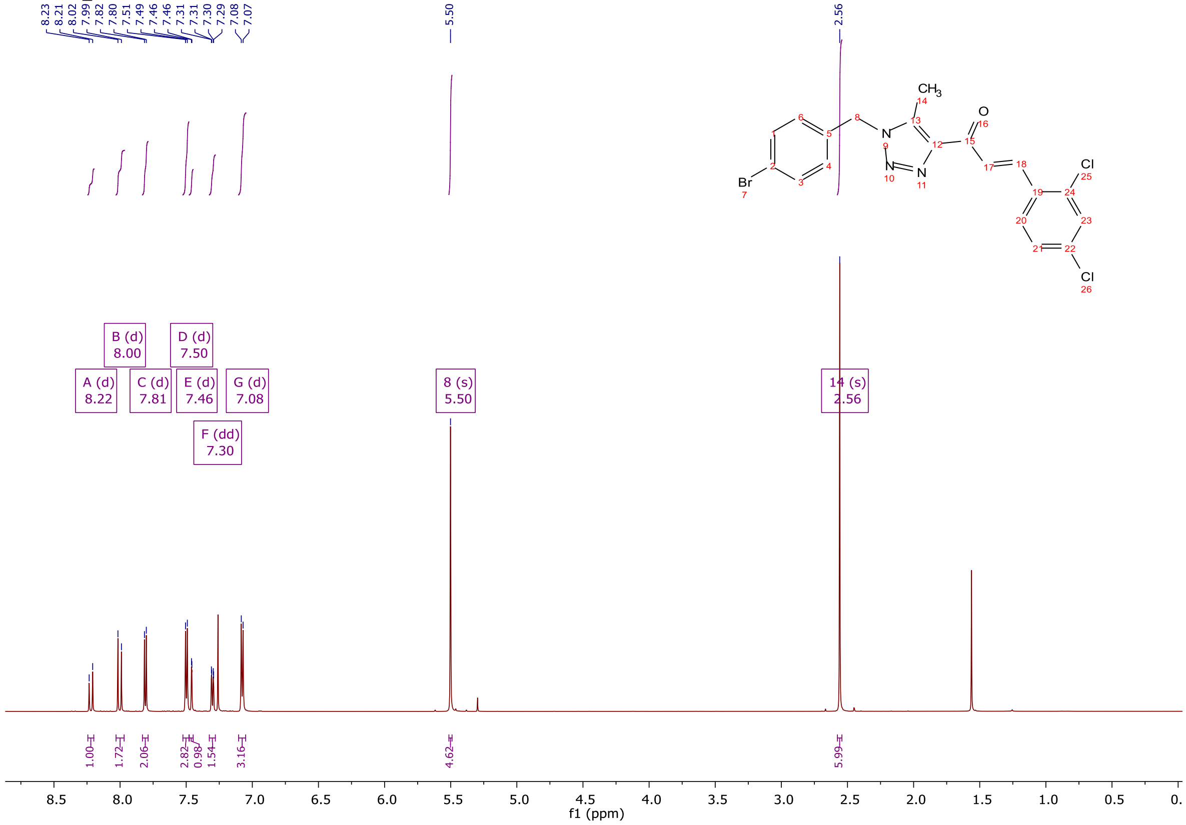

8.23
8.21
8.02
7.99
7.82
7.80
7.51
7.49
7.46
7.46
7.31
7.30
7.29
7.08
7.07
5.50
2.56
A (d) 8.22
B (d) 8.00
C (d) 7.81
D (d) 7.50
E (d) 7.46
F (dd) 7.30
G (d) 7.08
8 (s) 5.50
14 (s) 2.56
1.00
1.72
2.06
2.82
0.98
1.54
3.16
4.62
5.99
CH3
O
Cl
Cl
Br
N
N
N
f1 (ppm)

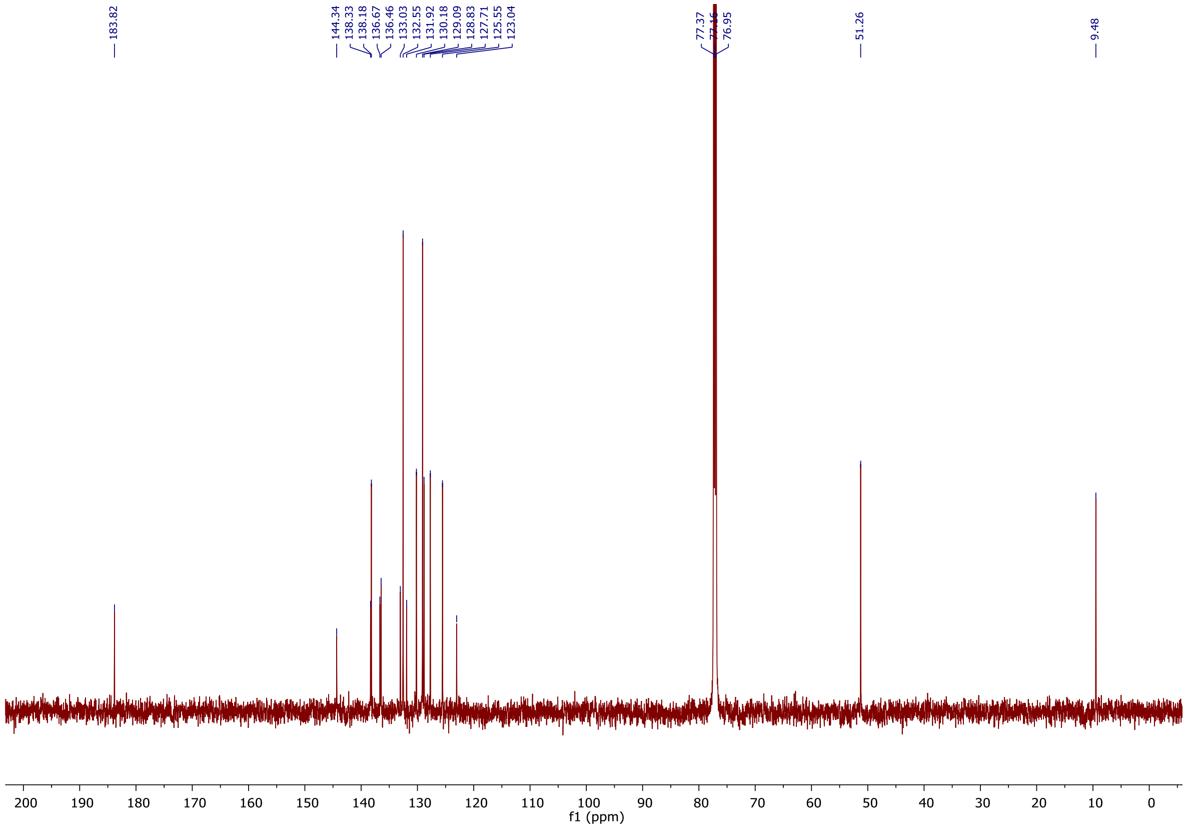

183.82
144.34
138.33
138.18
136.67
136.46
133.03
132.55
131.92
130.18
129.09
128.83
127.71
125.55
123.04
77.37
77.16
76.95
51.26
9.48
f1 (ppm)

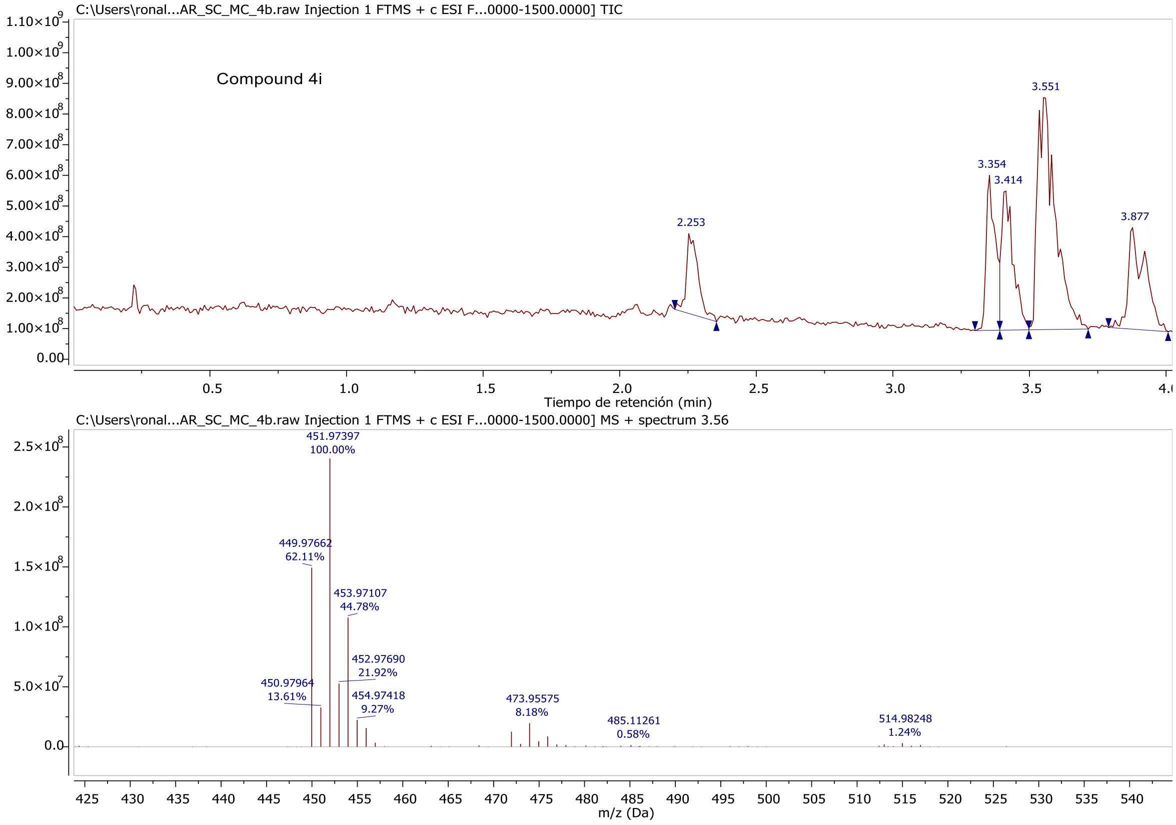
C:\Users\ronal...AR_SC_MC_4b.raw Injection 1 FTMS + c ESI F...0000-1500.0000] TIC
Compound 4i
2.253
3.354
3.414
3.551
3.877
Tiempo de retención (min)
C:\Users\ronal...AR_SC_MC_4b.raw Injection 1 FTMS + c ESI F...0000-1500.0000] MS + spectrum 3.56
451.97397
100.00%
449.97662
62.11%
453.97107
44.78%
452.97690
21.92%
450.97964
13.61%
454.97418
9.27%
473.95575
8.18%
485.11261
0.58%
514.98248
1.24%
m/z (Da)

Compound 4j

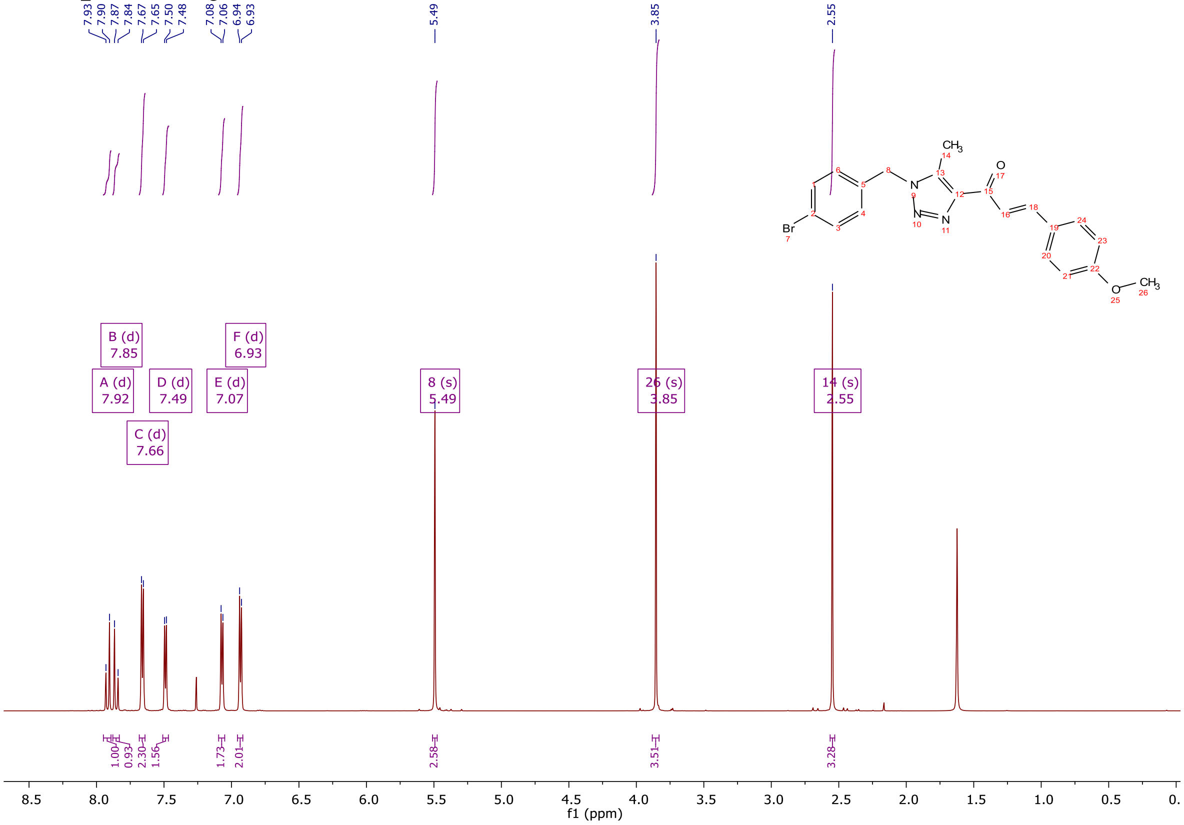
7.93
7.90
7.87
7.84
7.67
7.65
7.50
7.48
7.08
7.06
6.94
6.93
5.49
3.85
2.55
B (d) 7.85
F (d) 6.93
A (d) 7.92
D (d) 7.49
E (d) 7.07
8 (s) 5.49
26 (s) 3.85
14 (s) 2.55
C (d) 7.66
1.00
0.93
2.30
1.56
1.73
2.01
2.58
3.51
3.28
f1 (ppm)

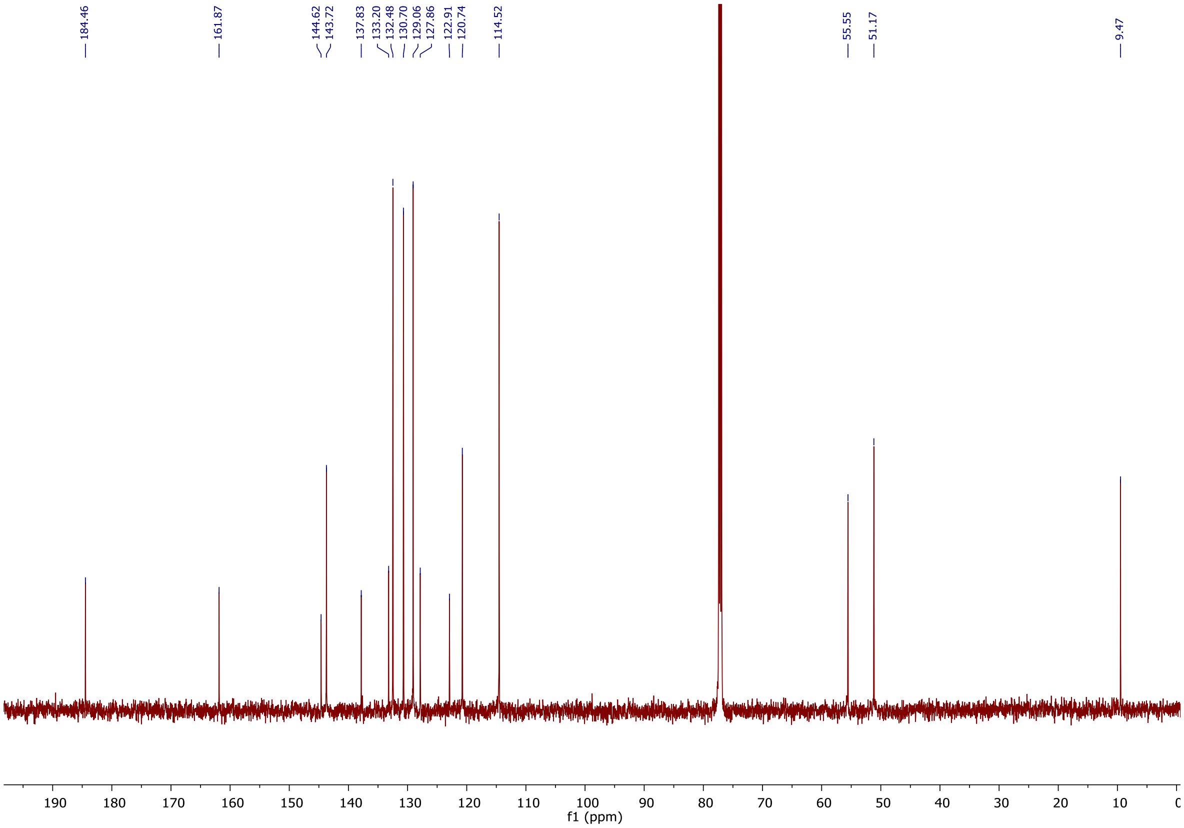
184.46
161.87
144.62
143.72
137.83
133.20
132.48
130.70
129.06
127.86
122.91
120.74
114.52
55.55
51.17
9.47
f1 (ppm)

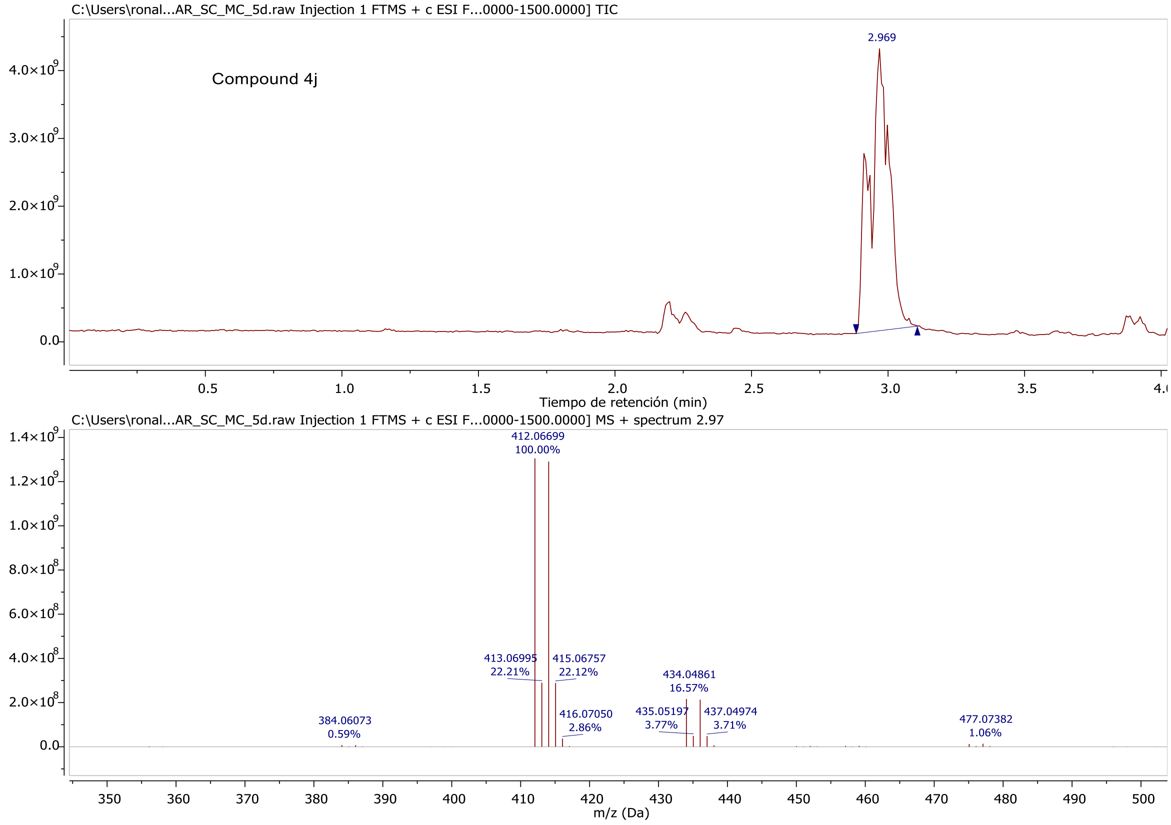
C:\Users\ronal...AR_SC_MC_5d.raw Injection 1 FTMS + c ESI F...0000-1500.0000] TIC
Compound 4j
2.969
Tiempo de retención (min)
C:\Users\ronal...AR_SC_MC_5d.raw Injection 1 FTMS + c ESI F...0000-1500.0000] MS + spectrum 2.97
412.06699
100.00%
413.06995
22.21%
415.06757
22.12%
434.04861
16.57%
384.06073
0.59%
416.07050
2.86%
435.05197
3.77%
437.04974
3.71%
477.07382
1.06%
m/z (Da)

Compound 4k

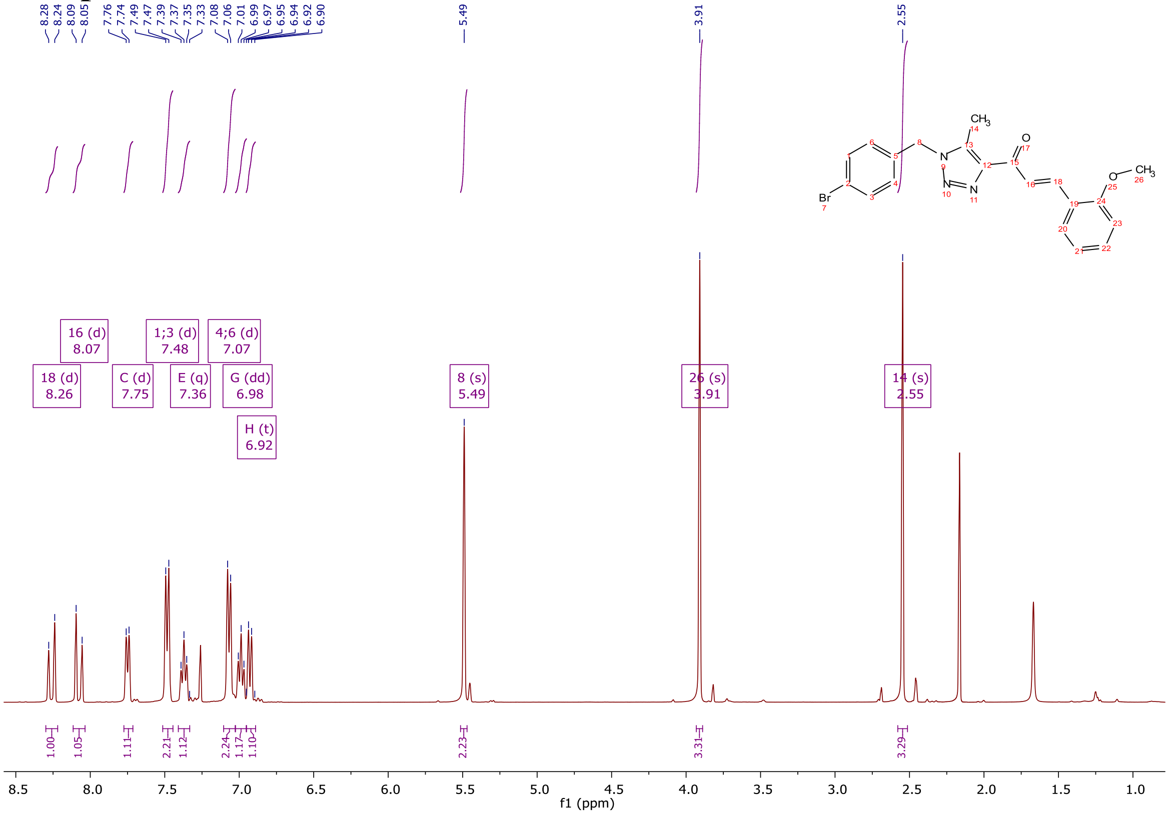
8.28 8.24 8.09 8.05 7.76 7.74 7.49 7.47 7.39 7.37 7.35 7.33 7.08 7.06 7.01 6.99 6.97 6.95 6.94 6.92 6.90
5.49
3.91
2.55
16 (d) 8.07
1;3 (d) 7.48
4;6 (d) 7.07
18 (d) 8.26
C (d) 7.75
E (q) 7.36
G (dd) 6.98
H (t) 6.92
8 (s) 5.49
26 (s) 3.91
14 (s) 2.55
1.00 1.05 1.11 2.21 1.12 2.24 1.17 1.10 2.23 3.31 3.29
f1 (ppm)
Br
CH3
O
N
CH3

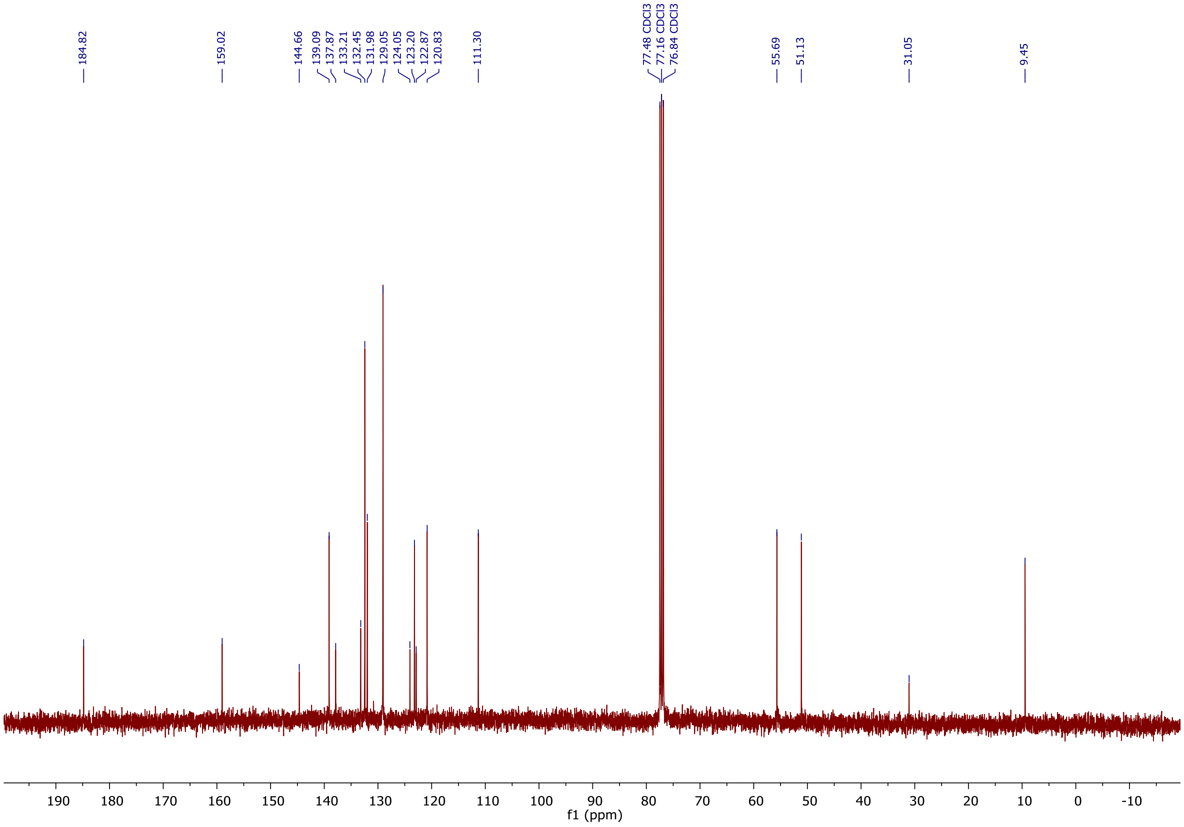
184.82
159.02
144.66 139.09 137.87 133.21 132.45 131.98 129.05 124.05 123.20 122.87 120.83
111.30
77.48 CDCl3 77.16 CDCl3 76.84 CDCl3
55.69 51.13
31.05
9.45
f1 (ppm)

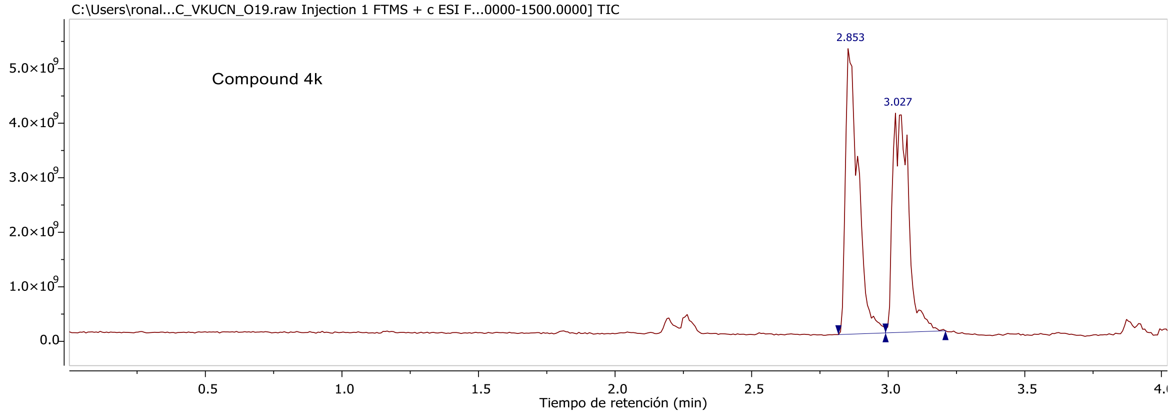
C:\Users\ronal...C_VKUCN_O19.raw Injection 1 FTMS + c ESI F...0000-1500.0000] TIC
Compound 4k
2.853
3.027
Tiempo de retención (min)

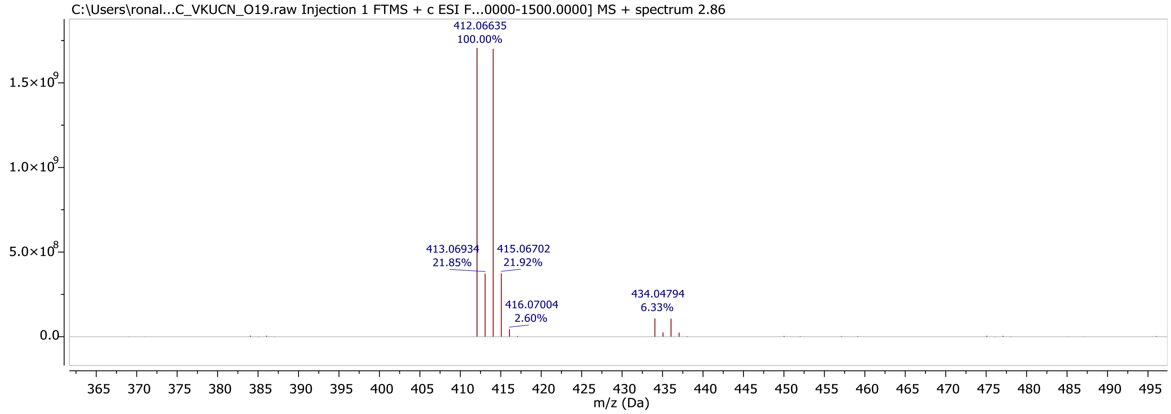
C:\Users\ronal...C_VKUCN_O19.raw Injection 1 FTMS + c ESI F...0000-1500.0000] MS + spectrum 2.86
412.06635
100.00%
413.06934
21.85%
415.06702
21.92%
416.07004
2.60%
434.04794
6.33%
m/z (Da)

Compound 4l

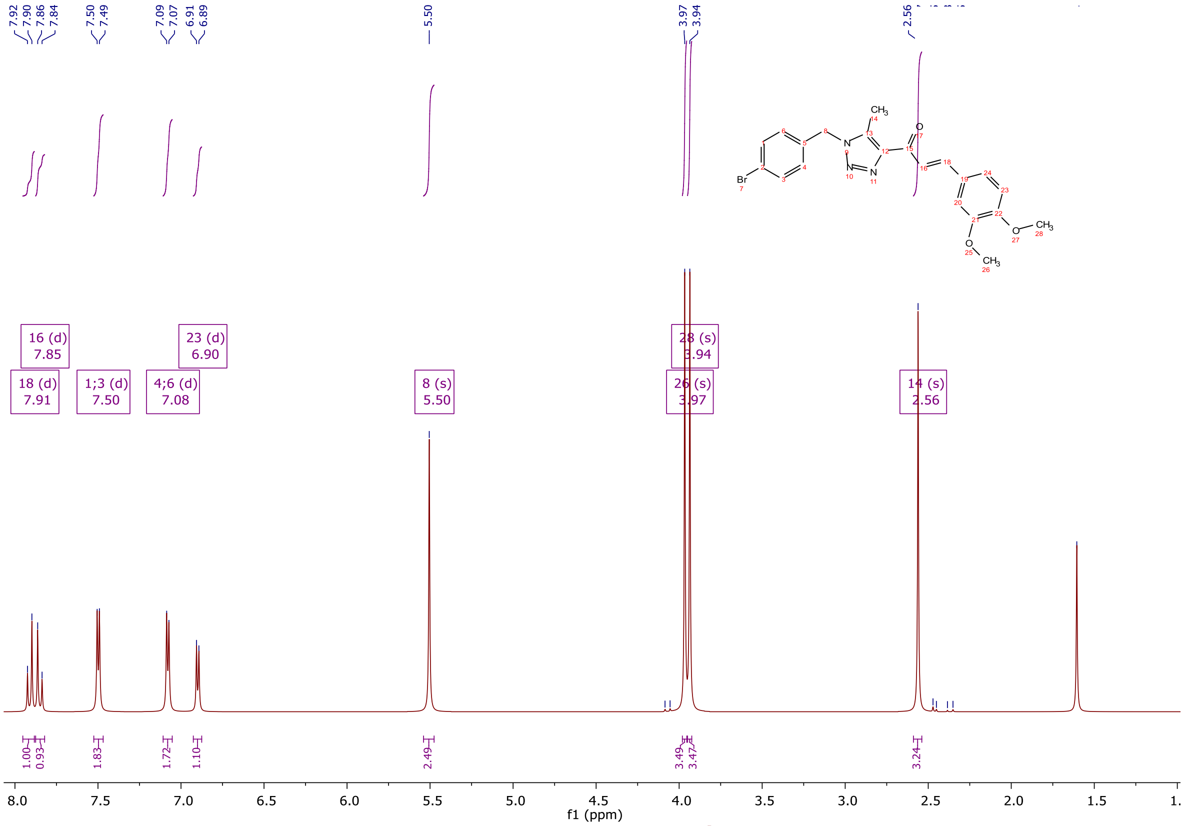
7.92 7.90 7.86 7.84 7.50 7.49 7.09 7.07 6.91 6.89 5.50 3.97 3.94 2.56
16 (d) 7.85
23 (d) 6.90
28 (s) 3.94
18 (d) 7.91
1;3 (d) 7.50
4;6 (d) 7.08
8 (s) 5.50
26 (s) 3.97
14 (s) 2.56
1.00 0.93 1.83 1.72 1.10 2.49 3.49 3.47 3.24
f1 (ppm)

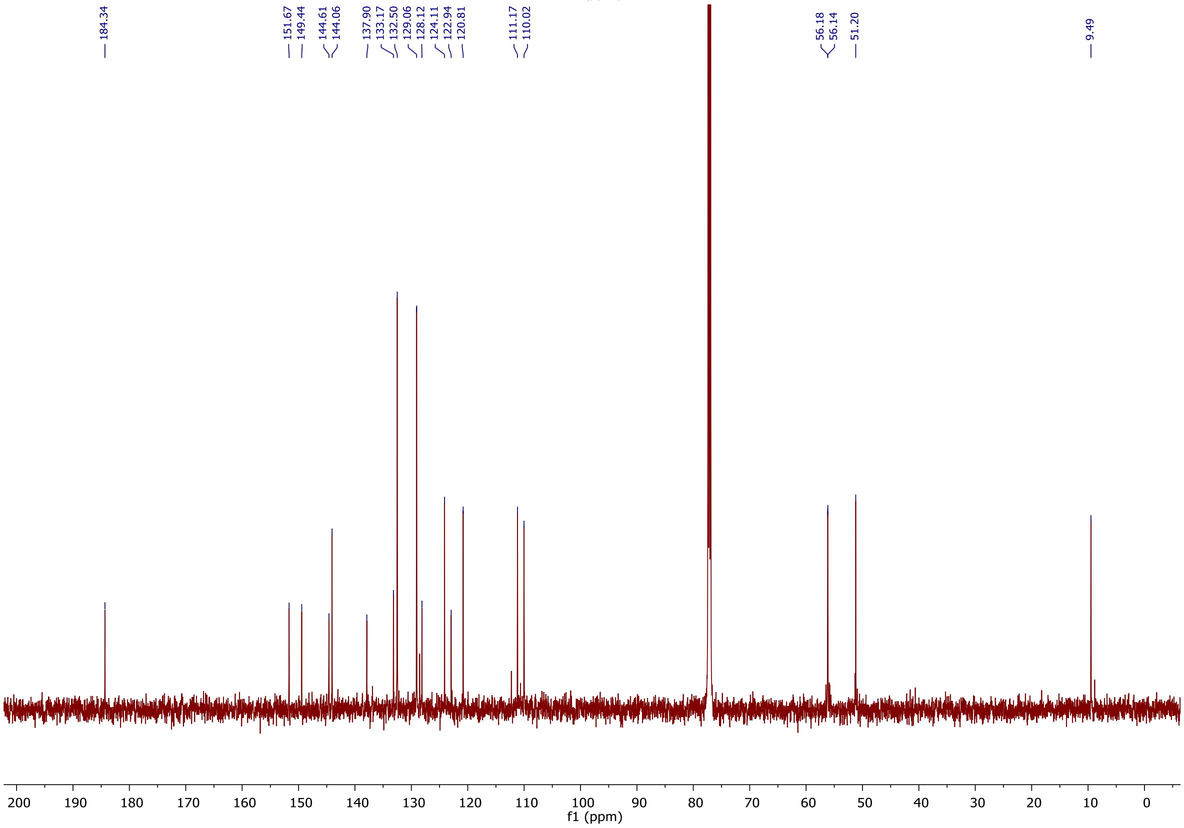
184.34
151.67 149.44 144.61 144.06 137.90 133.17 132.50 129.06 128.12 124.11 122.94 120.81 111.17 110.02
56.18 56.14 51.20
9.49
f1 (ppm)

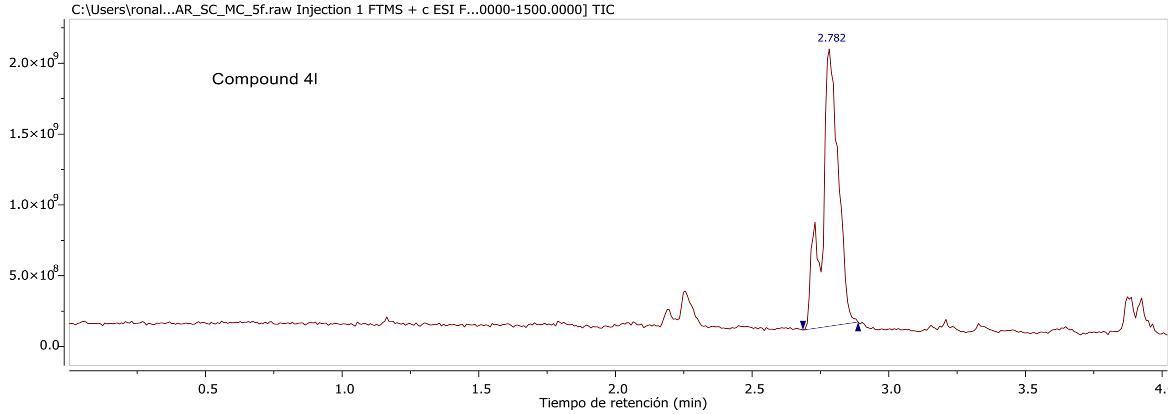

C:\Users\ronal...AR_SC_MC_5f.raw Injection 1 FTMS + c ESI F...0000-1500.0000] TIC
Compound 4l
2.782
Tiempo de retención (min)

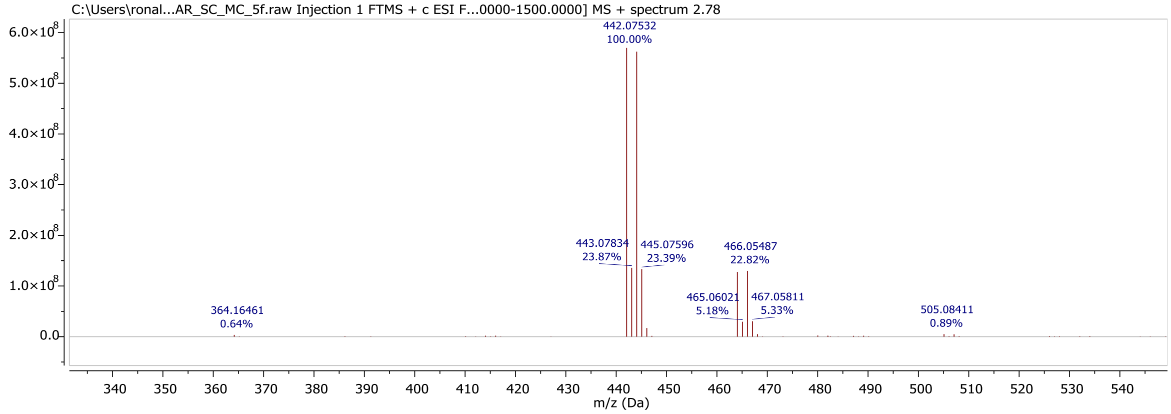

C:\Users\ronal...AR_SC_MC_5f.raw Injection 1 FTMS + c ESI F...0000-1500.0000] MS + spectrum 2.78
442.07532
100.00%
443.07834
23.87%
445.07596
23.39%
466.05487
22.82%
465.06021
5.18%
467.05811
5.33%
364.16461
0.64%
505.08411
0.89%
m/z (Da)

Compound 4m

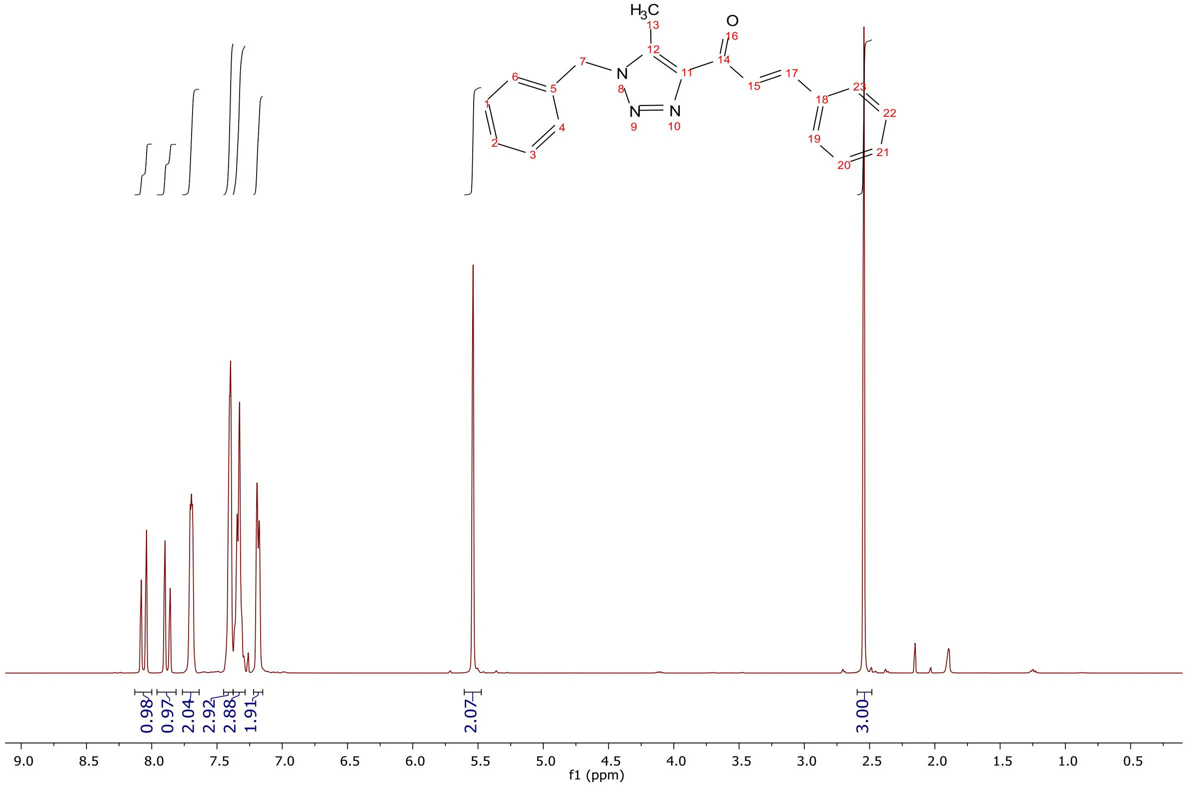

0.98
0.97
2.04
2.92
2.88
1.91
2.07
3.00
f1 (ppm)

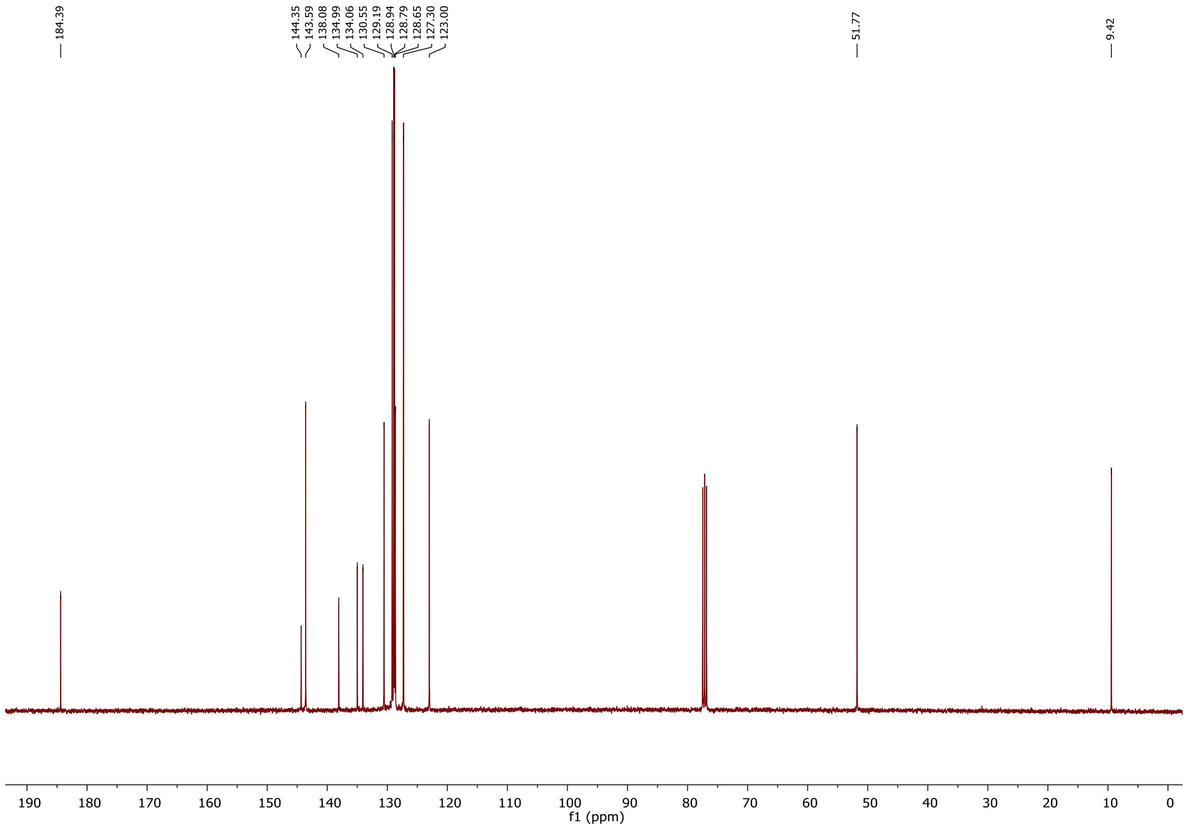

184.39
144.35
143.59
138.08
134.99
134.06
130.55
129.19
128.94
128.79
128.65
127.30
123.00
51.77
9.42
f1 (ppm)

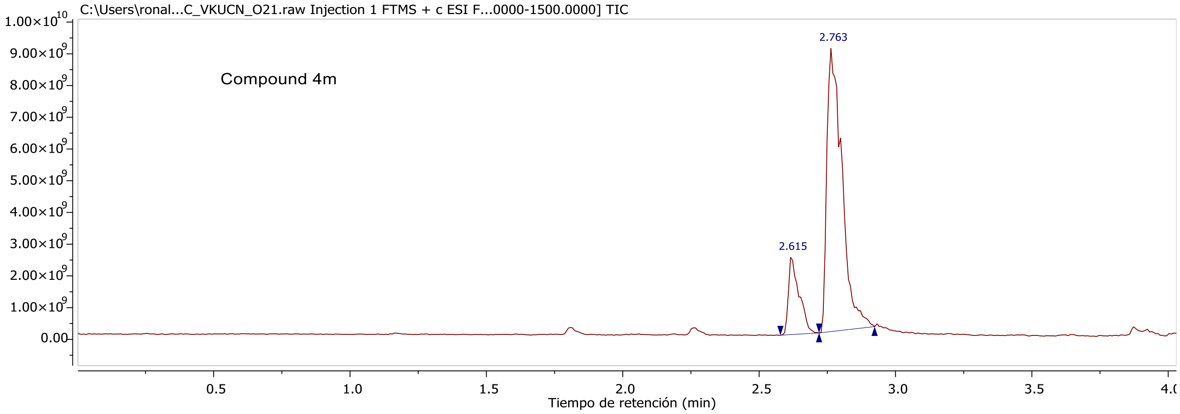
C:\Users\ronal...C_VKUCN_O21.raw Injection 1 FTMS + c ESI F...0000-1500.0000] TIC
Compound 4m
2.763
2.615
Tiempo de retención (min)

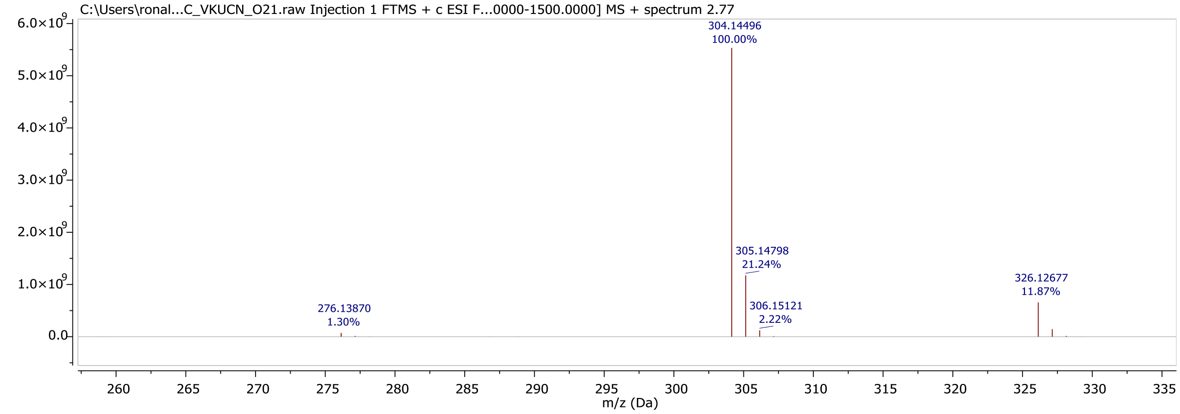
C:\Users\ronal...C_VKUCN_O21.raw Injection 1 FTMS + c ESI F...0000-1500.0000] MS + spectrum 2.77
304.14496
100.00%
305.14798
21.24%
306.15121
2.22%
326.12677
11.87%
276.13870
1.30%
m/z (Da)

Compound 4n

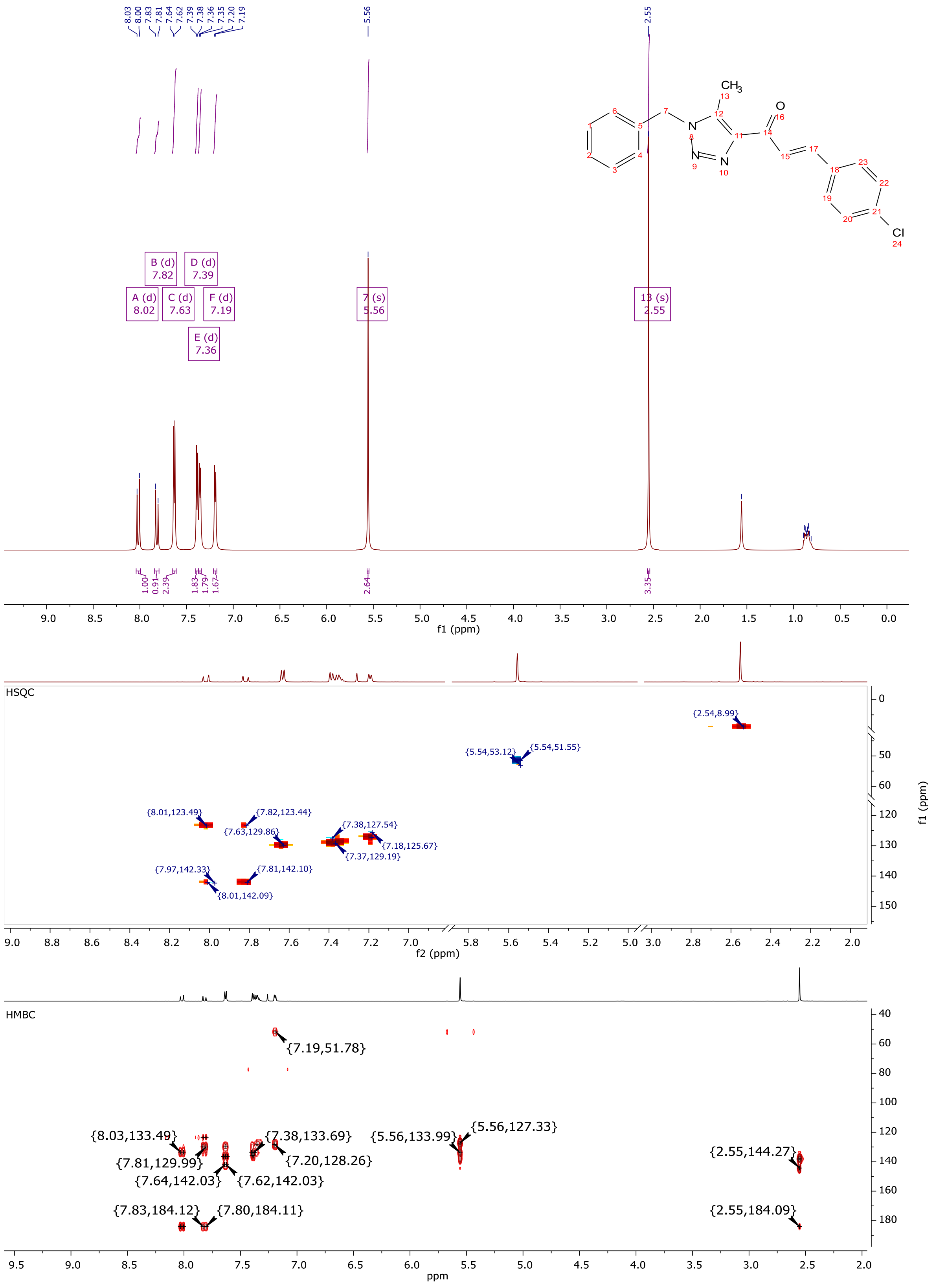

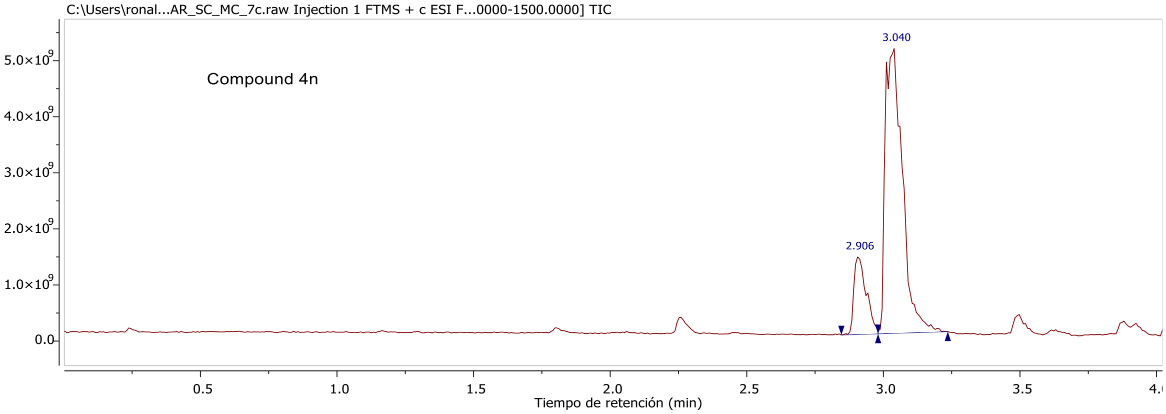
C:\Users\ronal...AR_SC_MC_7c.raw Injection 1 FTMS + c ESI F...0000-1500.0000] TIC
Compound 4n
3.040
2.906
Tiempo de retención (min)

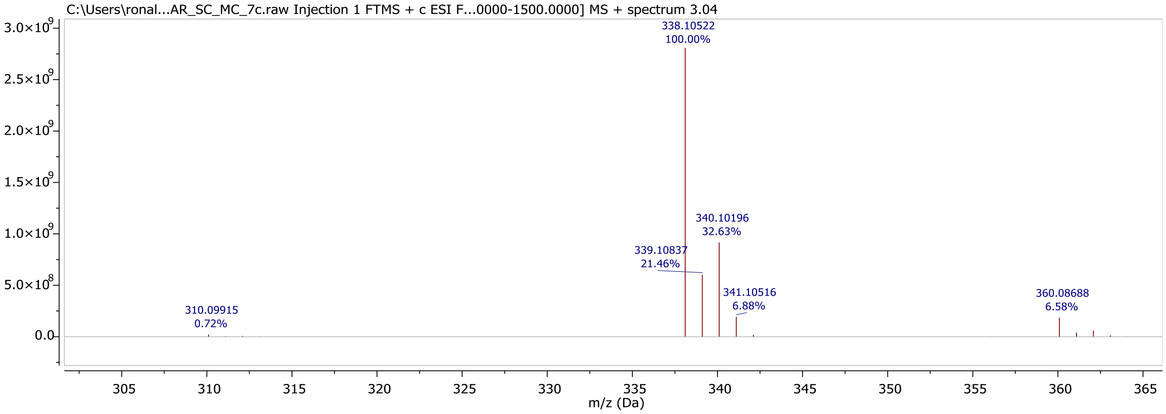
C:\Users\ronal...AR_SC_MC_7c.raw Injection 1 FTMS + c ESI F...0000-1500.0000] MS + spectrum 3.04
338.10522
100.00%
340.10196
32.63%
339.10837
21.46%
341.10516
6.88%
310.09915
0.72%
360.08688
6.58%
m/z (Da)

Compound 4o

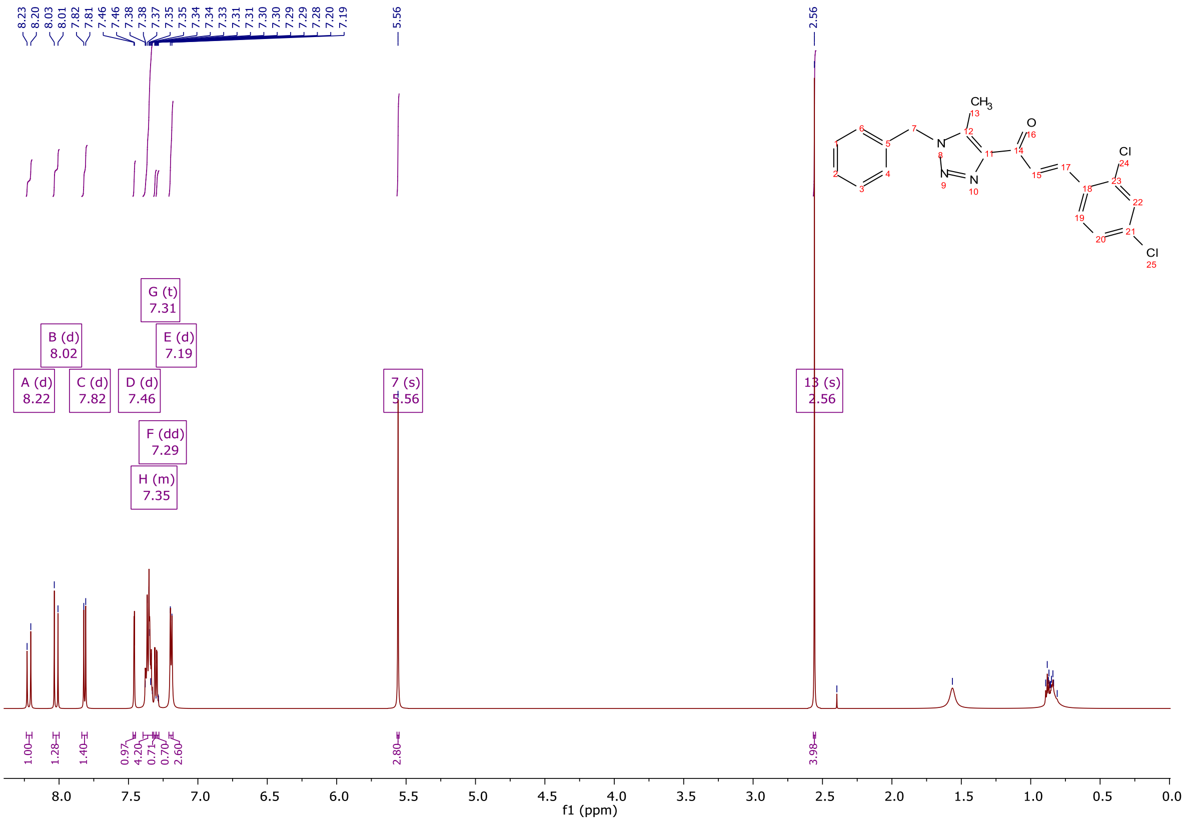

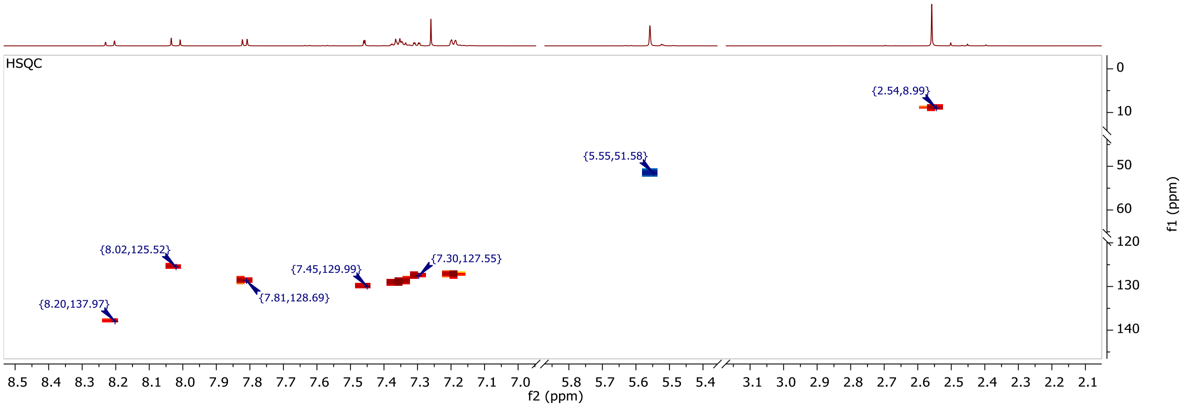

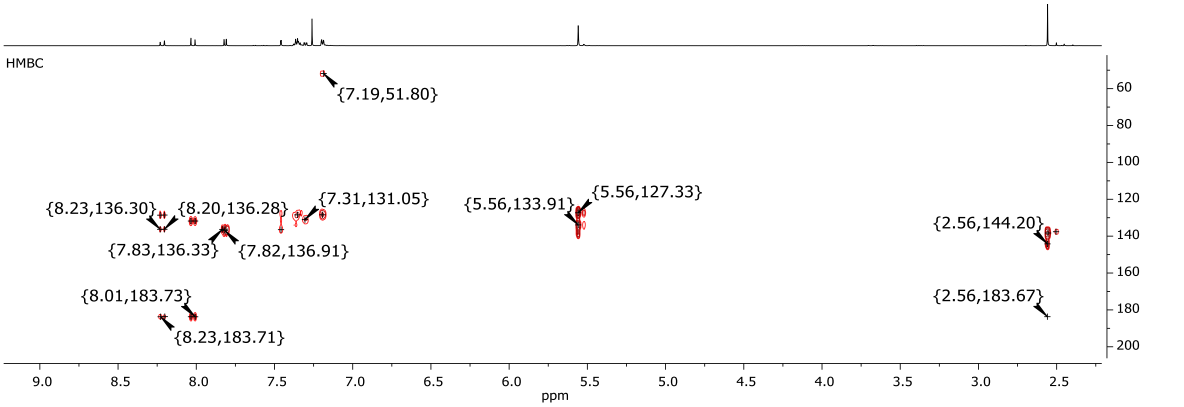

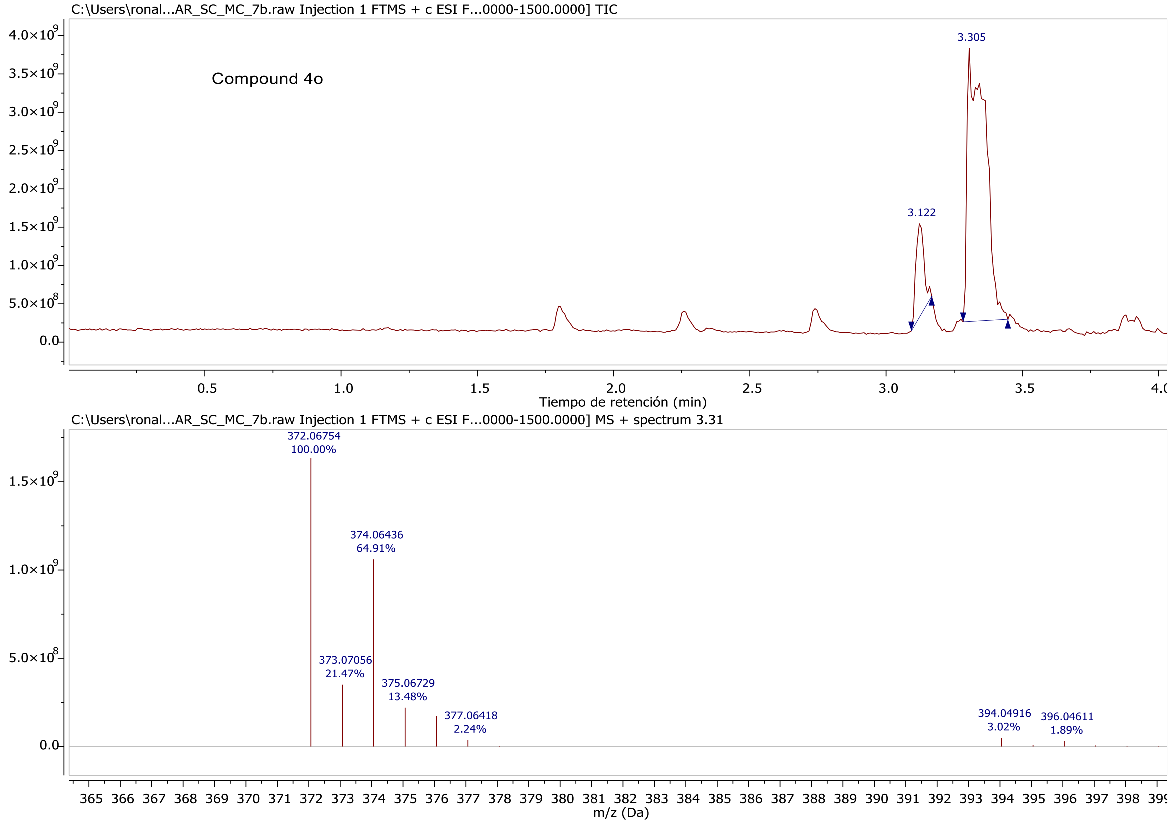
C:\Users\ronal...AR_SC_MC_7b.raw Injection 1 FTMS + c ESI F...0000-1500.0000] TIC
Compound 4o
3.305
3.122
Tiempo de retención (min)
C:\Users\ronal...AR_SC_MC_7b.raw Injection 1 FTMS + c ESI F...0000-1500.0000] MS + spectrum 3.31
372.06754
100.00%
374.06436
64.91%
373.07056
21.47%
375.06729
13.48%
377.06418
2.24%
394.04916
3.02%
396.04611
1.89%
m/z (Da)

Compound 4p

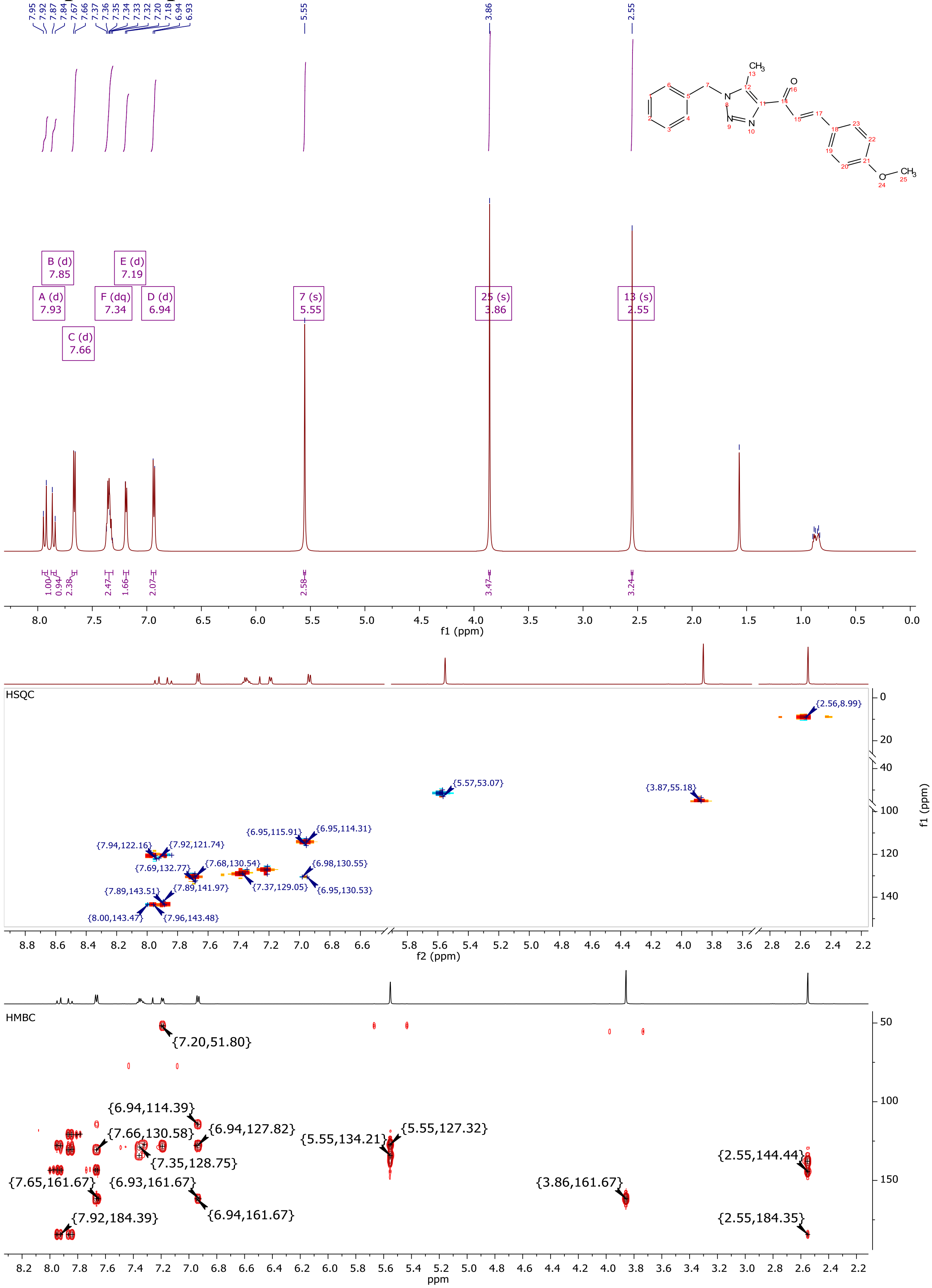

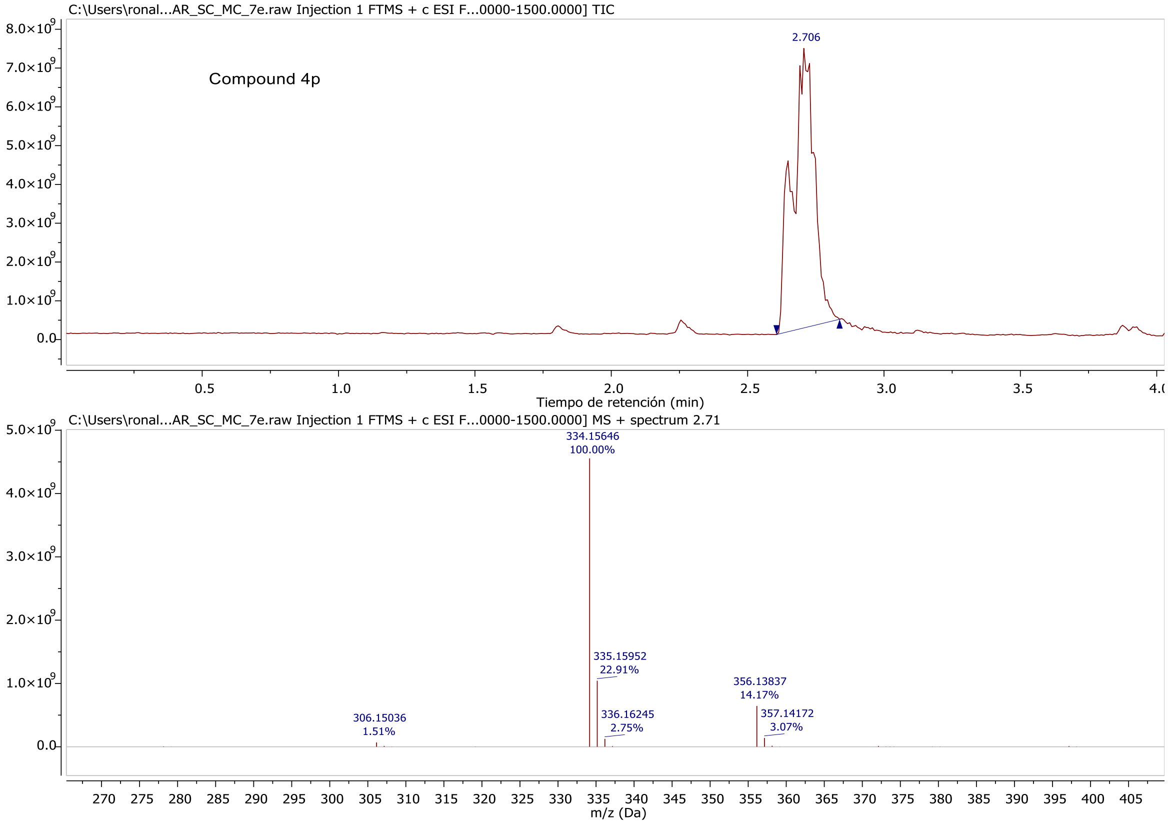
C:\Users\ronal...AR_SC_MC_7e.raw Injection 1 FTMS + c ESI F...0000-1500.0000] TIC
Compound 4p
2.706
Tiempo de retención (min)
C:\Users\ronal...AR_SC_MC_7e.raw Injection 1 FTMS + c ESI F...0000-1500.0000] MS + spectrum 2.71
334.15646
100.00%
335.15952
22.91%
336.16245
2.75%
356.13837
14.17%
357.14172
3.07%
306.15036
1.51%
m/z (Da)

Compound 4q

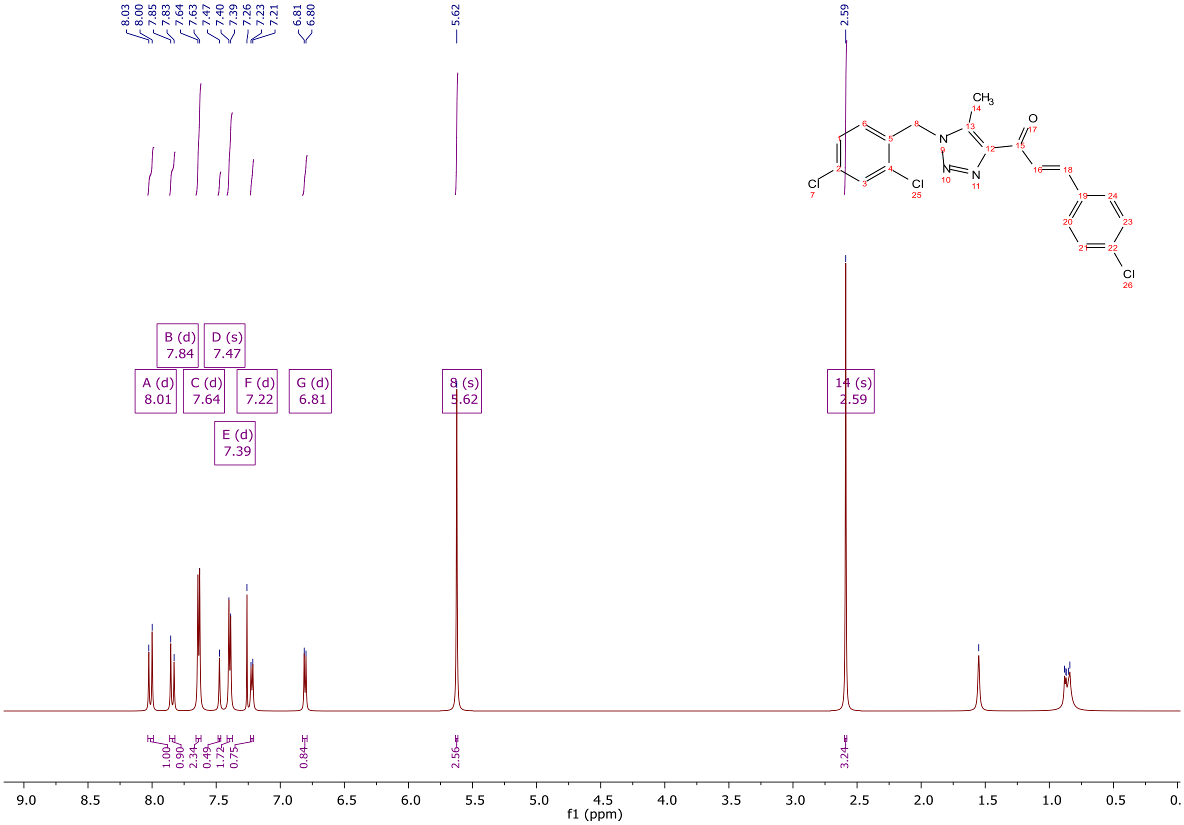

8.03
8.00
7.85
7.83
7.64
7.63
7.47
7.40
7.39
7.26
7.23
7.21
6.81
6.80
5.62
2.59
A (d) 8.01
B (d) 7.84
C (d) 7.64
D (s) 7.47
E (d) 7.39
F (d) 7.22
G (d) 6.81
8 (s) 5.62
14 (s) 2.59
1.00
0.90
2.34
0.49
1.72
0.75
0.84
2.56
3.24
f1 (ppm)

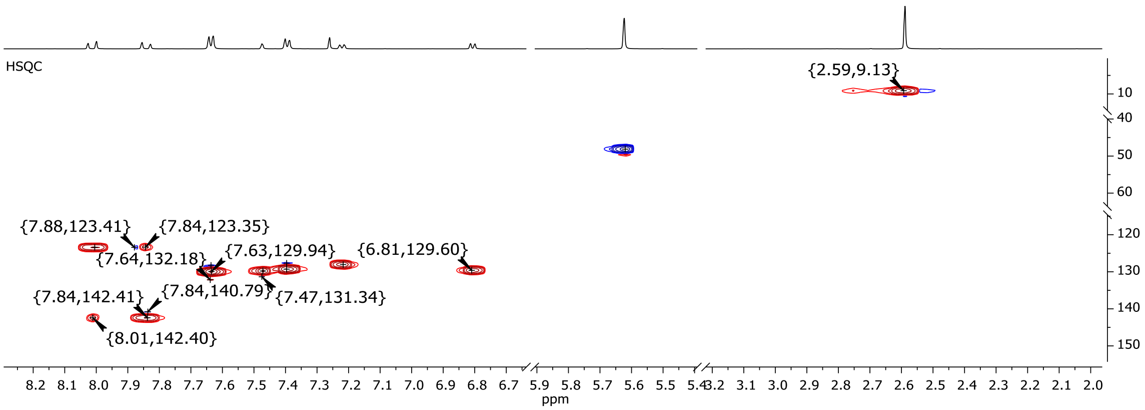

HSQC
{2.59,9.13}
{7.88,123.41}
{7.84,123.35}
{7.64,132.18}
{7.63,129.94}
{6.81,129.60}
{7.84,142.41}
{7.84,140.79}
{7.47,131.34}
{8.01,142.40}
ppm

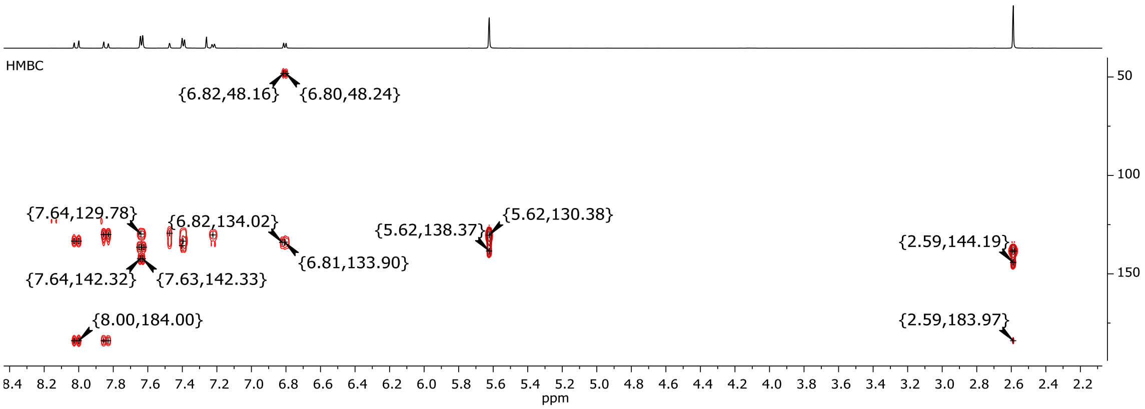

HMBC
{6.82,48.16}
{6.80,48.24}
{7.64,129.78}
{6.82,134.02}
{5.62,138.37}
{5.62,130.38}
{2.59,144.19}
{6.81,133.90}
{7.64,142.32}
{7.63,142.33}
{8.00,184.00}
{2.59,183.97}
ppm

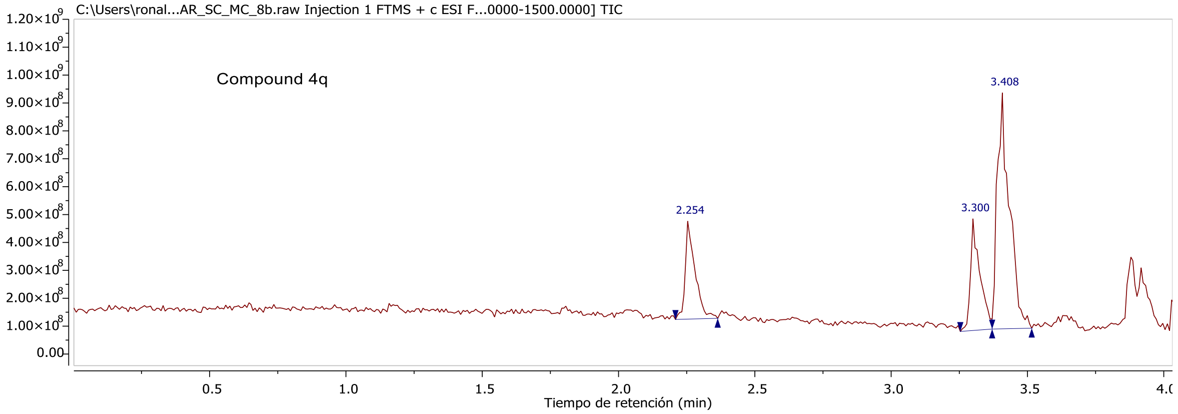
C:\Users\ronal...AR_SC_MC_8b.raw Injection 1 FTMS + c ESI F...0000-1500.0000] TIC
Compound 4q
3.408
2.254
3.300
Tiempo de retención (min)

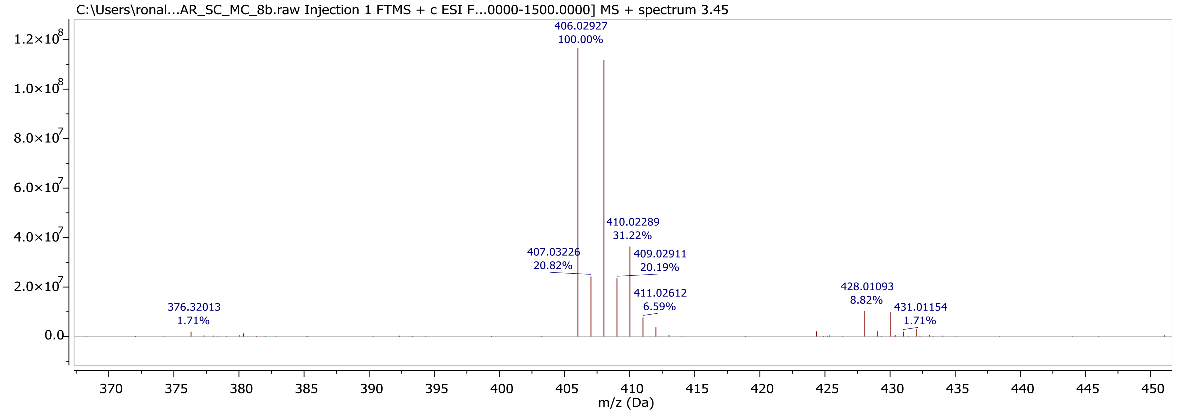
C:\Users\ronal...AR_SC_MC_8b.raw Injection 1 FTMS + c ESI F...0000-1500.0000] MS + spectrum 3.45
406.02927
100.00%
410.02289
31.22%
407.03226
20.82%
409.02911
20.19%
411.02612
6.59%
376.32013
1.71%
428.01093
8.82%
431.01154
1.71%
m/z (Da)

Compound 4r

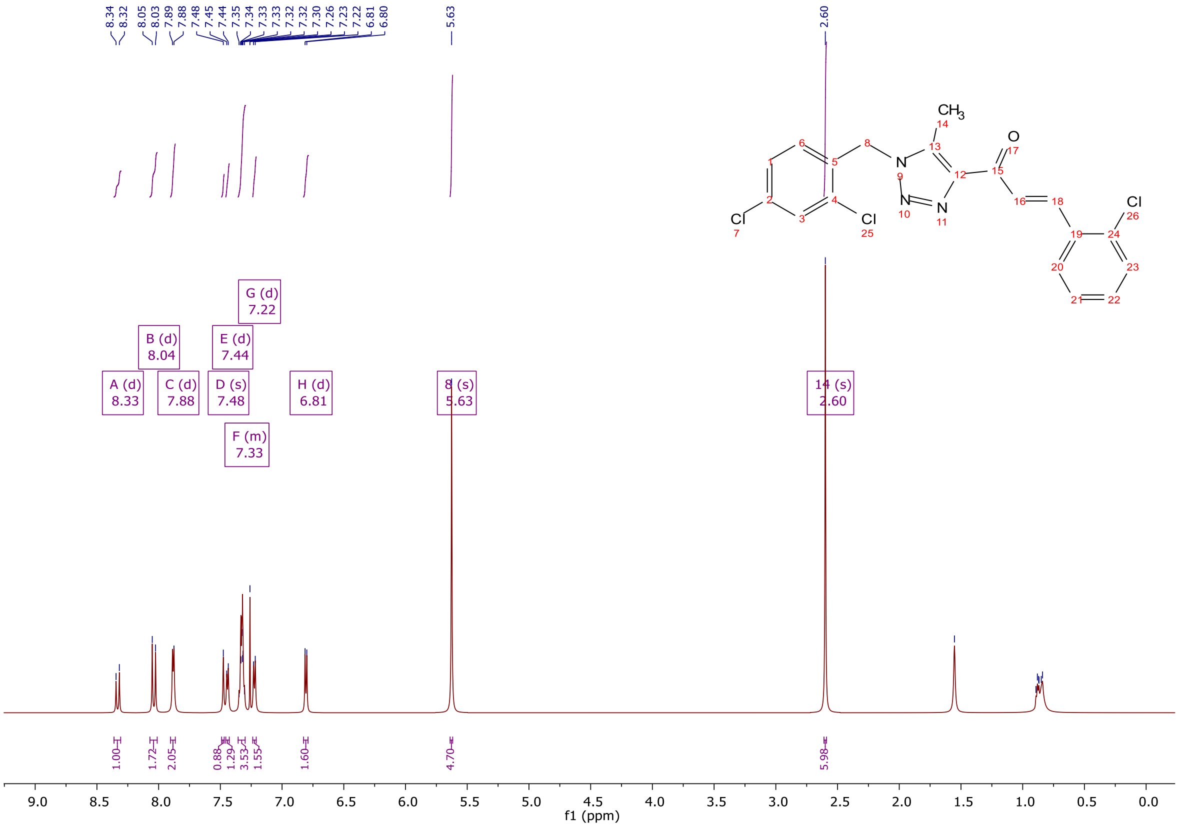

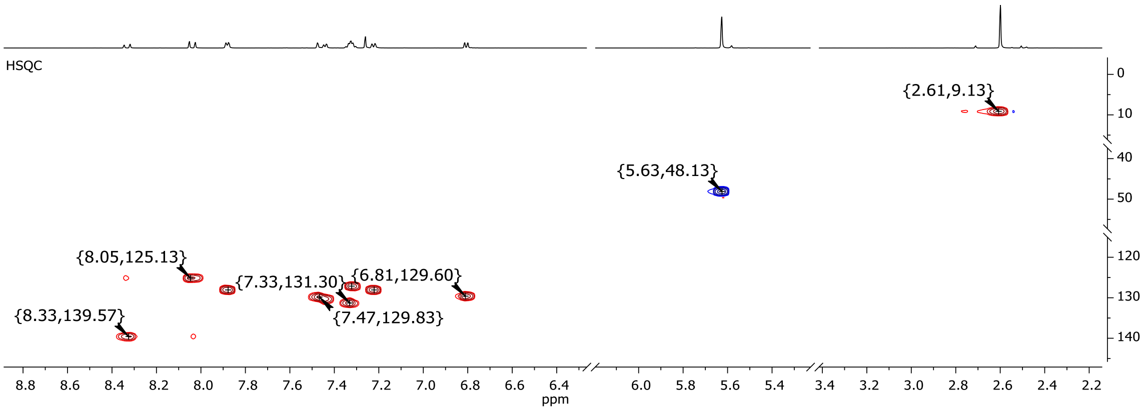
HSQC
{2.61,9.13}
{5.63,48.13}
{8.05,125.13}
{7.33,131.30}
{6.81,129.60}
{8.33,139.57}
{7.47,129.83}
ppm

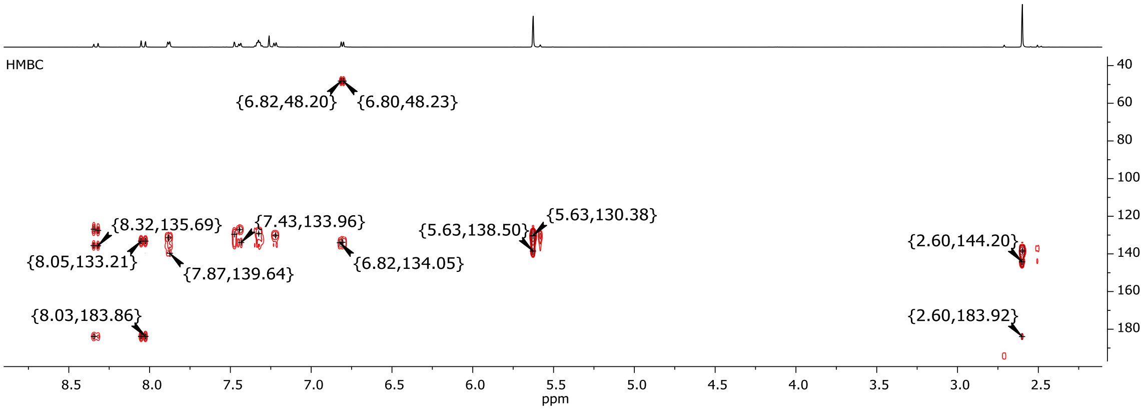
HMBC
{6.82,48.20}
{6.80,48.23}
{8.32,135.69}
{7.43,133.96}
{5.63,138.50}
{5.63,130.38}
{2.60,144.20}
{8.05,133.21}
{7.87,139.64}
{6.82,134.05}
{8.03,183.86}
{2.60,183.92}
ppm

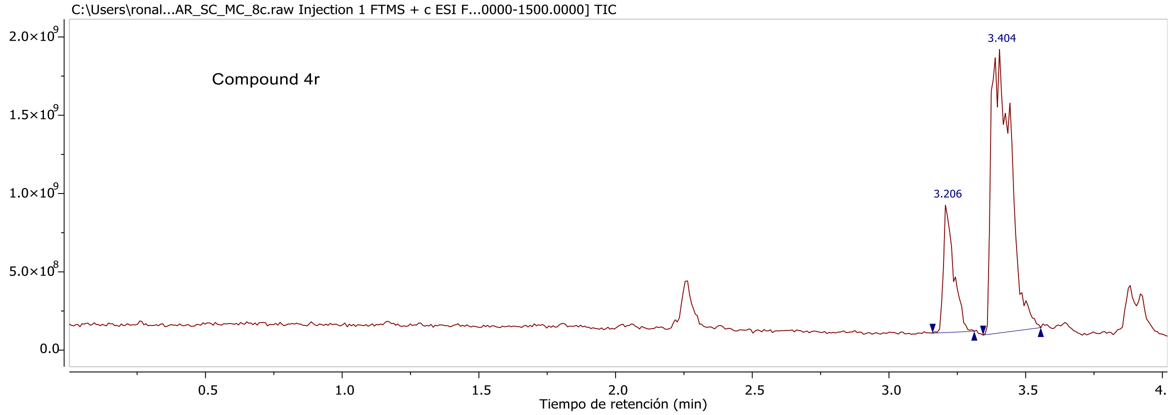
C:\Users\ronal...AR_SC_MC_8c.raw Injection 1 FTMS + c ESI F...0000-1500.0000] TIC
Compound 4r
3.404
3.206
Tiempo de retención (min)

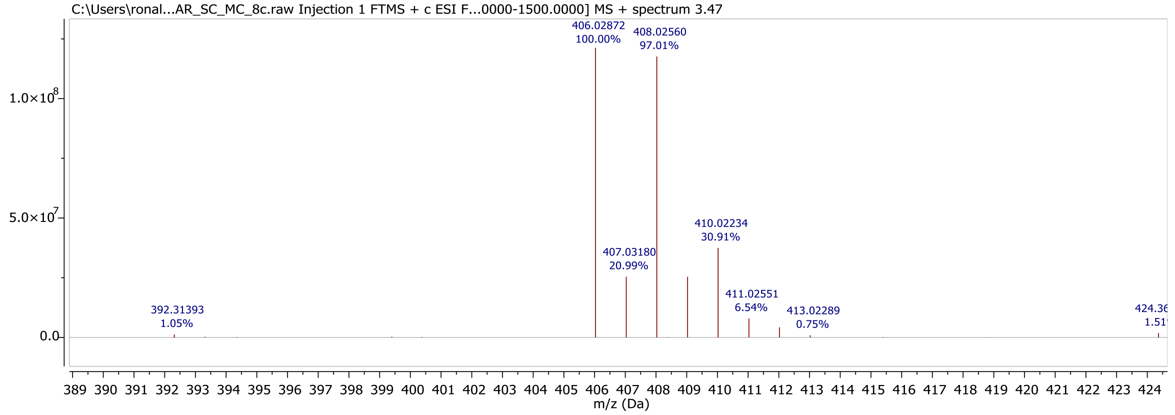
C:\Users\ronal...AR_SC_MC_8c.raw Injection 1 FTMS + c ESI F...0000-1500.0000] MS + spectrum 3.47
406.02872
100.00%
408.02560
97.01%
410.02234
30.91%
407.03180
20.99%
411.02551
6.54%
413.02289
0.75%
392.31393
1.05%
m/z (Da)

Compound 4s

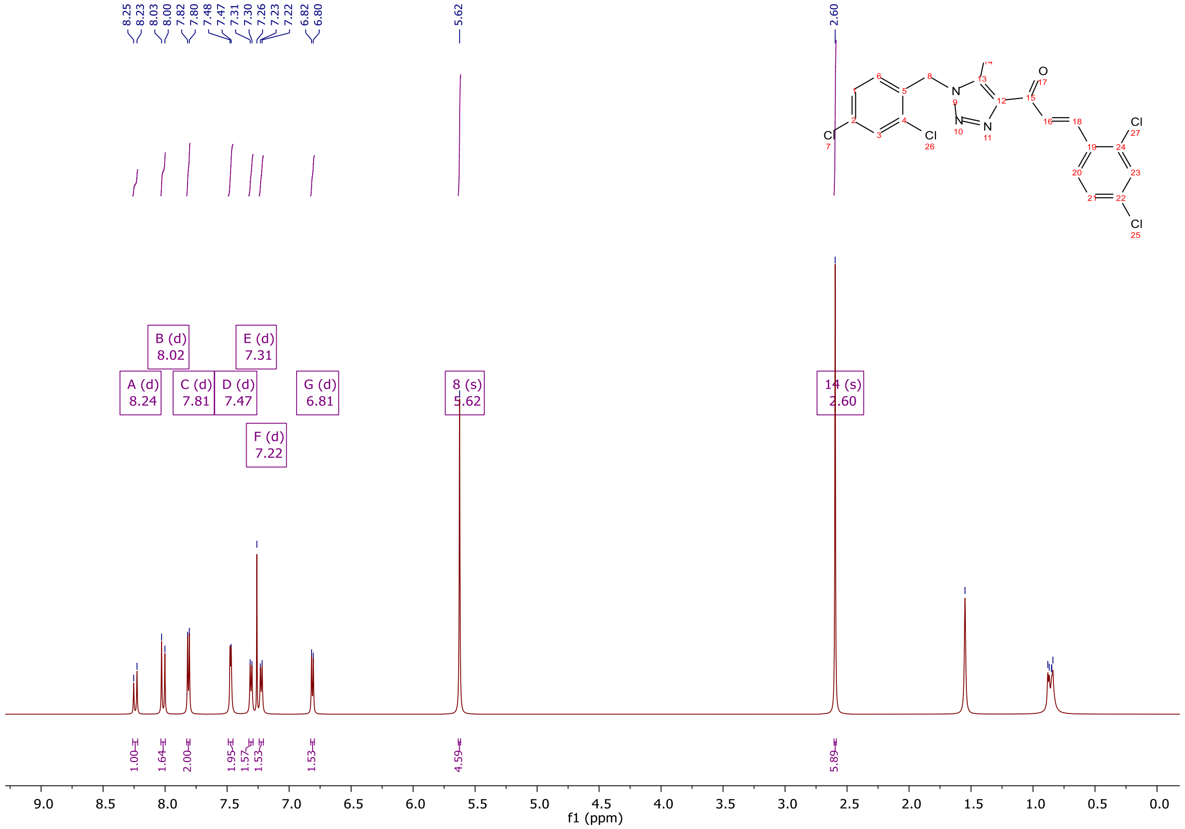

8.25
8.23
8.03
8.00
7.82
7.80
7.48
7.47
7.31
7.30
7.26
7.23
7.22
6.82
6.80
5.62
2.60
A (d) 8.24
B (d) 8.02
C (d) 7.81
D (d) 7.47
E (d) 7.31
F (d) 7.22
G (d) 6.81
8 (s) 5.62
14 (s) 2.60
1.00
1.64
2.00
1.95
1.57
1.53
1.53
4.59
5.89
f1 (ppm)
O
Cl
Cl
Cl
Cl
N
N
N

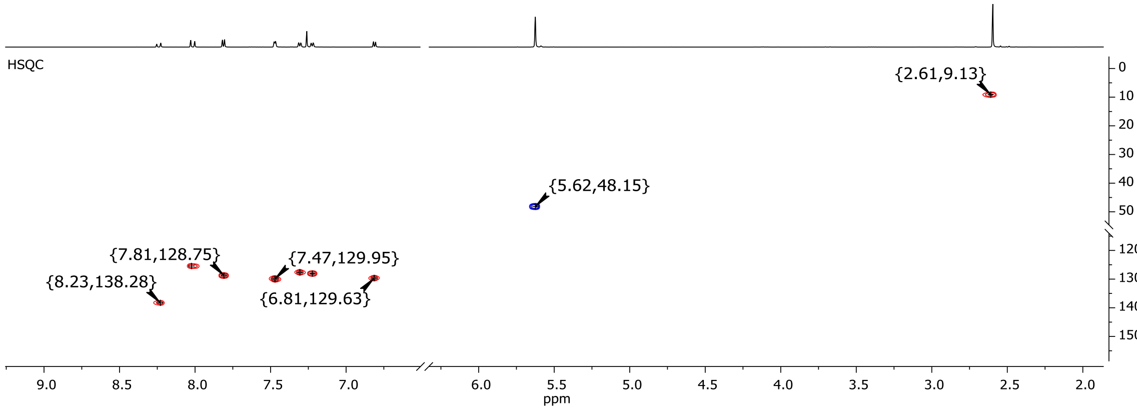

HSQC
{2.61,9.13}
{5.62,48.15}
{7.81,128.75}
{7.47,129.95}
{8.23,138.28}
{6.81,129.63}
ppm

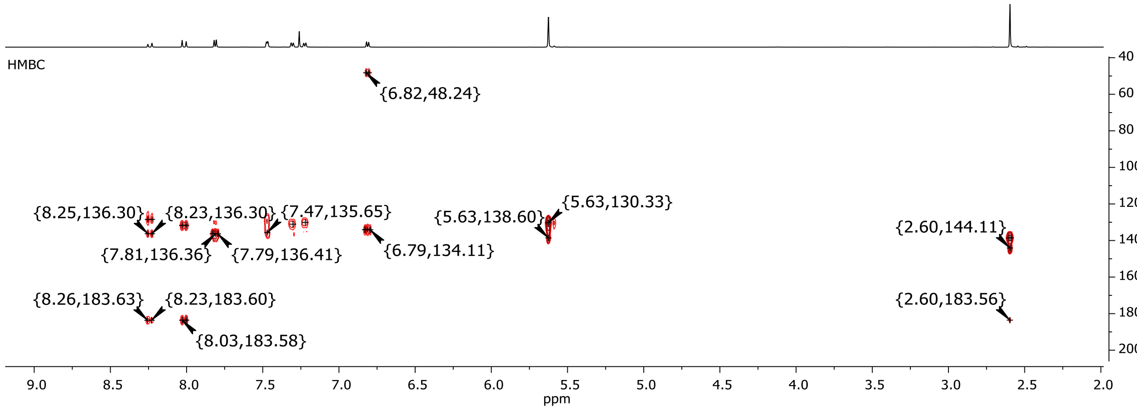

HMBC
{6.82,48.24}
{8.25,136.30}
{8.23,136.30}
{7.47,135.65}
{5.63,138.60}
{5.63,130.33}
{2.60,144.11}
{7.81,136.36}
{7.79,136.41}
{6.79,134.11}
{8.26,183.63}
{8.23,183.60}
{2.60,183.56}
{8.03,183.58}
ppm

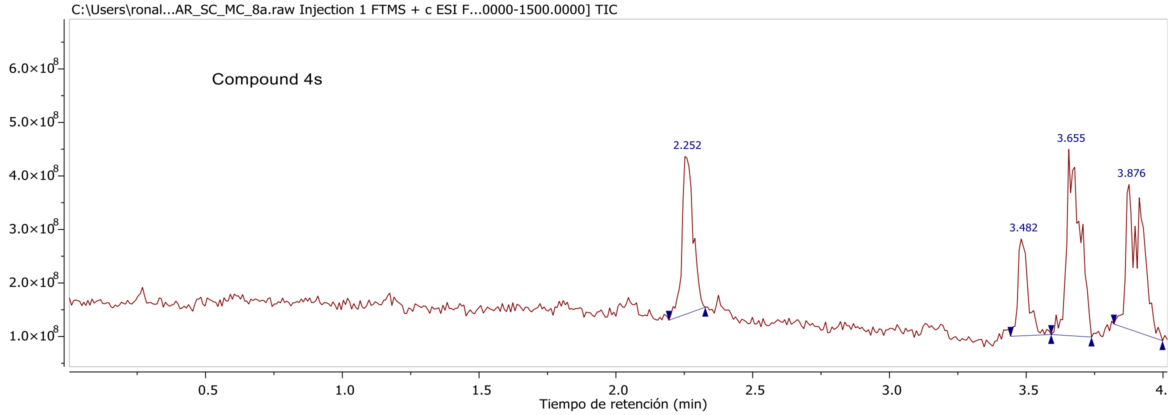
C:\Users\ronal...AR_SC_MC_8a.raw Injection 1 FTMS + c ESI F...0000-1500.0000] TIC
Compound 4s
2.252
3.482
3.655
3.876
6.0×10^8
5.0×10^8
4.0×10^8
3.0×10^8
2.0×10^8
1.0×10^8
0.5
1.0
1.5
2.0
2.5
3.0
3.5
4.
Tiempo de retención (min)

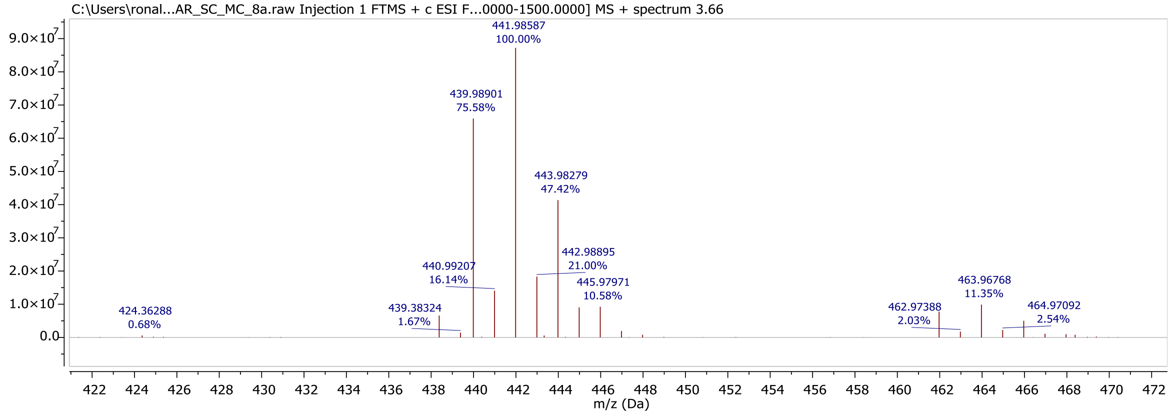
C:\Users\ronal...AR_SC_MC_8a.raw Injection 1 FTMS + c ESI F...0000-1500.0000] MS + spectrum 3.66
441.98587
100.00%
439.98901
75.58%
443.98279
47.42%
442.98895
21.00%
440.99207
16.14%
445.97971
10.58%
439.38324
1.67%
424.36288
0.68%
462.97388
2.03%
463.96768
11.35%
464.97092
2.54%
9.0×10^7
8.0×10^7
7.0×10^7
6.0×10^7
5.0×10^7
4.0×10^7
3.0×10^7
2.0×10^7
1.0×10^7
0.0
422
424
426
428
430
432
434
436
438
440
442
444
446
448
450
452
454
456
458
460
462
464
466
468
470
472
m/z (Da)

Compound 4t

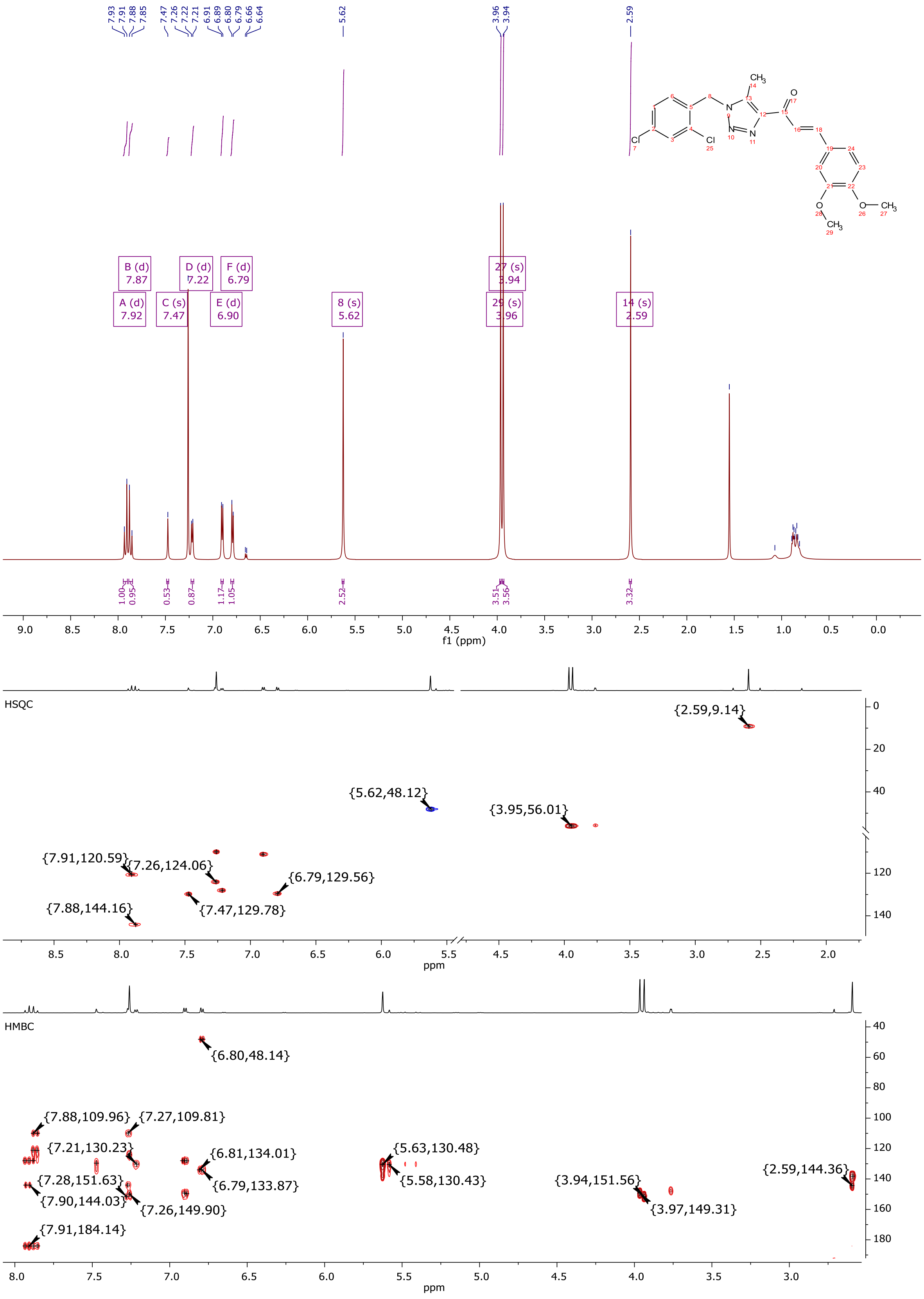

HSQC
{2.59,9.14}
{5.62,48.12}
{3.95,56.01}
{7.91,120.59}
{7.26,124.06}
{6.79,129.56}
{7.88,144.16}
{7.47,129.78}
HMBC
{6.80,48.14}
{7.88,109.96}
{7.27,109.81}
{7.21,130.23}
{6.81,134.01}
{5.63,130.48}
{7.28,151.63}
{6.79,133.87}
{5.58,130.43}
{3.94,151.56}
{2.59,144.36}
{7.90,144.03}
{7.26,149.90}
{3.97,149.31}
{7.91,184.14}
f1 (ppm)
ppm

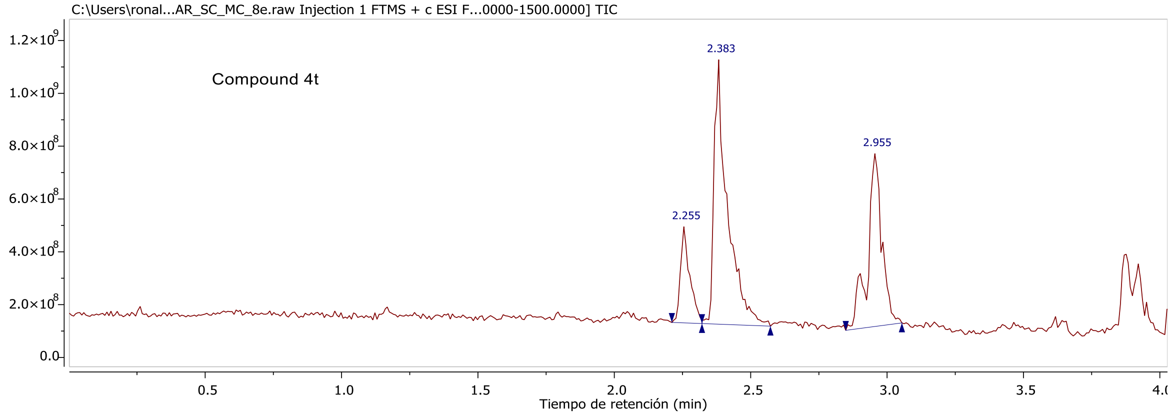
C:\Users\ronal...AR_SC_MC_8e.raw Injection 1 FTMS + c ESI F...0000-1500.0000] TIC
Compound 4t
2.383
2.955
2.255
Tiempo de retención (min)

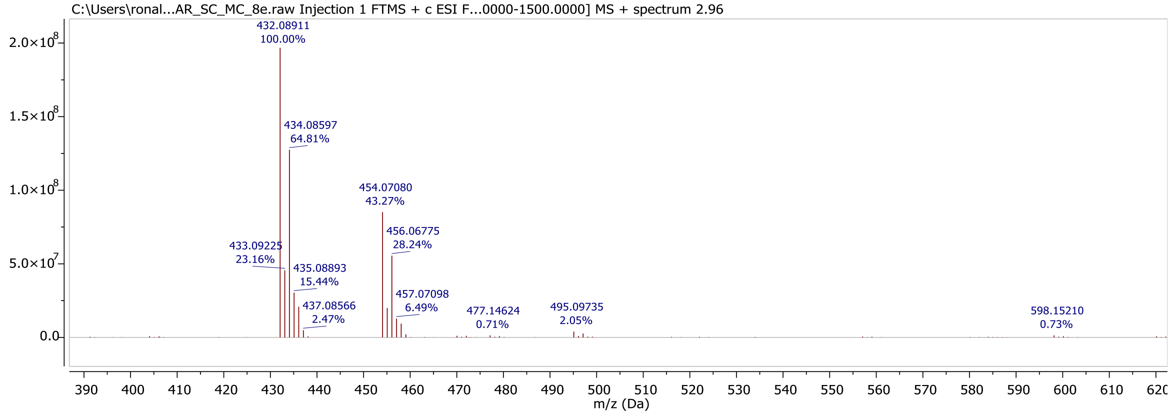
C:\Users\ronal...AR_SC_MC_8e.raw Injection 1 FTMS + c ESI F...0000-1500.0000] MS + spectrum 2.96
432.08911
100.00%
434.08597
64.81%
454.07080
43.27%
456.06775
28.24%
433.09225
23.16%
435.08893
15.44%
437.08566
2.47%
457.07098
6.49%
477.14624
0.71%
495.09735
2.05%
598.15210
0.73%
m/z (Da)

Compound 4v

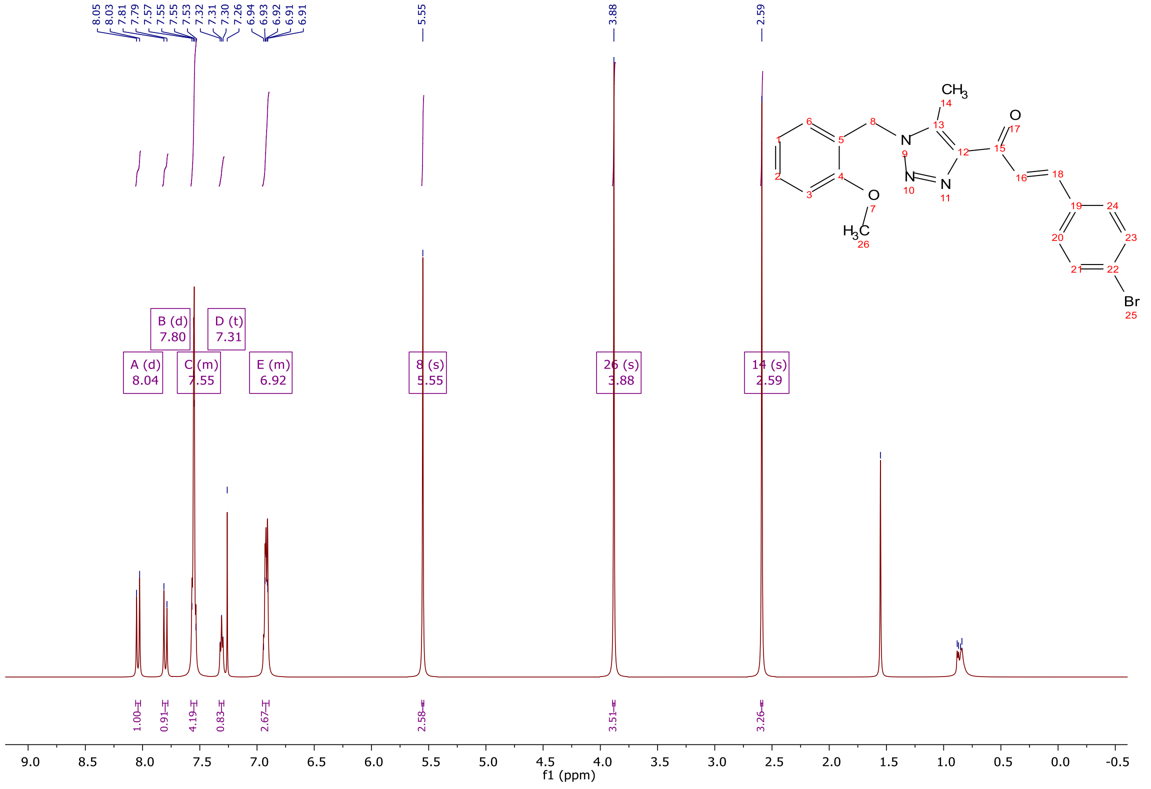

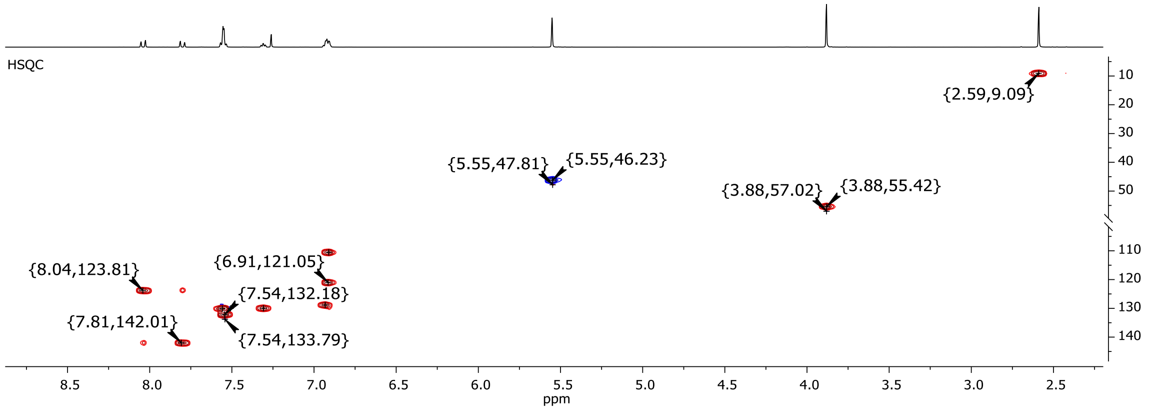

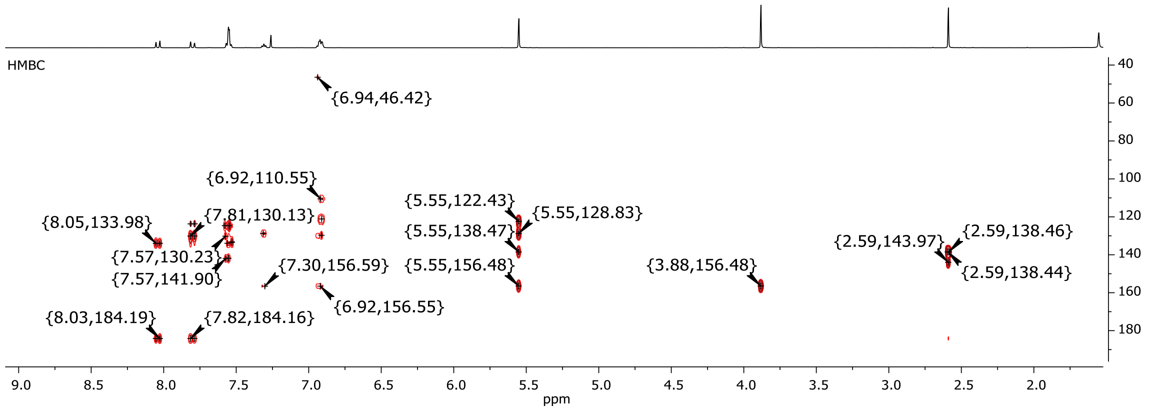

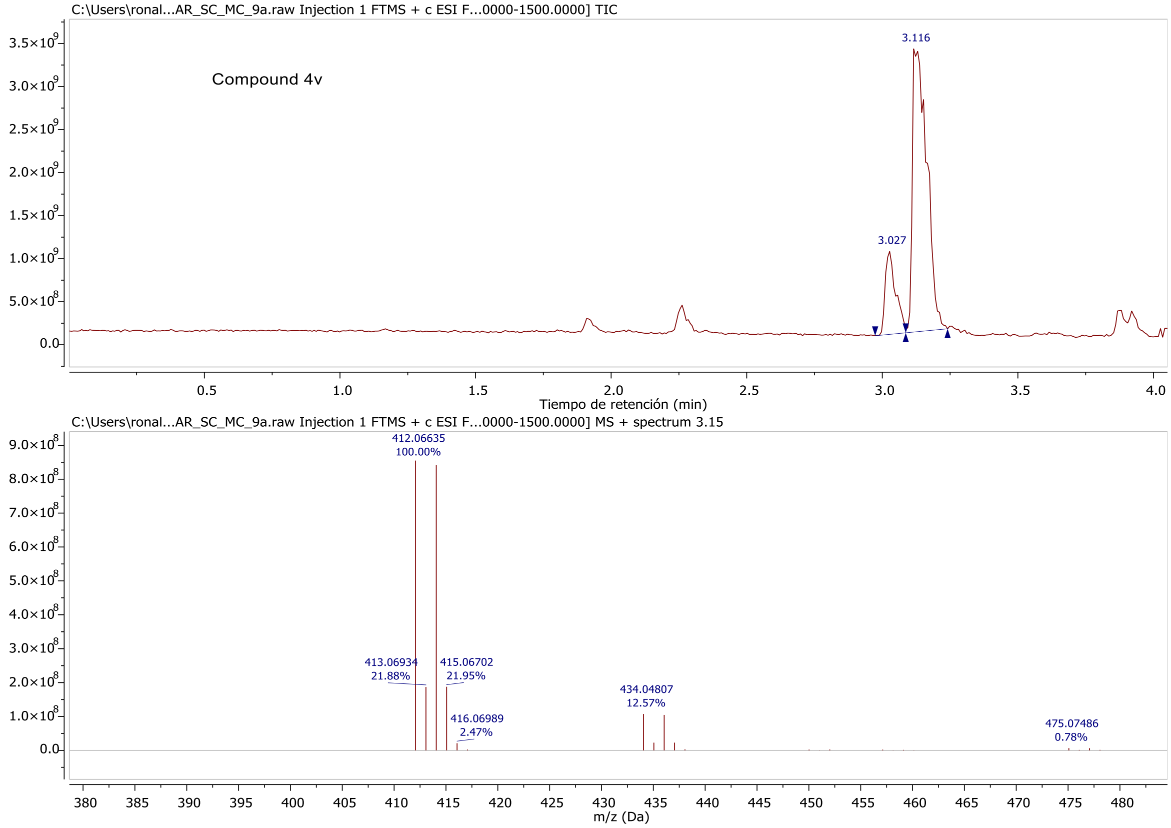
C:\Users\ronal...AR_SC_MC_9a.raw Injection 1 FTMS + c ESI F...0000-1500.0000] TIC
Compound 4v
3.116
3.027
Tiempo de retención (min)
C:\Users\ronal...AR_SC_MC_9a.raw Injection 1 FTMS + c ESI F...0000-1500.0000] MS + spectrum 3.15
412.06635
100.00%
413.06934
21.88%
415.06702
21.95%
416.06989
2.47%
434.04807
12.57%
475.07486
0.78%
m/z (Da)
